# Supplementary figures and images for: NAT10 inhibits ferroptosis and promotes the progression of renal clear cell carcinoma by regulating the NFE2L1-GPX4 signaling pathway (part 1 of 2)
Source: PeerJ. 2025 Oct 31;13:e20224. doi: 10.7717/peerj.20224 (PMC12581918; doi:10.7717/peerj.20224)

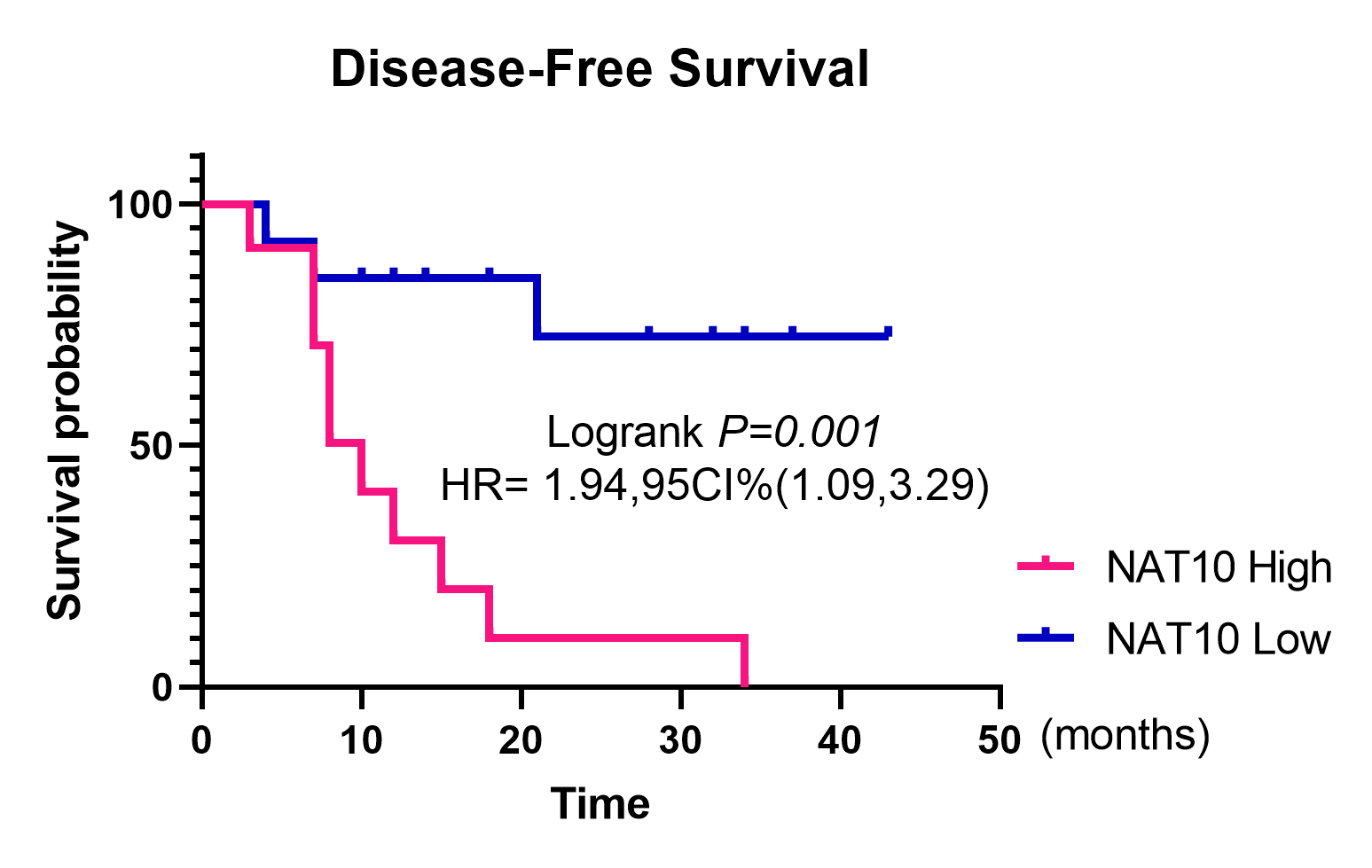

Supplement: Supplemental Information 2 [file peerj-13-20224-s002.zip › FIGURE1/fig-1G/Fig-1G.tif]

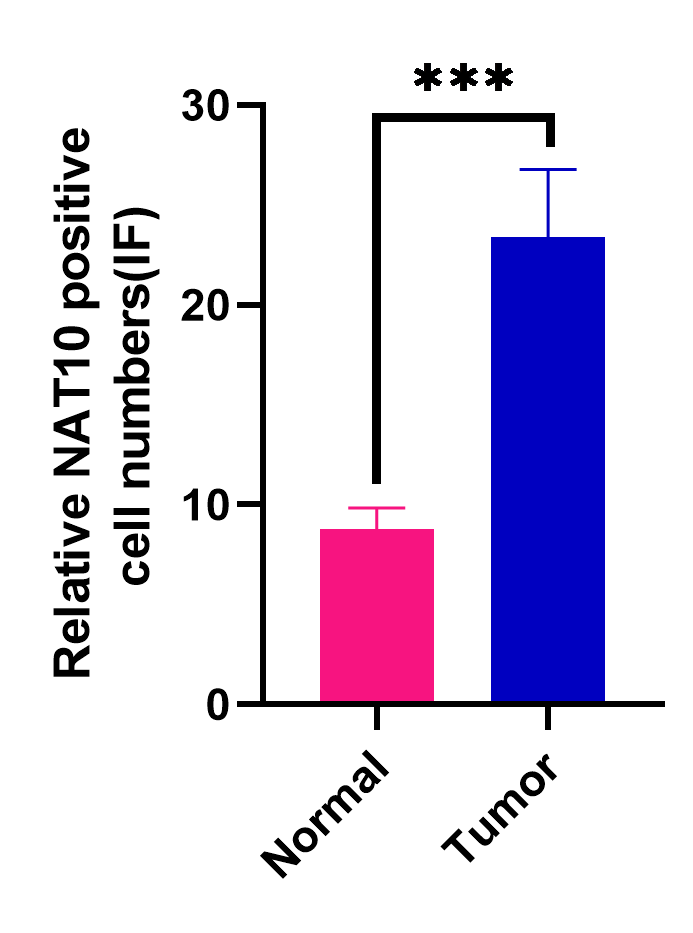

Supplement: Supplemental Information 2 [file peerj-13-20224-s002.zip › FIGURE1/fig1A-IF/fig-1C.tif]

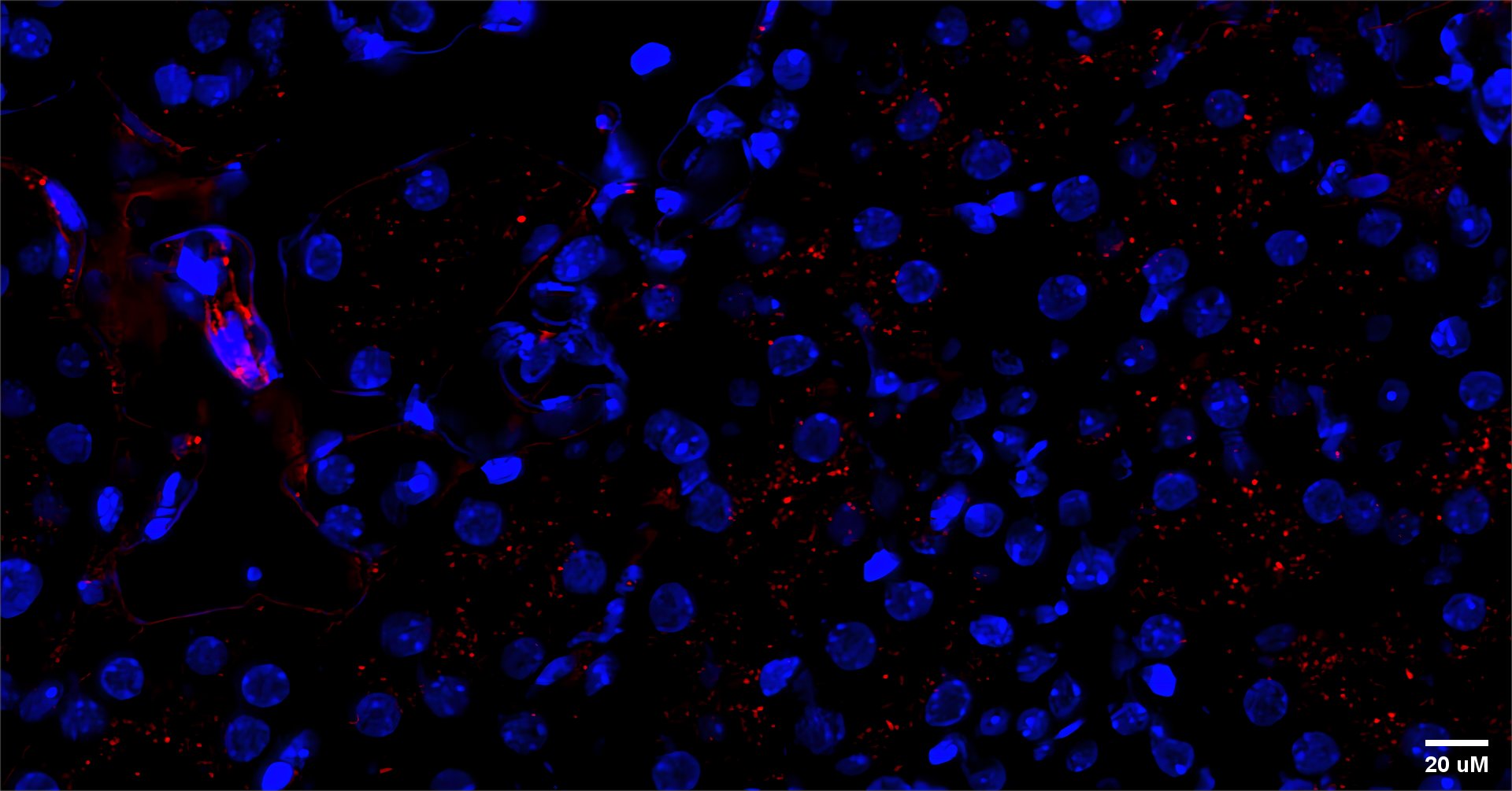

Supplement: Supplemental Information 2 [file peerj-13-20224-s002.zip › FIGURE1/fig1A-IF/Normal.jpg]

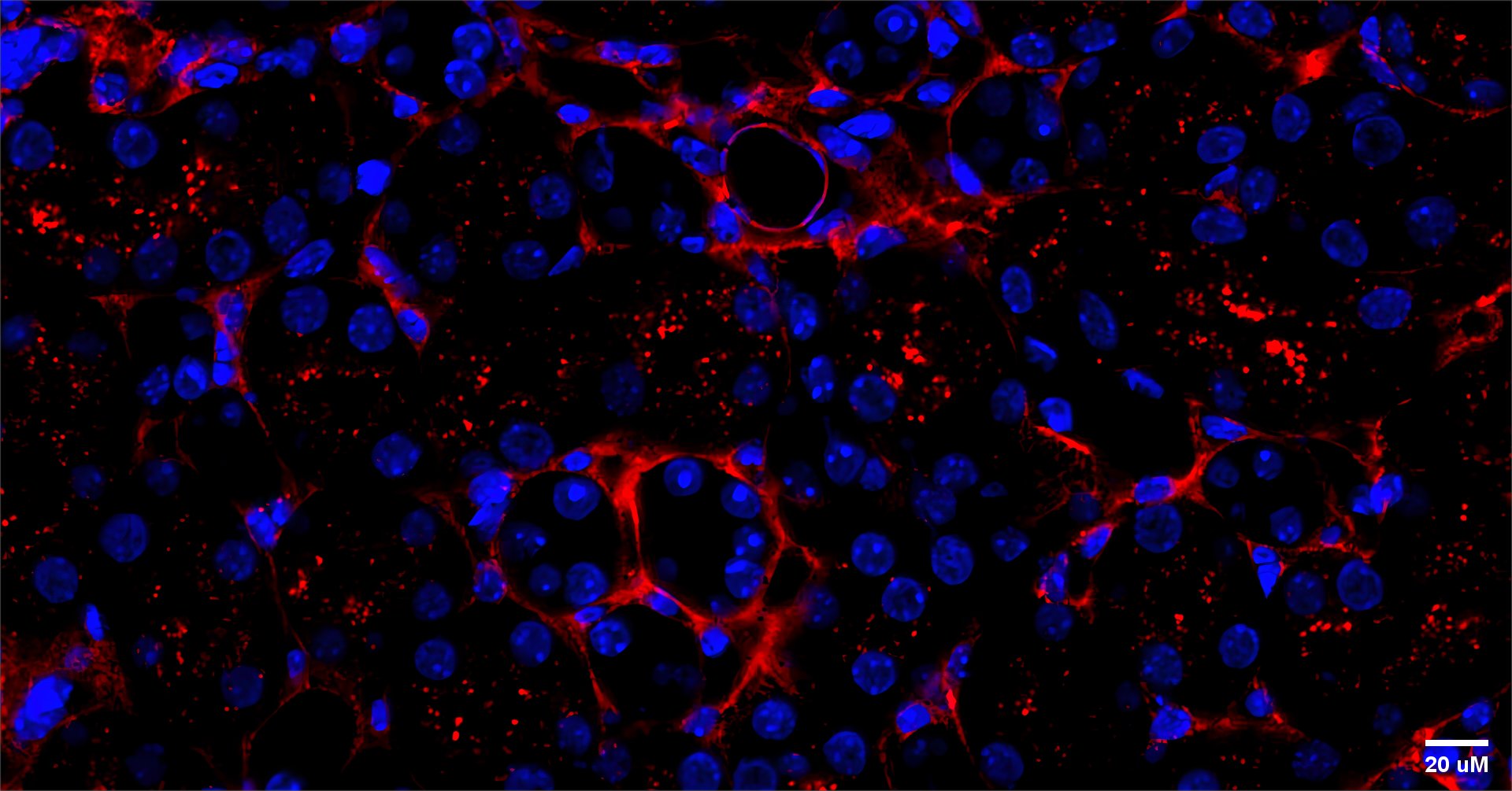

Supplement: Supplemental Information 2 [file peerj-13-20224-s002.zip › FIGURE1/fig1A-IF/Tumor.jpg]

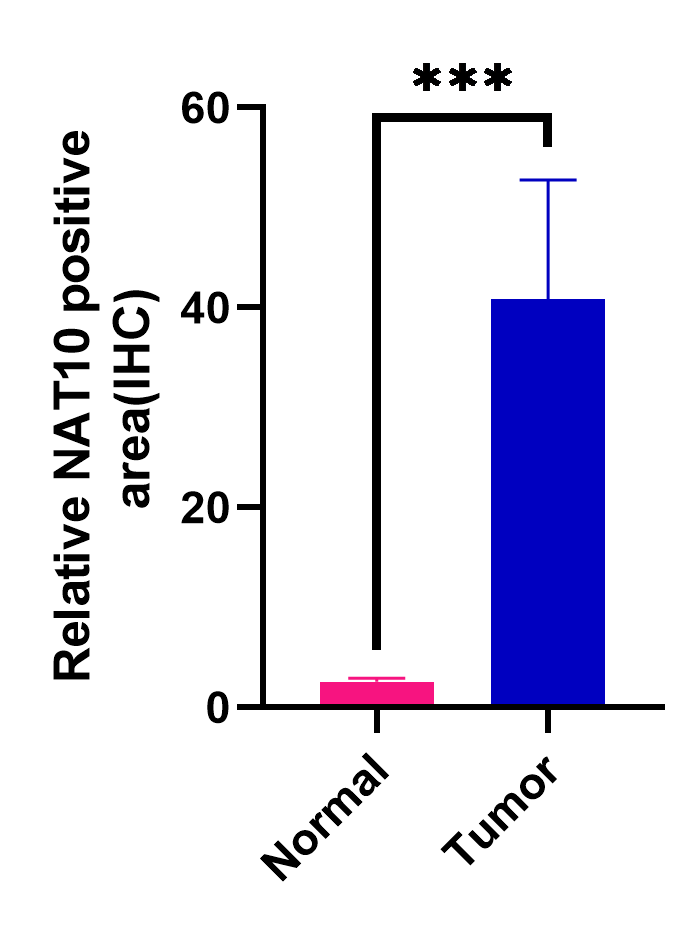

Supplement: Supplemental Information 2 [file peerj-13-20224-s002.zip › FIGURE1/fig1A-IHC/fig-1B.tif]

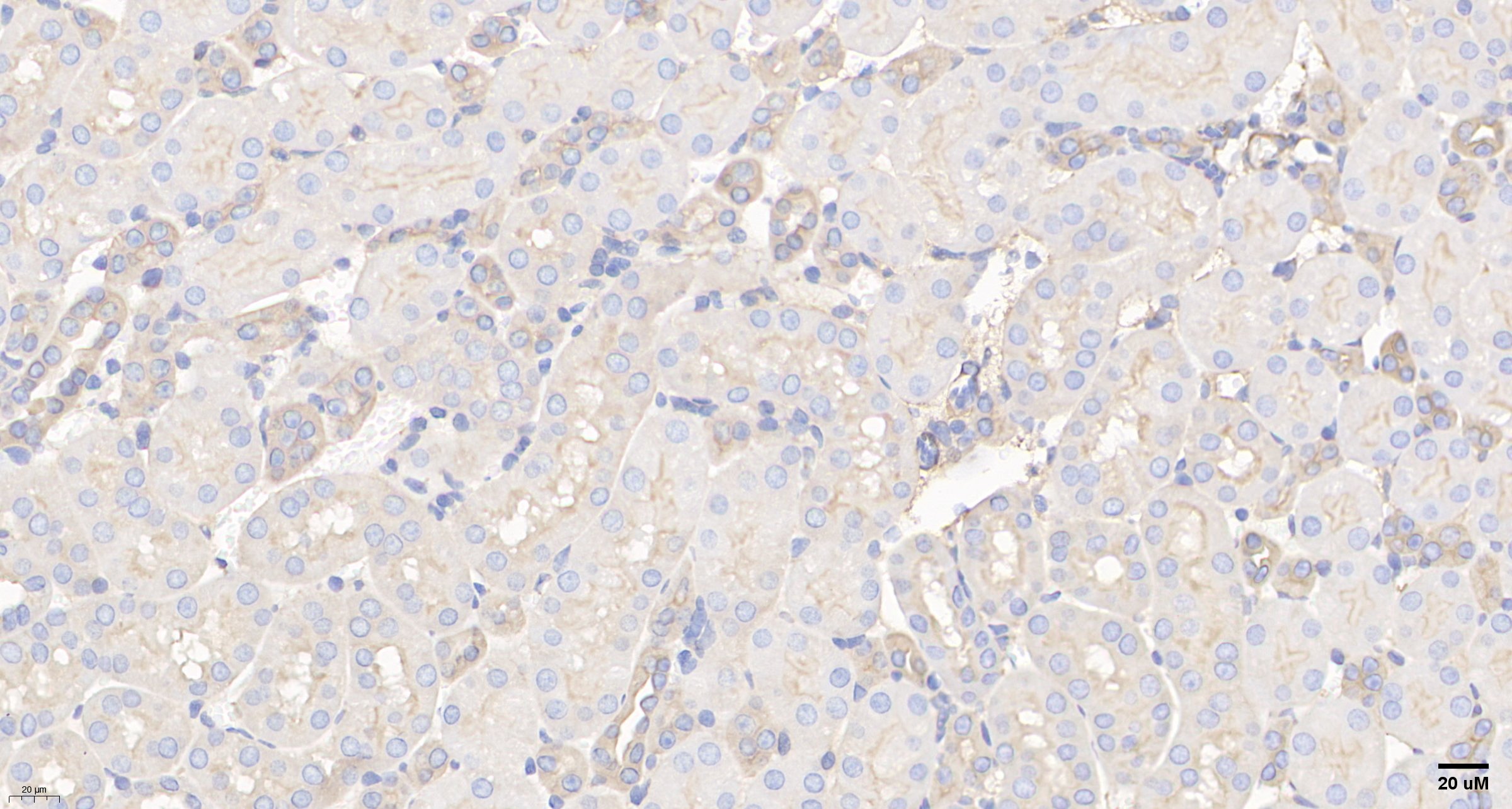

Supplement: Supplemental Information 2 [file peerj-13-20224-s002.zip › FIGURE1/fig1A-IHC/Fig.1A(N1).jpg]

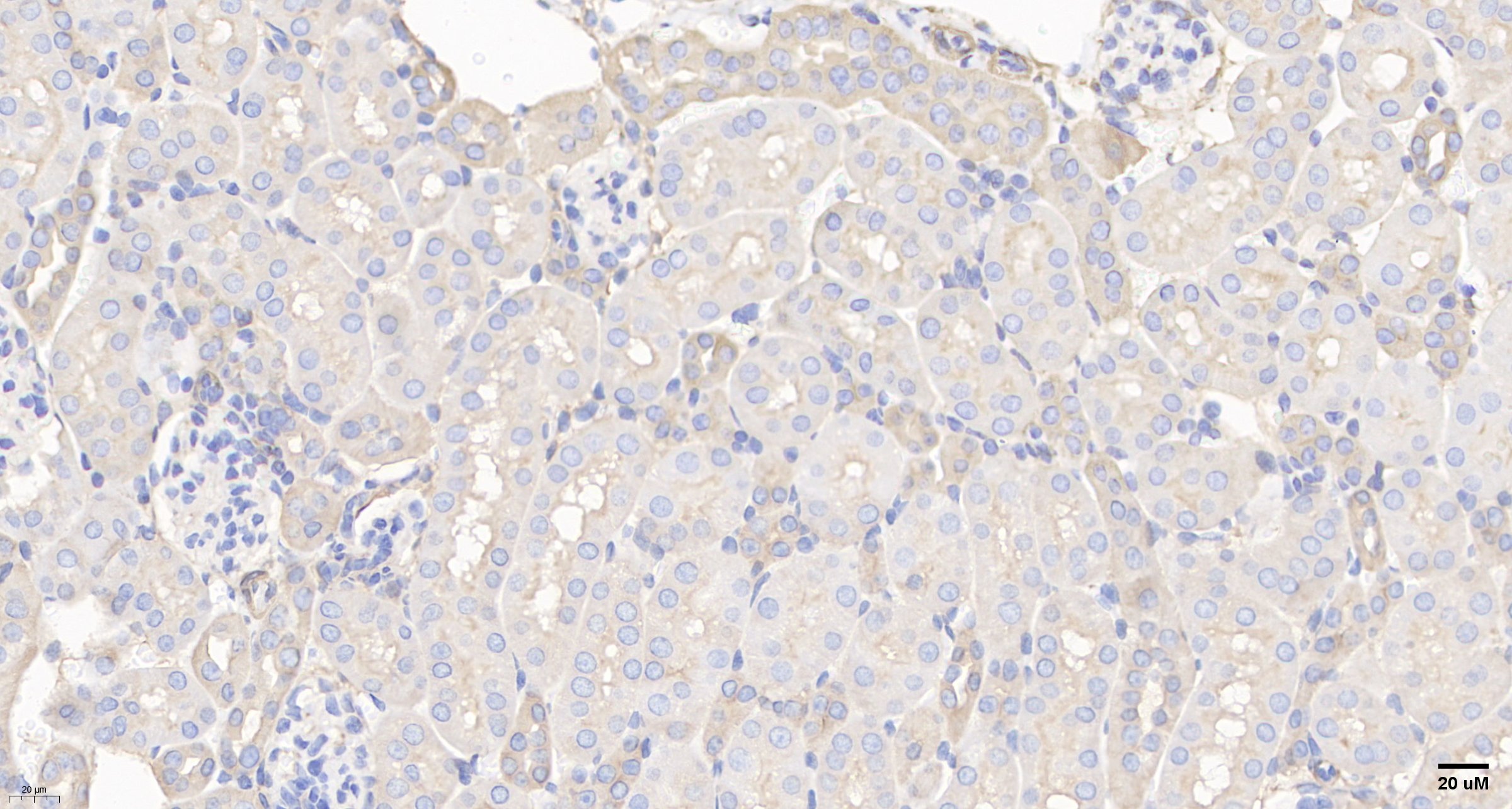

Supplement: Supplemental Information 2 [file peerj-13-20224-s002.zip › FIGURE1/fig1A-IHC/Fig.1A(N2).jpg]

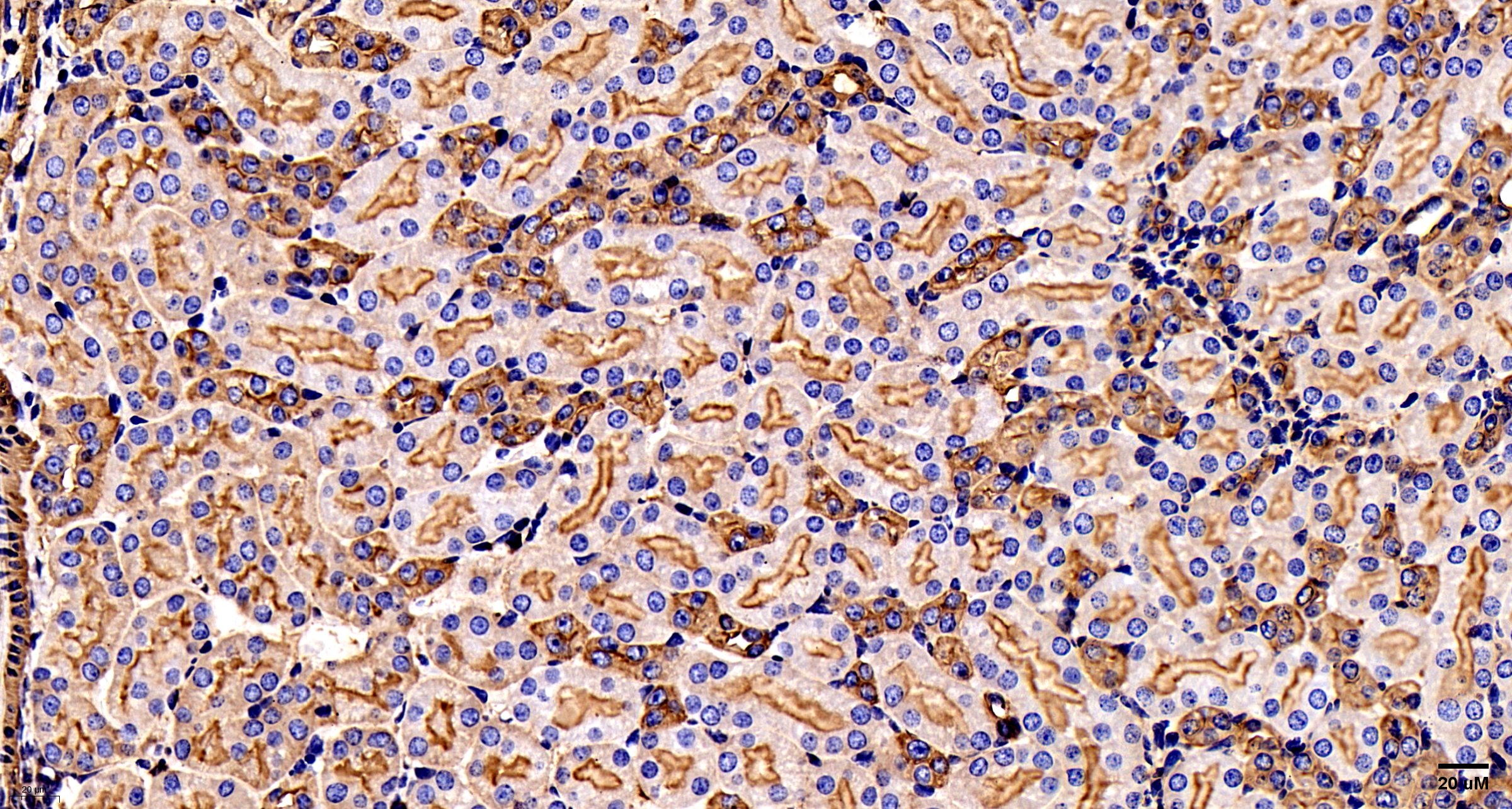

Supplement: Supplemental Information 2 [file peerj-13-20224-s002.zip › FIGURE1/fig1A-IHC/Fig.1A(T1).jpg]

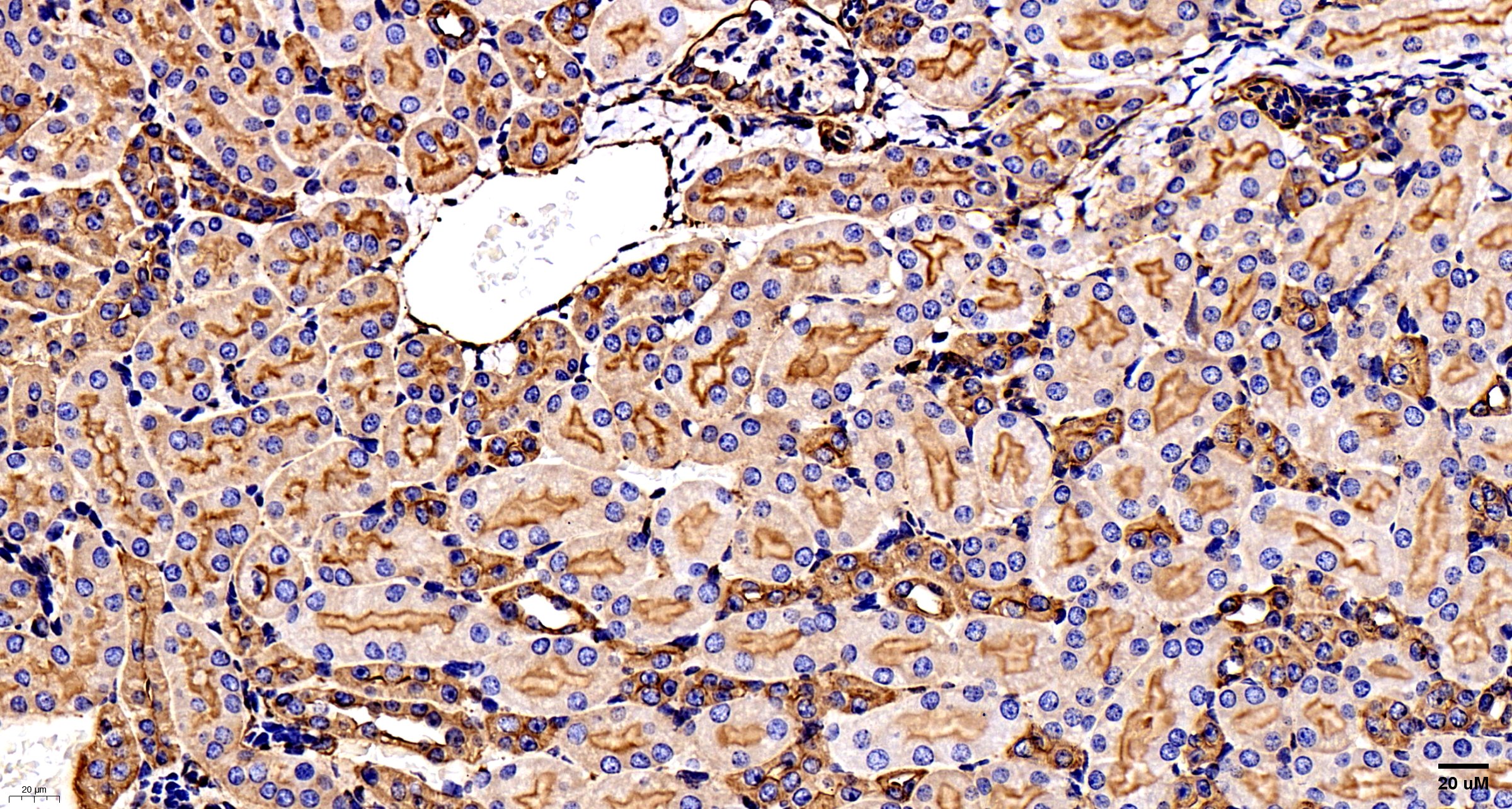

Supplement: Supplemental Information 2 [file peerj-13-20224-s002.zip › FIGURE1/fig1A-IHC/Fig.1A(T2).jpg]

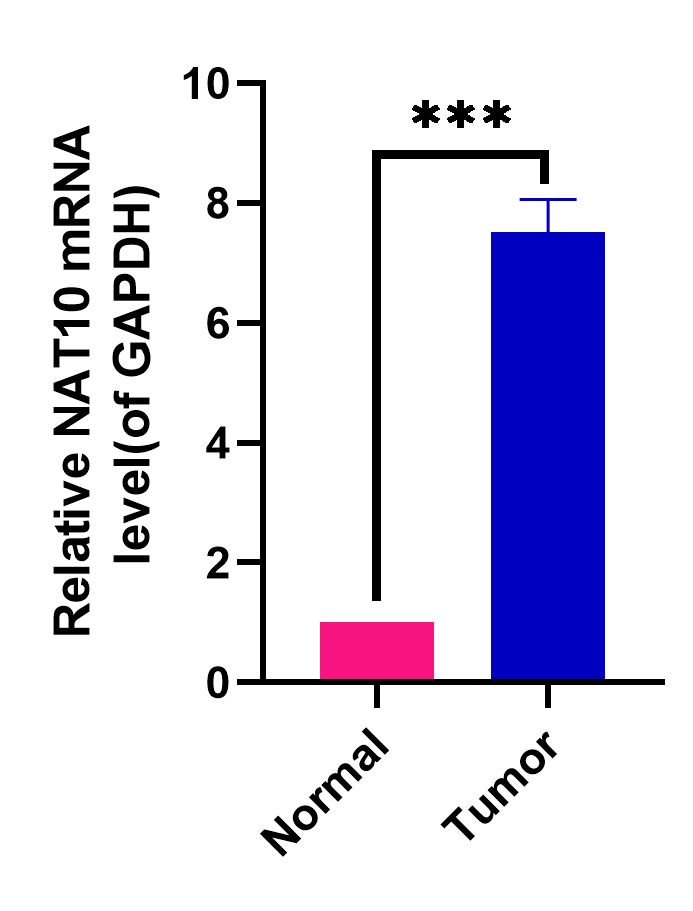

Supplement: Supplemental Information 2 [file peerj-13-20224-s002.zip › FIGURE1/PCR/fig-1D.tif]

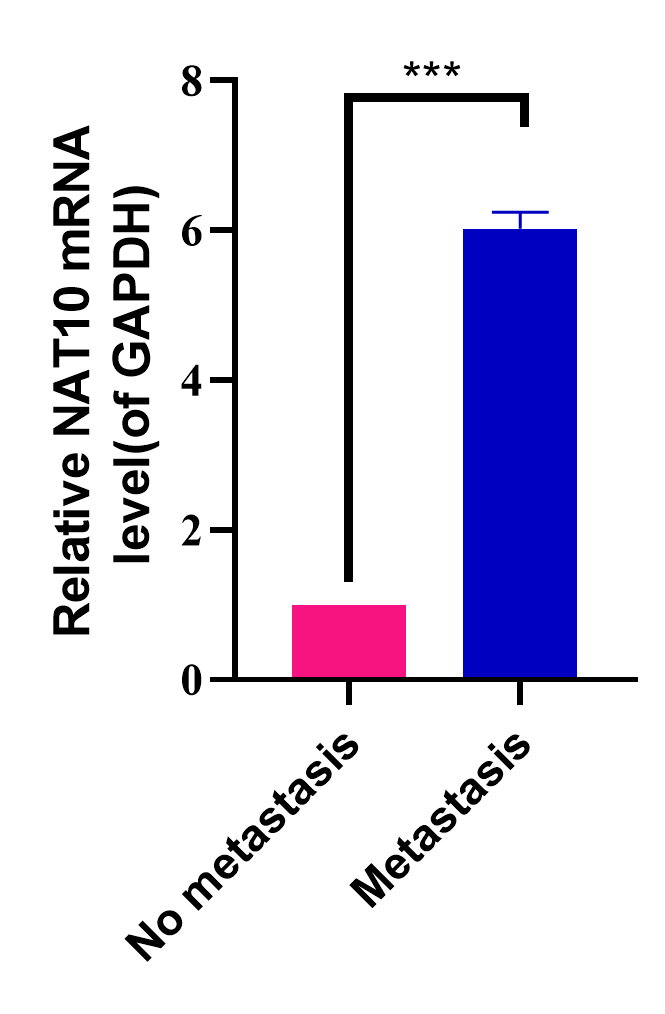

Supplement: Supplemental Information 2 [file peerj-13-20224-s002.zip › FIGURE1/PCR/fig-1E.tif]

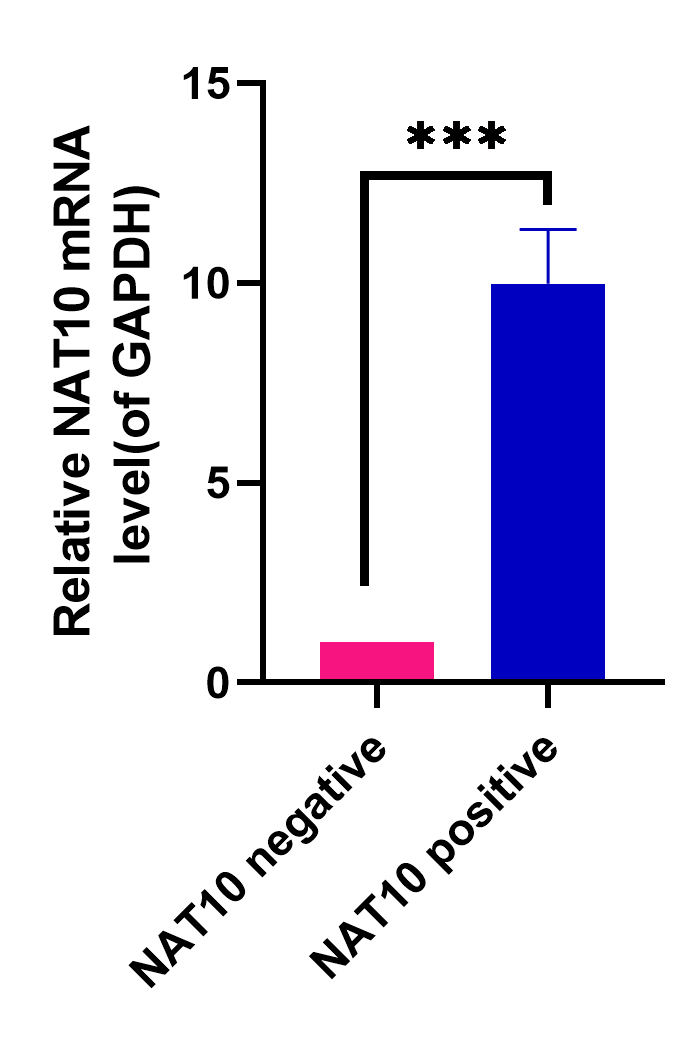

Supplement: Supplemental Information 2 [file peerj-13-20224-s002.zip › FIGURE1/PCR/fig-1F.tif]

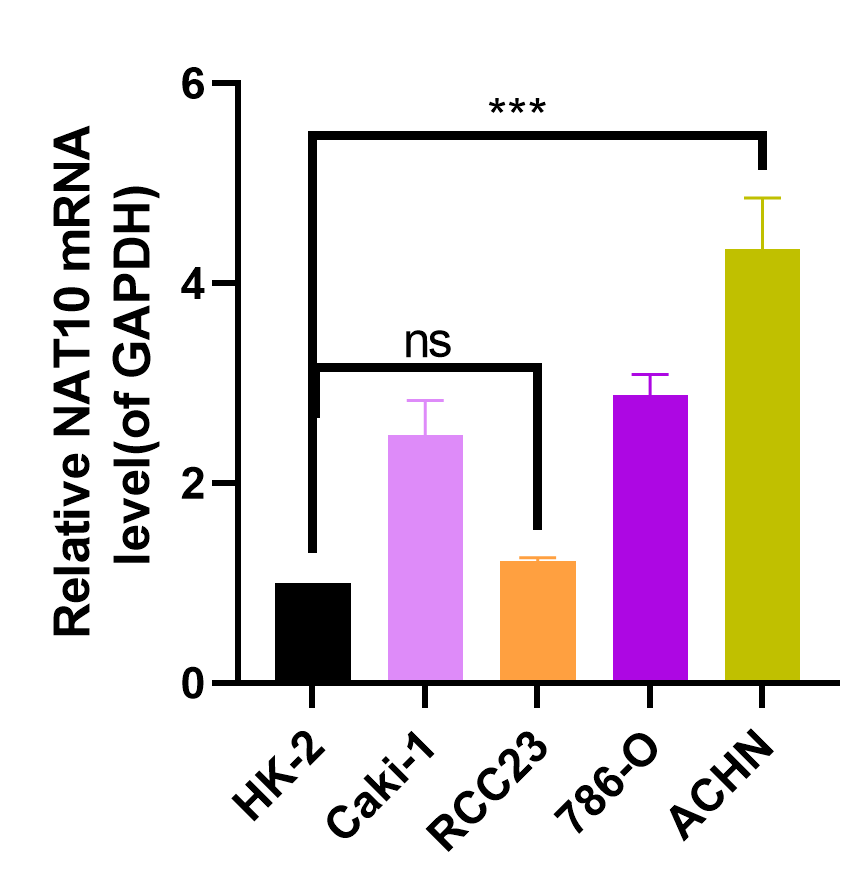

Supplement: Supplemental Information 3 [file peerj-13-20224-s003.zip › FIGURE2/FIG-2A/FIG-2A.tif]

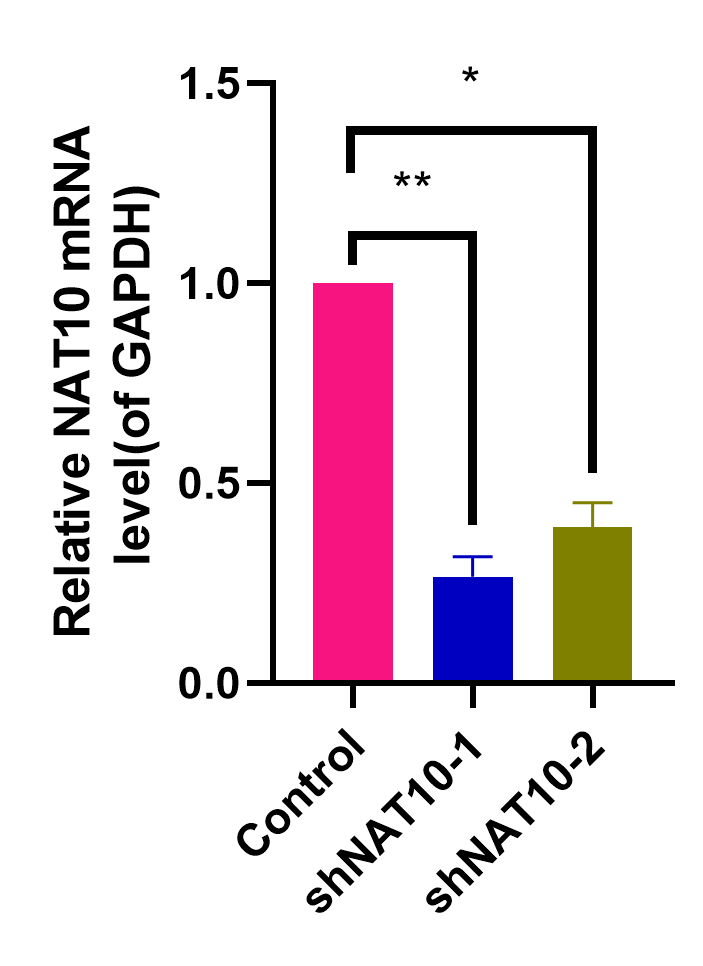

Supplement: Supplemental Information 3 [file peerj-13-20224-s003.zip › FIGURE2/FIG-2B-C/FIG-2B.tif]

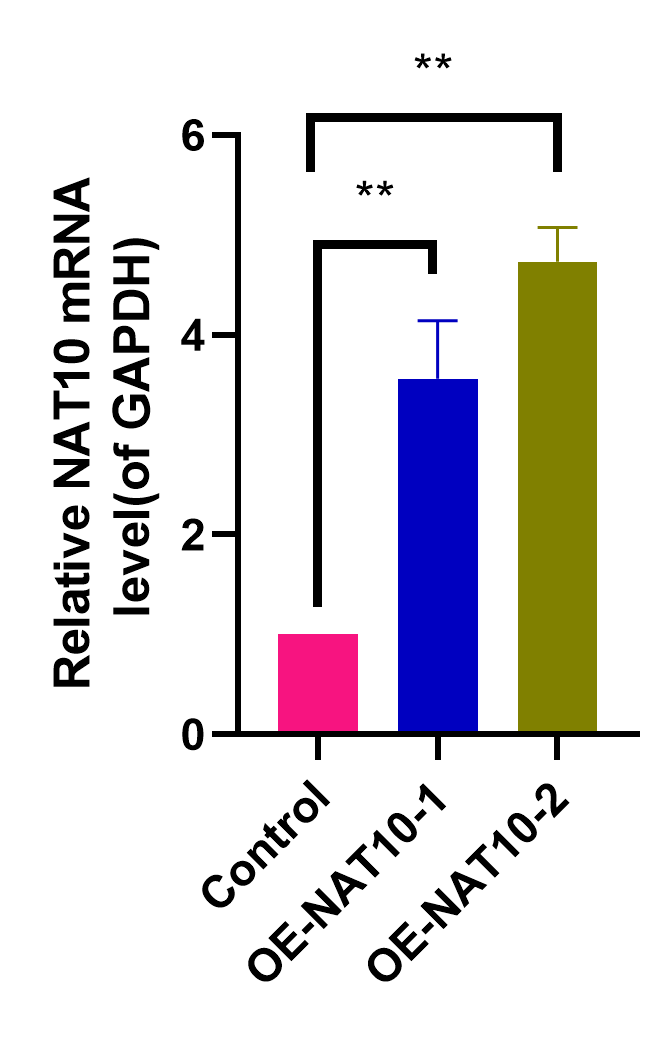

Supplement: Supplemental Information 3 [file peerj-13-20224-s003.zip › FIGURE2/FIG-2B-C/FIG-2C.tif]

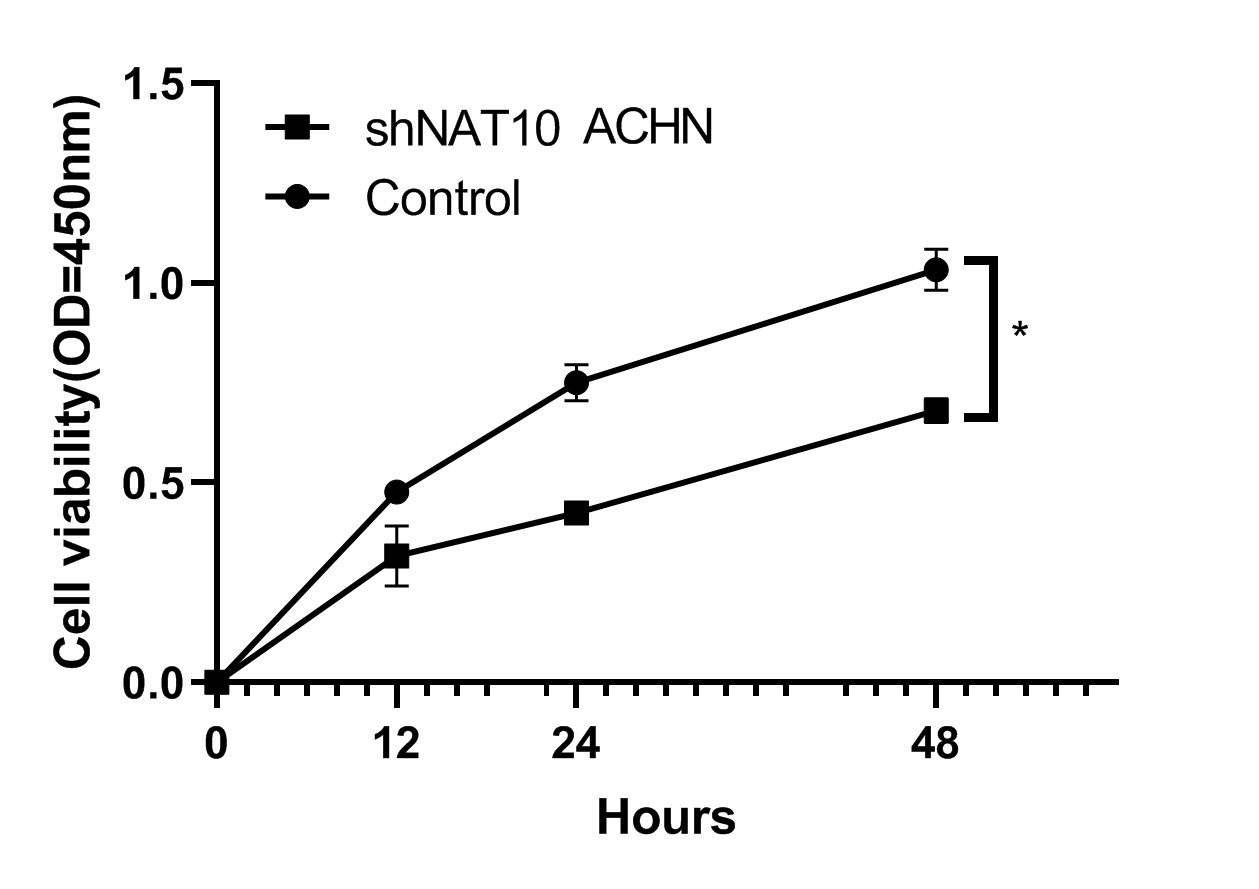

Supplement: Supplemental Information 3 [file peerj-13-20224-s003.zip › FIGURE2/FIG-2D-E/FIG-2D.tif]

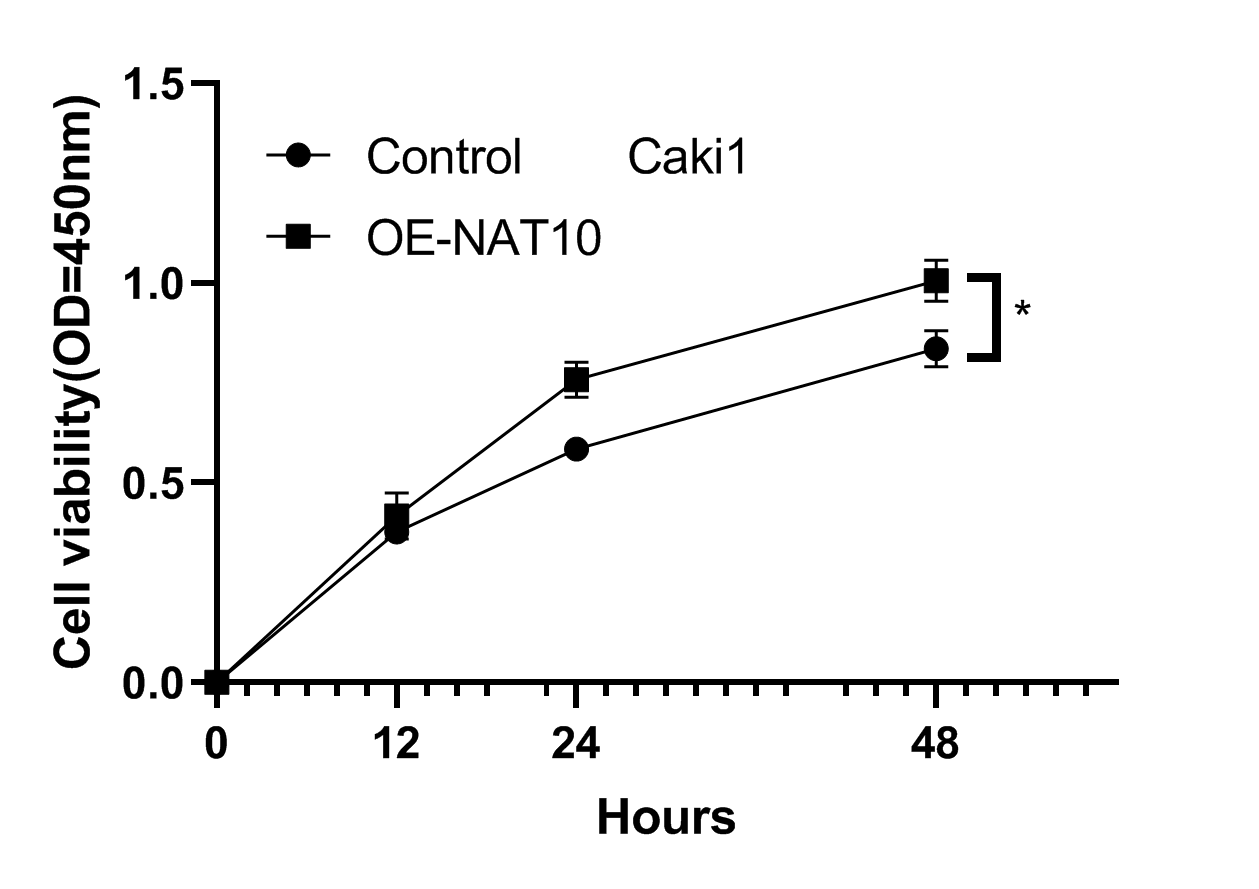

Supplement: Supplemental Information 3 [file peerj-13-20224-s003.zip › FIGURE2/FIG-2D-E/FIG-2E.tif]

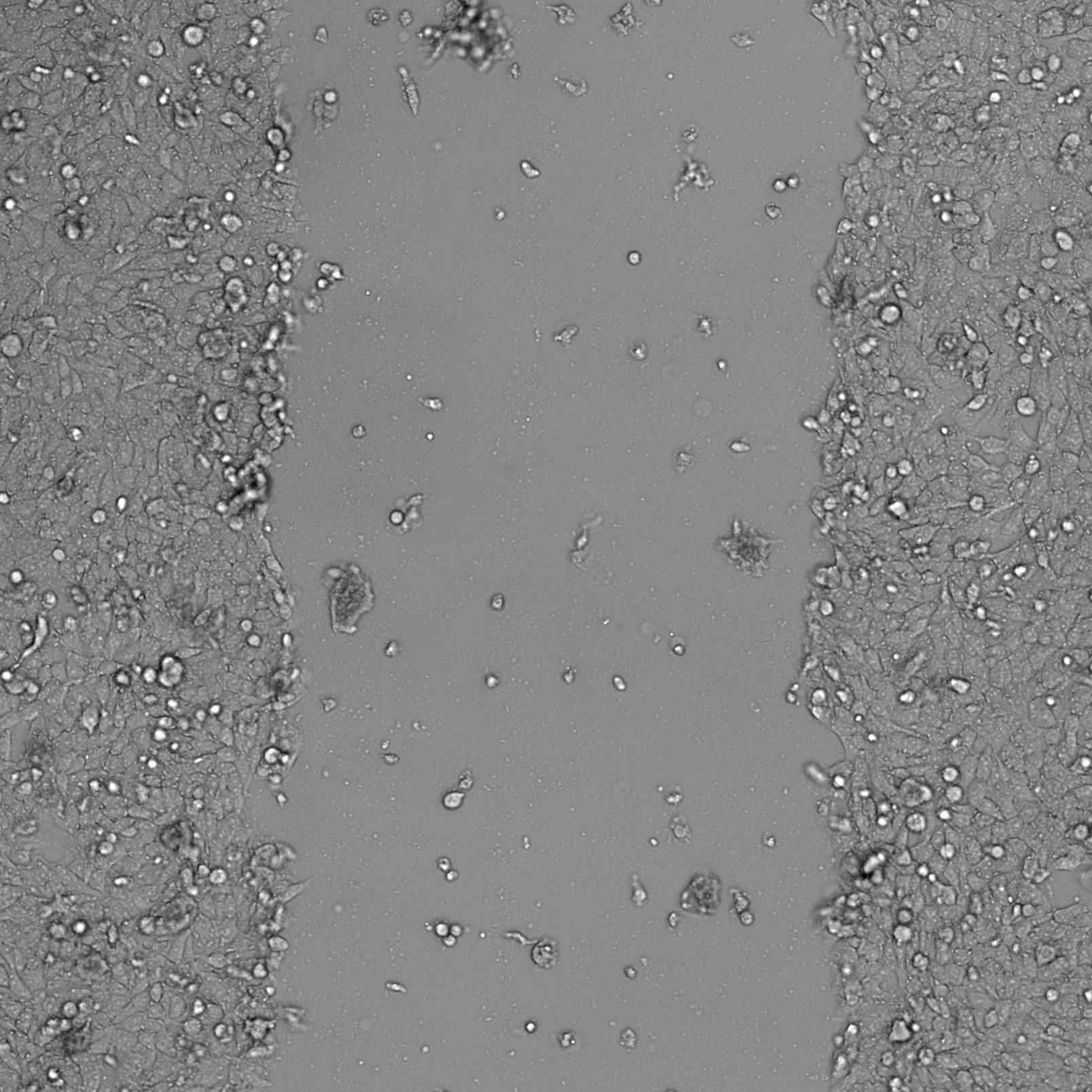

Supplement: Supplemental Information 3 [file peerj-13-20224-s003.zip › FIGURE2/FIG-2F-H/ACHN/0/Control.jpg]

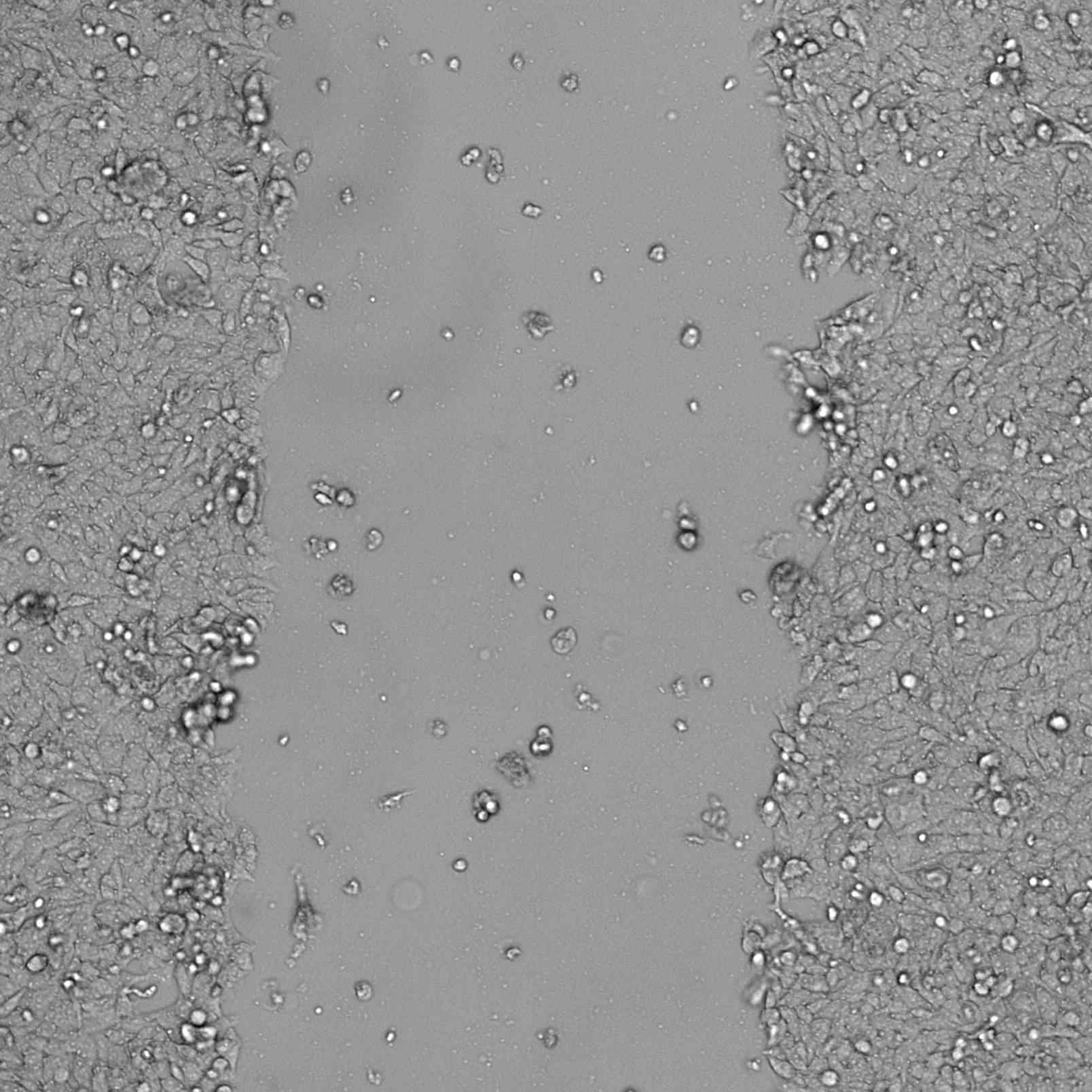

Supplement: Supplemental Information 3 [file peerj-13-20224-s003.zip › FIGURE2/FIG-2F-H/ACHN/0/shNAT10.jpg]

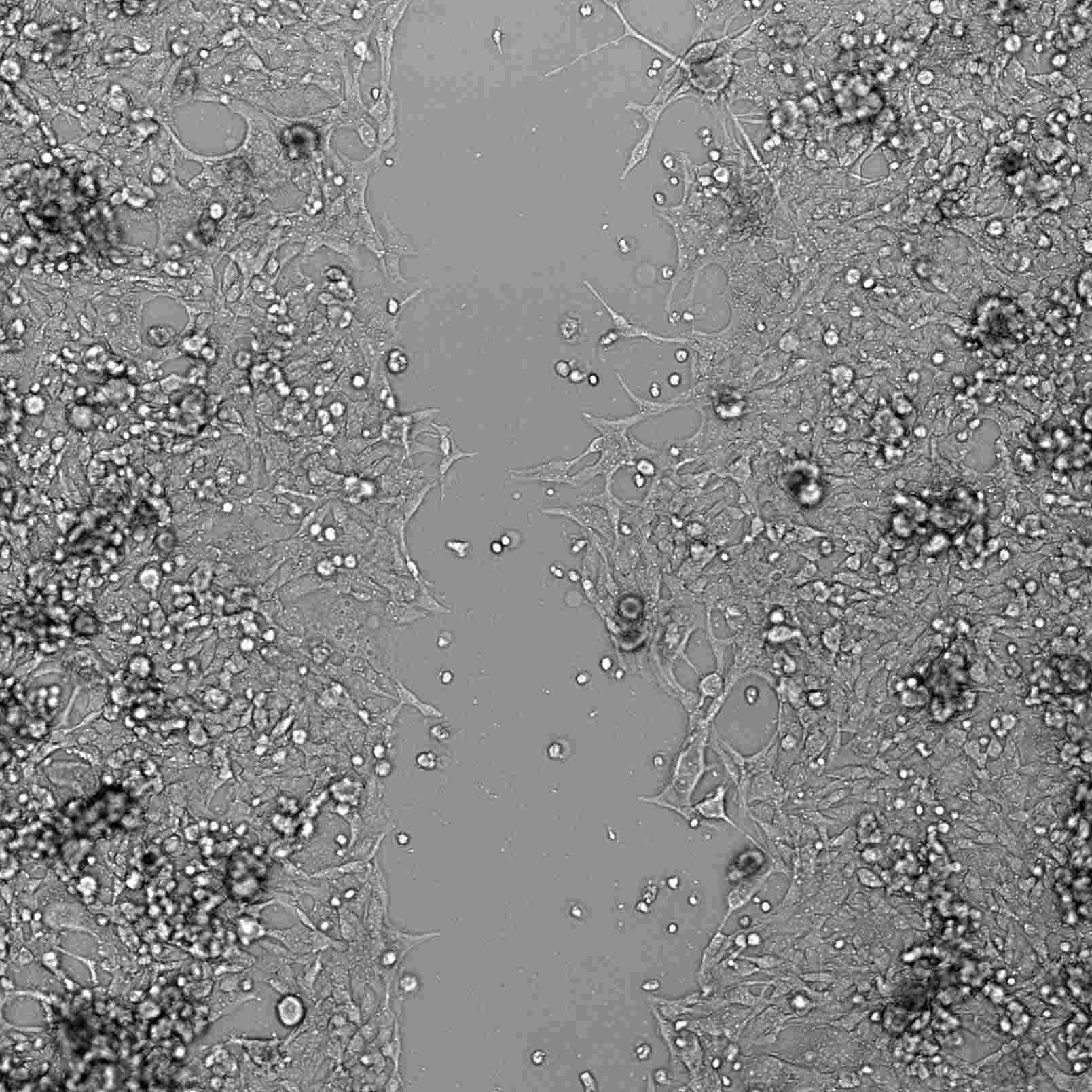

Supplement: Supplemental Information 3 [file peerj-13-20224-s003.zip › FIGURE2/FIG-2F-H/ACHN/24/Control.jpg]

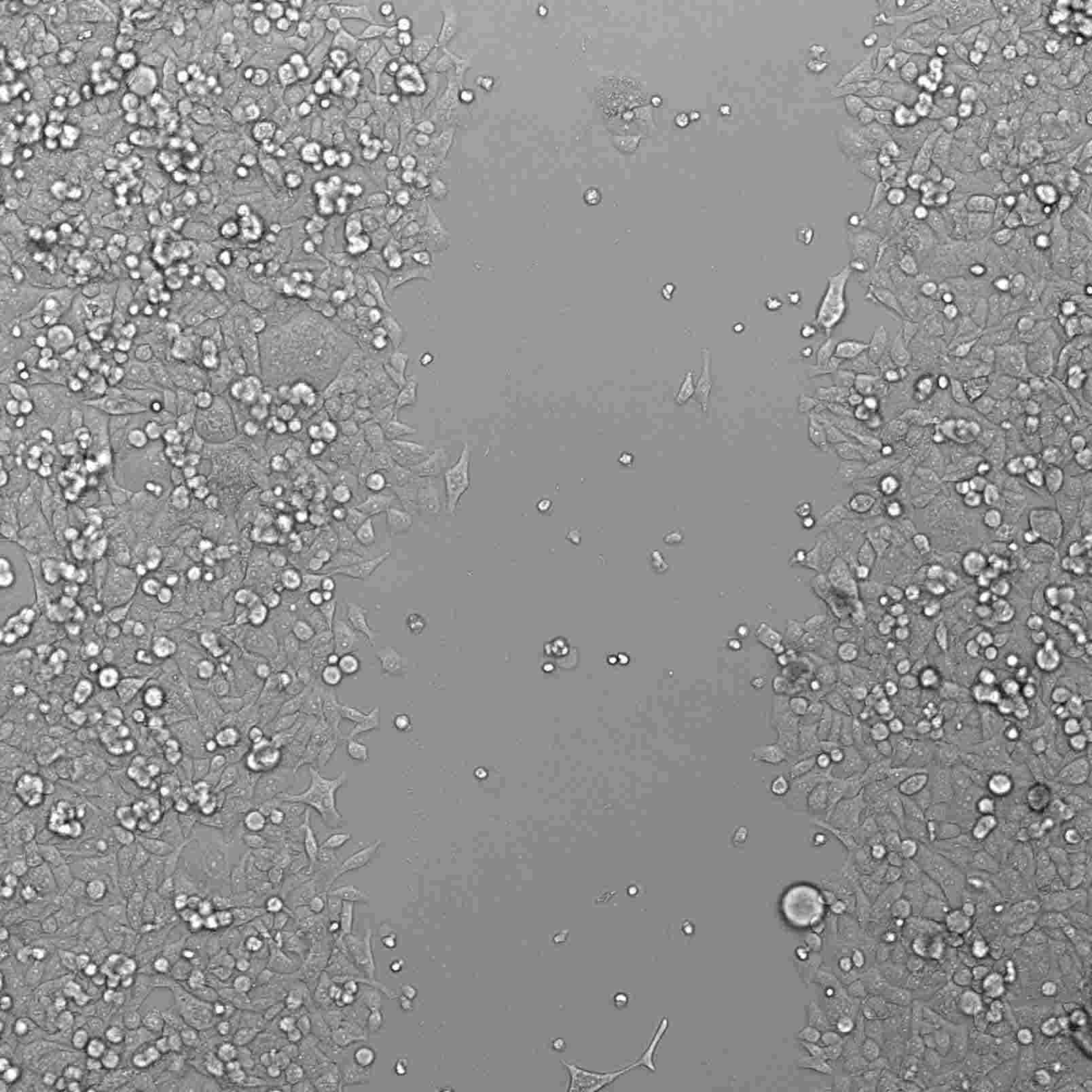

Supplement: Supplemental Information 3 [file peerj-13-20224-s003.zip › FIGURE2/FIG-2F-H/ACHN/24/shNAT10.jpg]

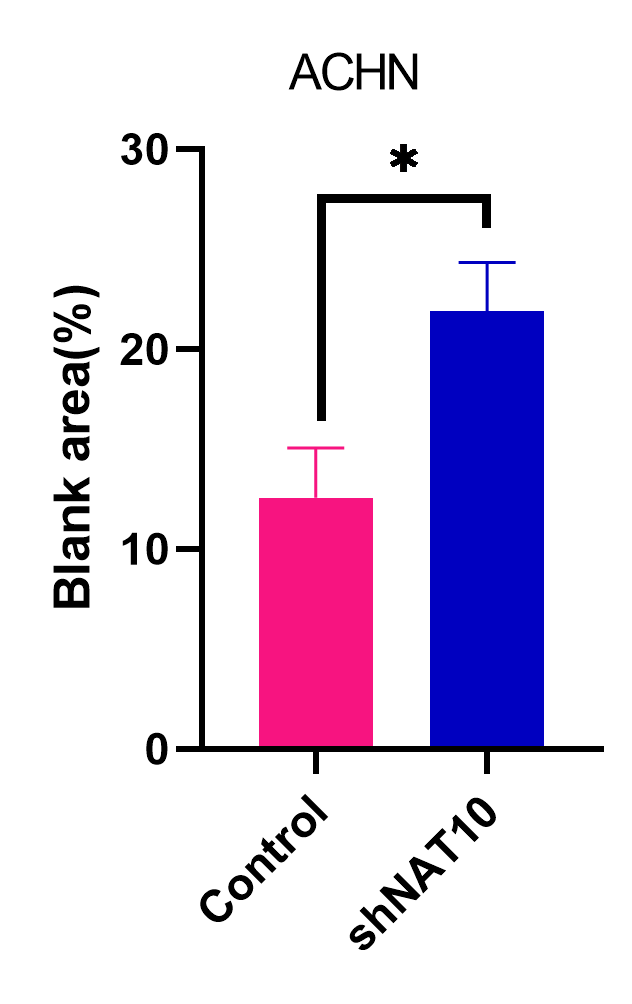

Supplement: Supplemental Information 3 [file peerj-13-20224-s003.zip › FIGURE2/FIG-2F-H/ACHN/FIG-2G.tif]

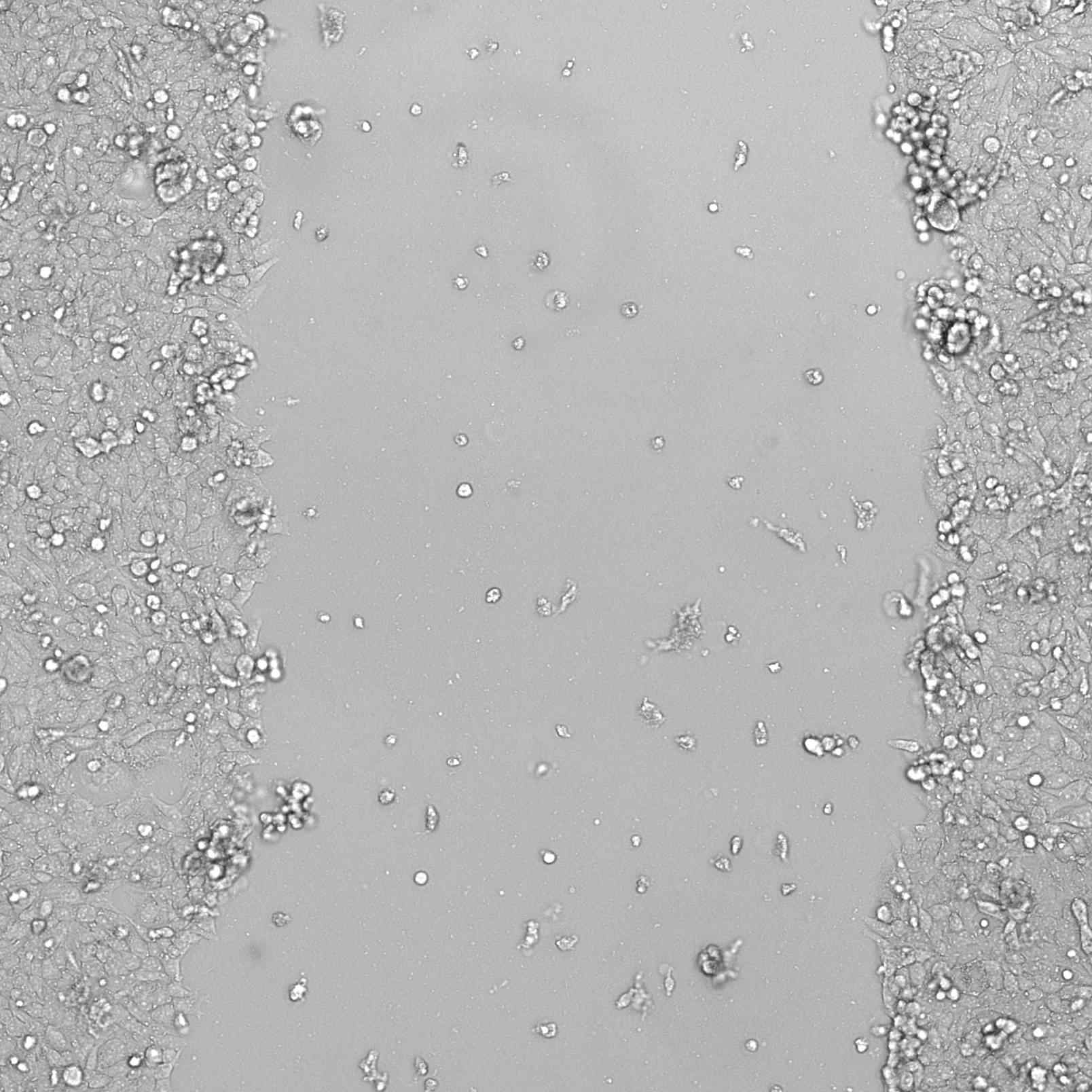

Supplement: Supplemental Information 3 [file peerj-13-20224-s003.zip › FIGURE2/FIG-2F-H/CAKi1/0/Control.jpg]

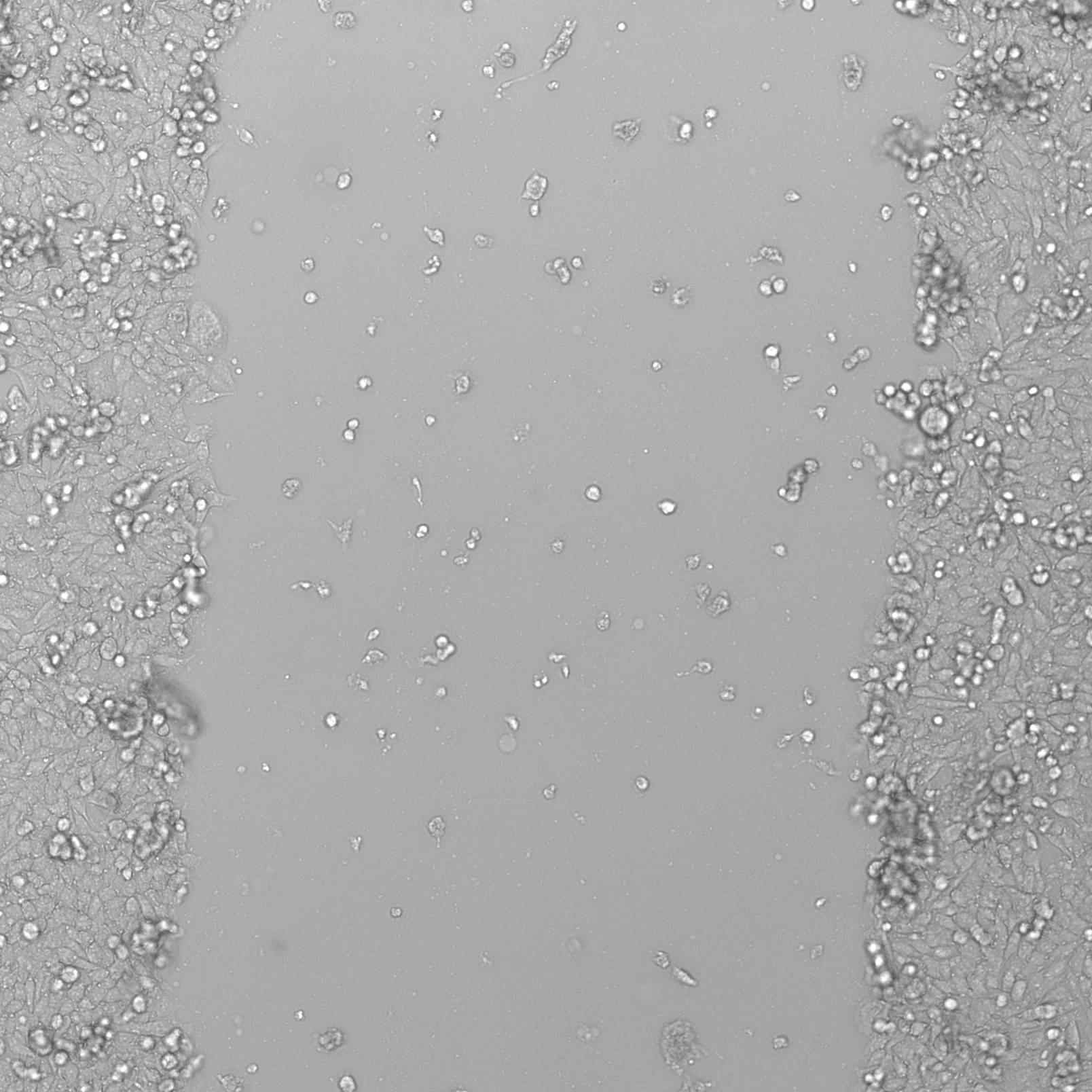

Supplement: Supplemental Information 3 [file peerj-13-20224-s003.zip › FIGURE2/FIG-2F-H/CAKi1/0/OE-NAT10.jpg]

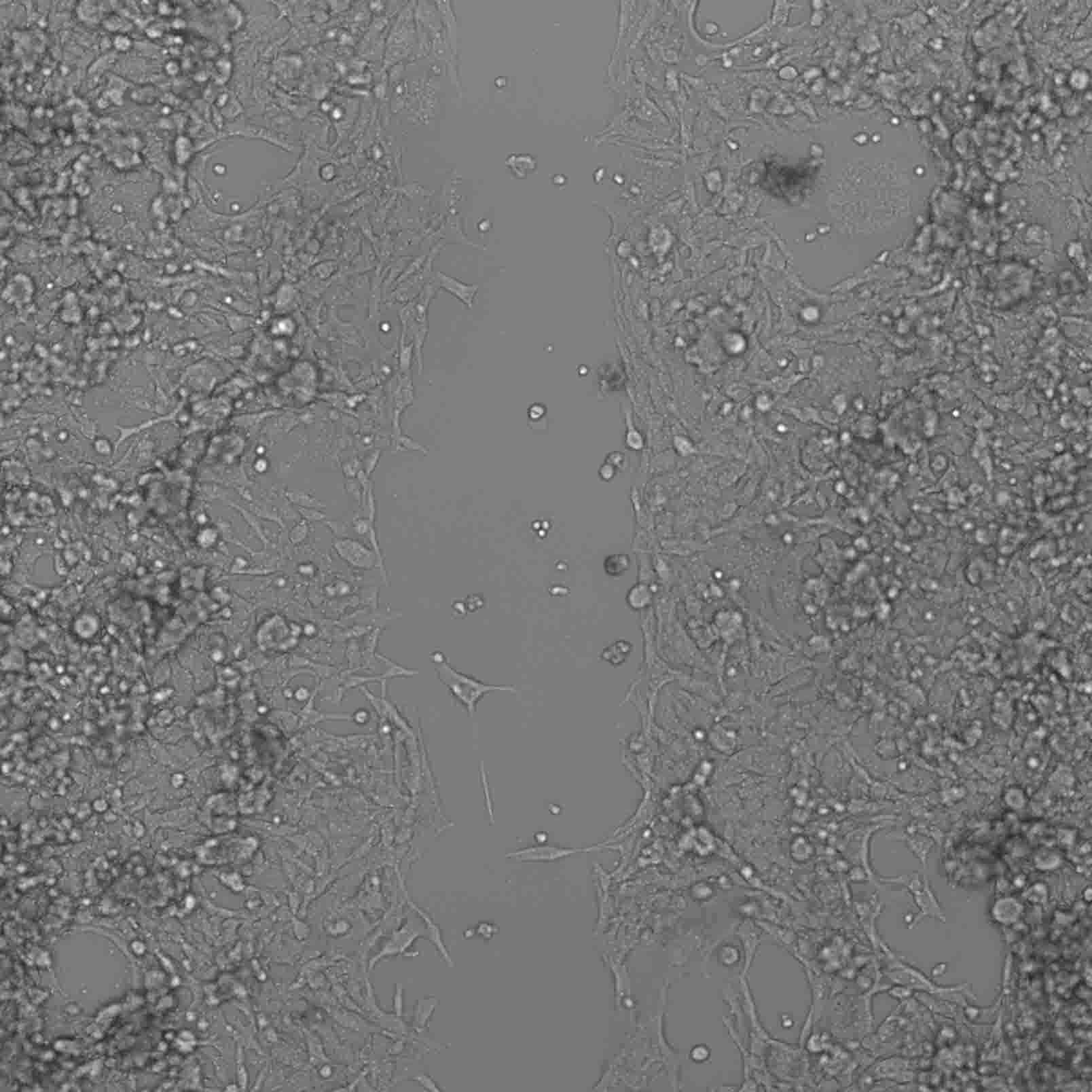

Supplement: Supplemental Information 3 [file peerj-13-20224-s003.zip › FIGURE2/FIG-2F-H/CAKi1/24/Control.jpg]

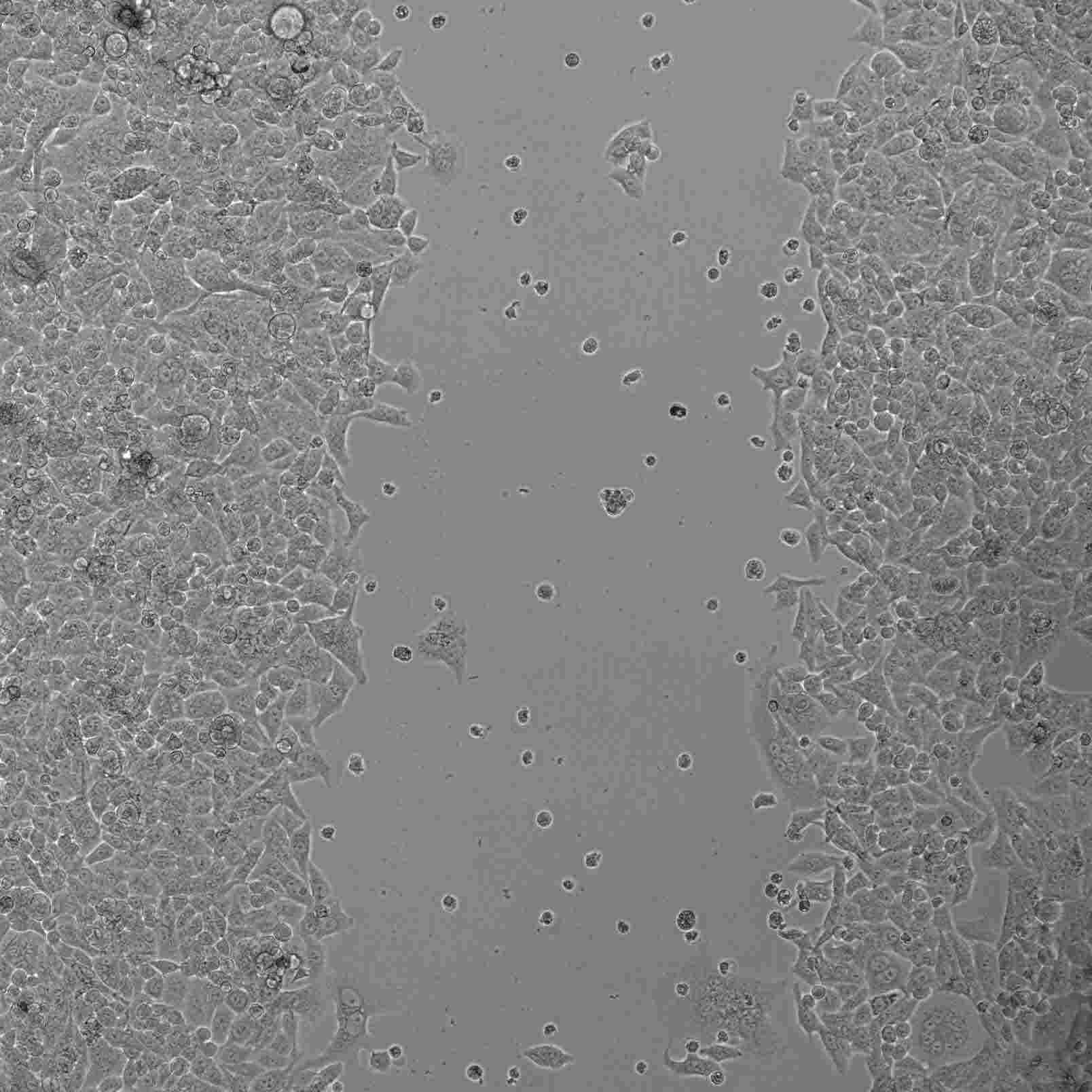

Supplement: Supplemental Information 3 [file peerj-13-20224-s003.zip › FIGURE2/FIG-2F-H/CAKi1/24/OE-NAT10.jpg]

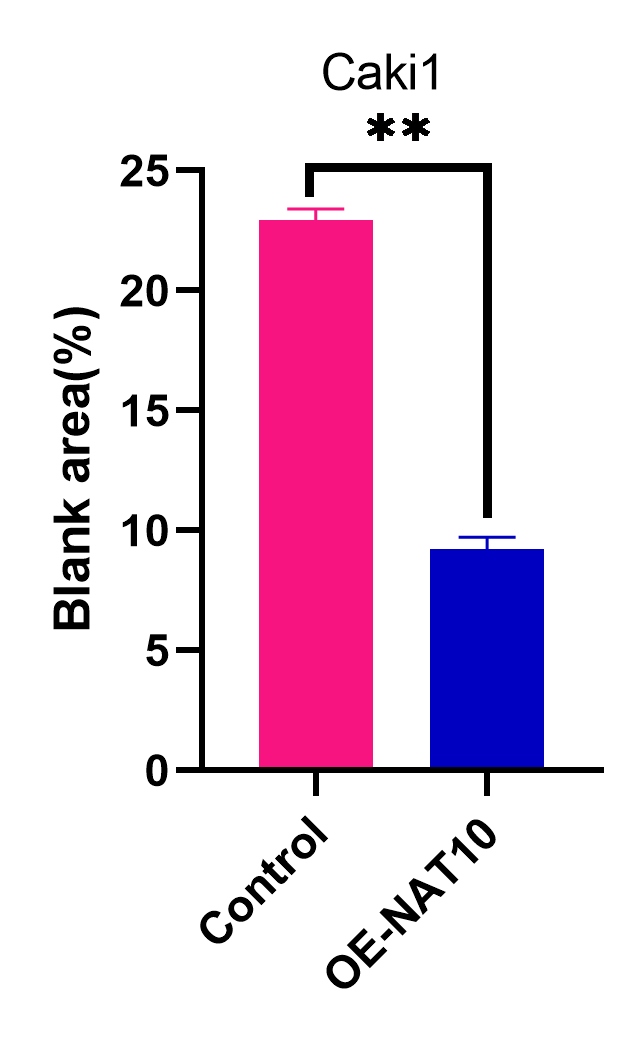

Supplement: Supplemental Information 3 [file peerj-13-20224-s003.zip › FIGURE2/FIG-2F-H/CAKi1/FIG-2H.tif]

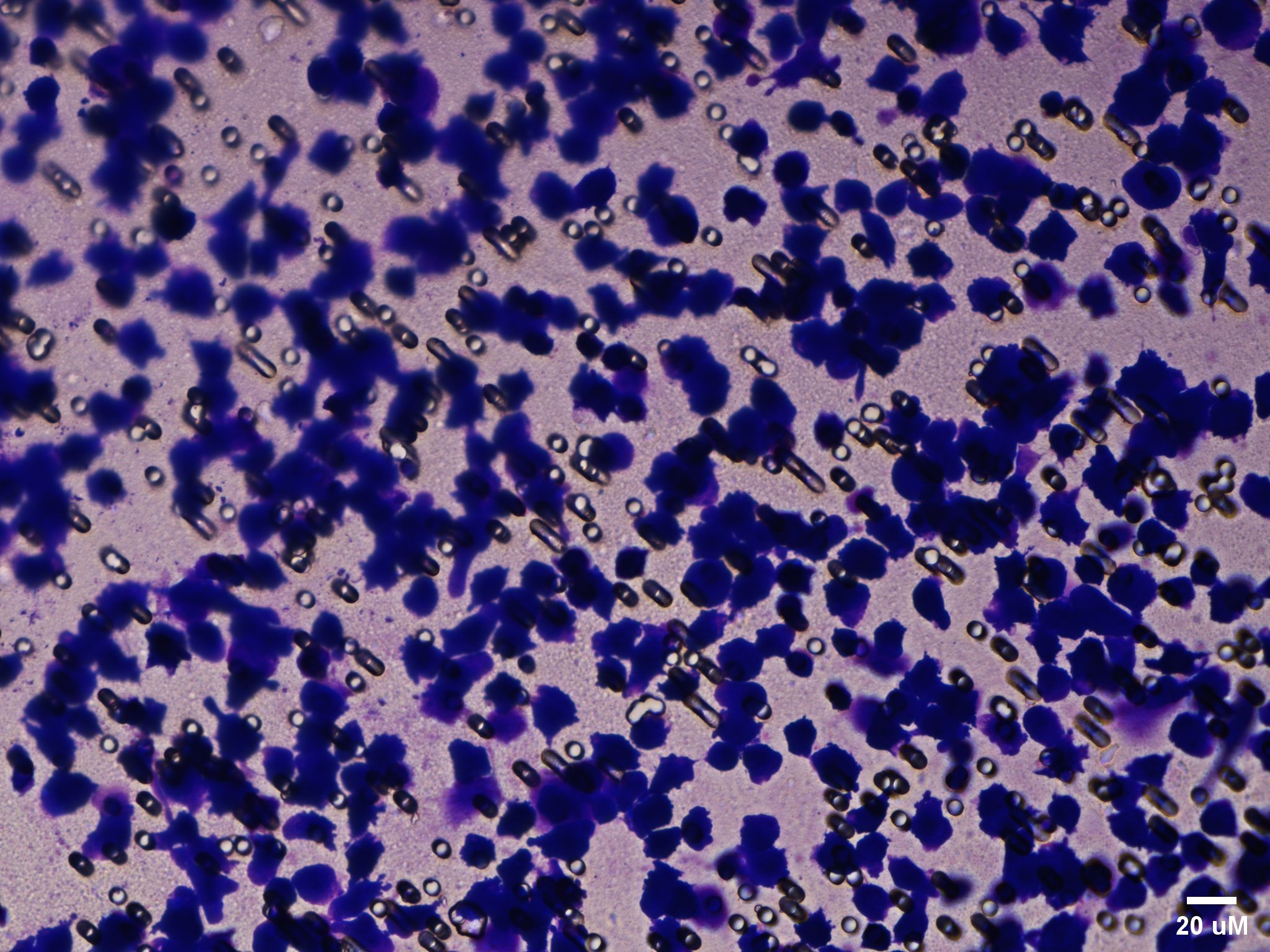

Supplement: Supplemental Information 3 [file peerj-13-20224-s003.zip › FIGURE2/FIG-2I-J/ACHN/Control.jpg]

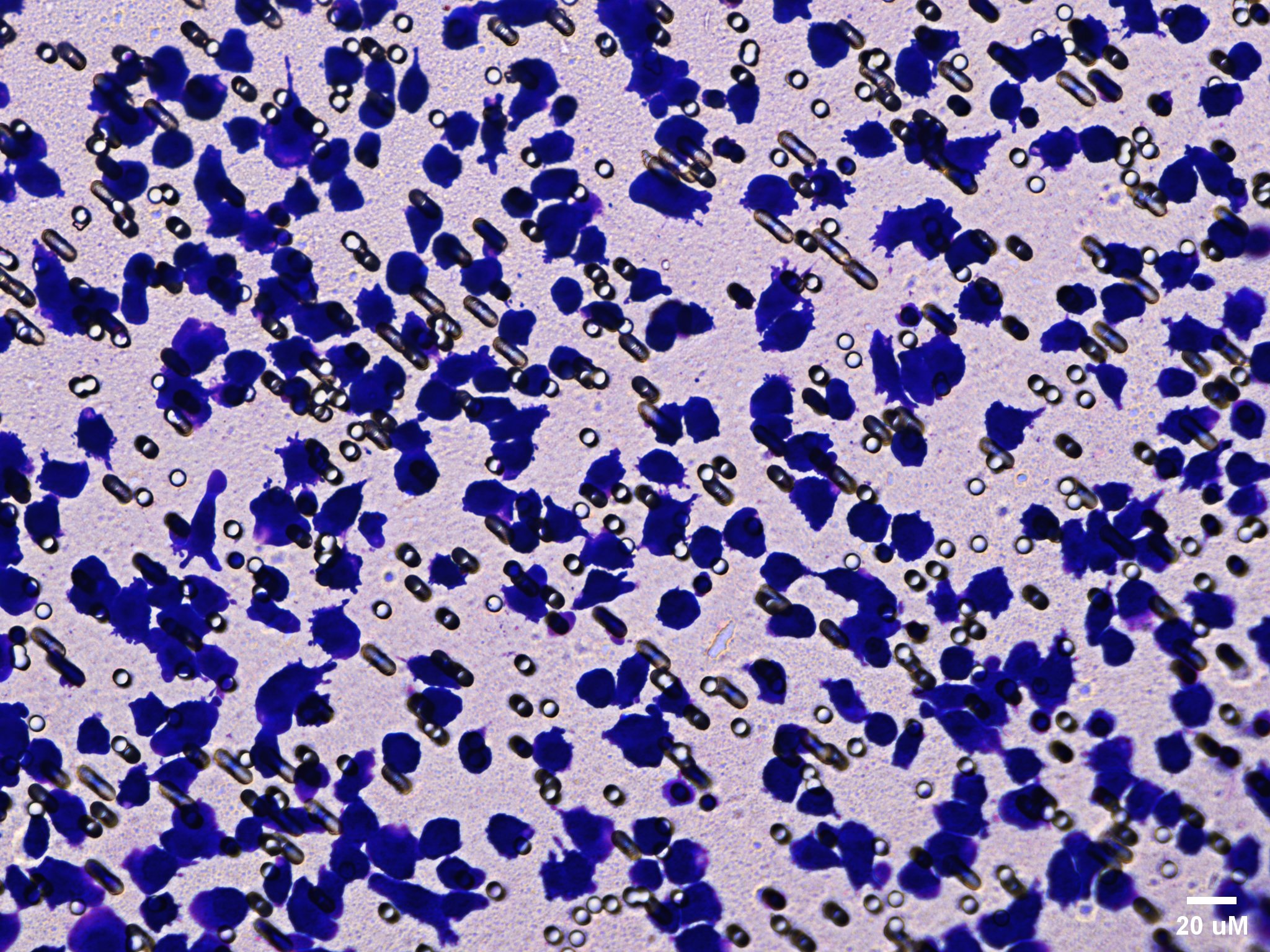

Supplement: Supplemental Information 3 [file peerj-13-20224-s003.zip › FIGURE2/FIG-2I-J/ACHN/shNAT10.jpg]

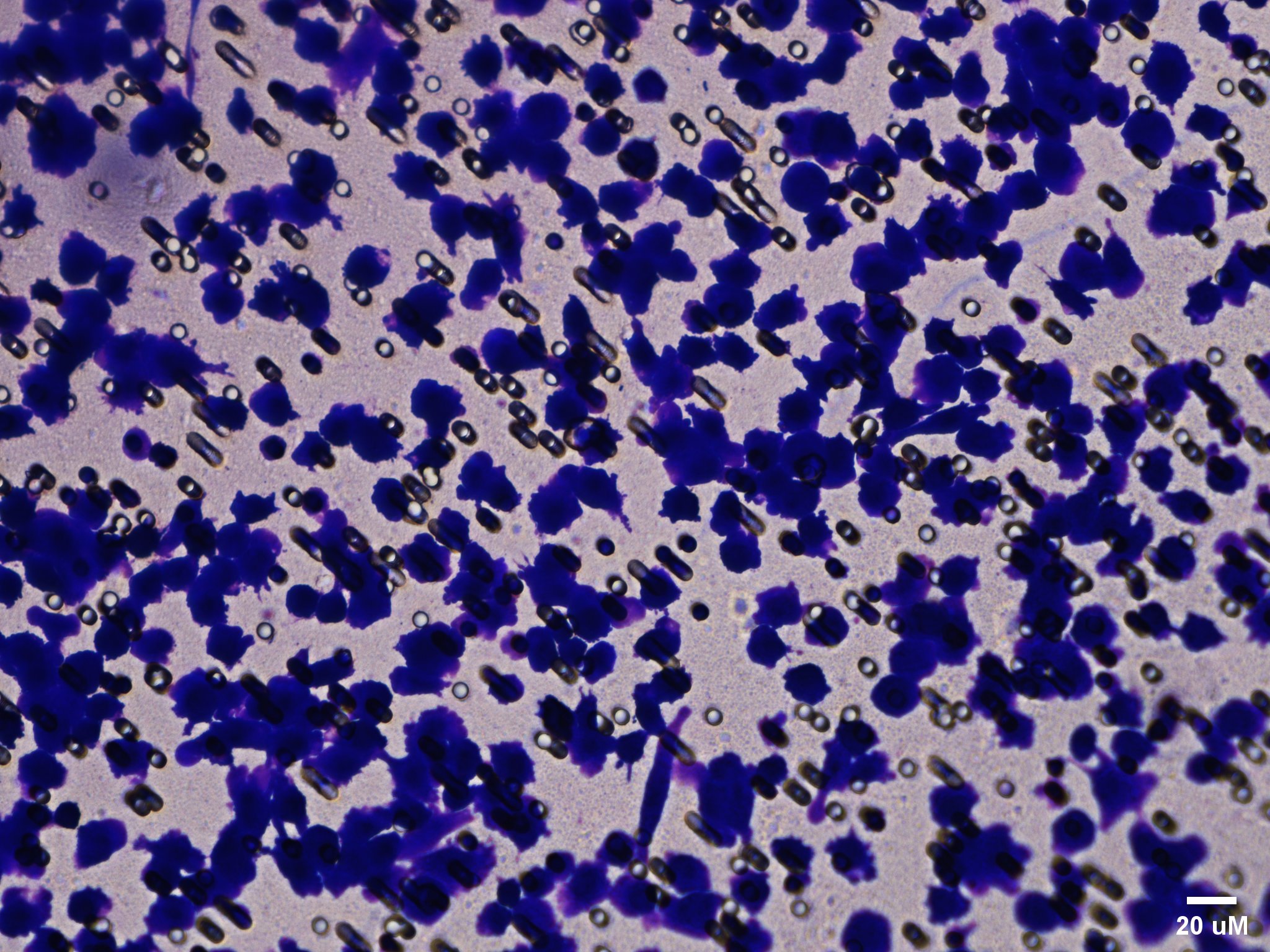

Supplement: Supplemental Information 3 [file peerj-13-20224-s003.zip › FIGURE2/FIG-2I-J/caki1/Control.jpg]

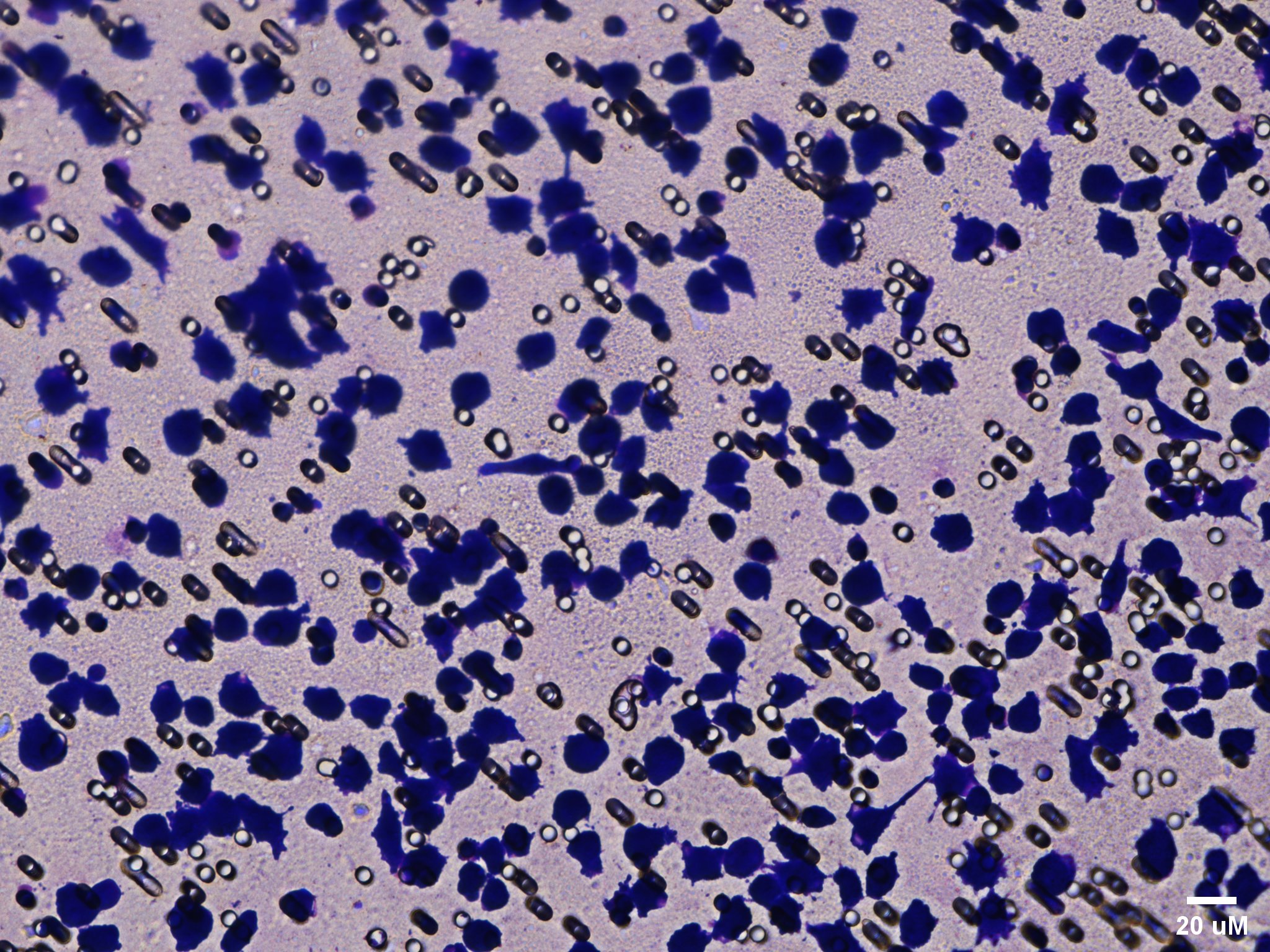

Supplement: Supplemental Information 3 [file peerj-13-20224-s003.zip › FIGURE2/FIG-2I-J/caki1/OE-NAT10.jpg]

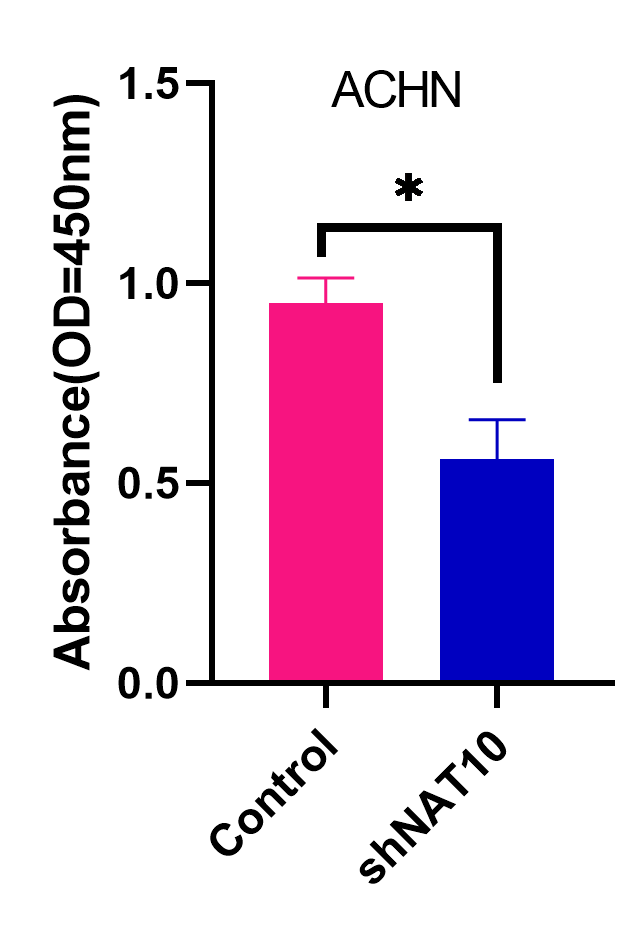

Supplement: Supplemental Information 3 [file peerj-13-20224-s003.zip › FIGURE2/FIG-2I-J/FIG-2I.tif]

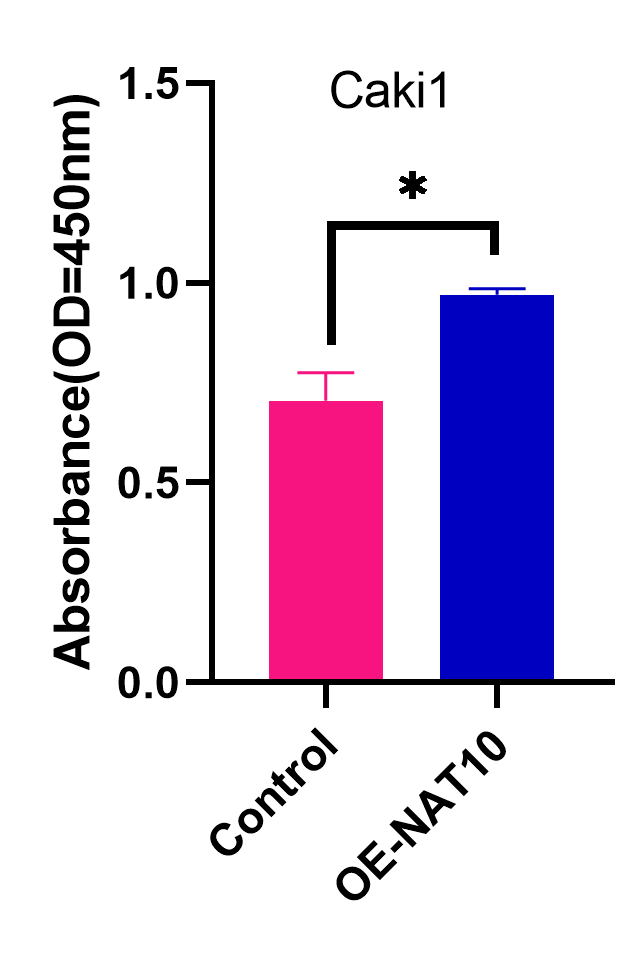

Supplement: Supplemental Information 3 [file peerj-13-20224-s003.zip › FIGURE2/FIG-2I-J/FIG-2J.tif]

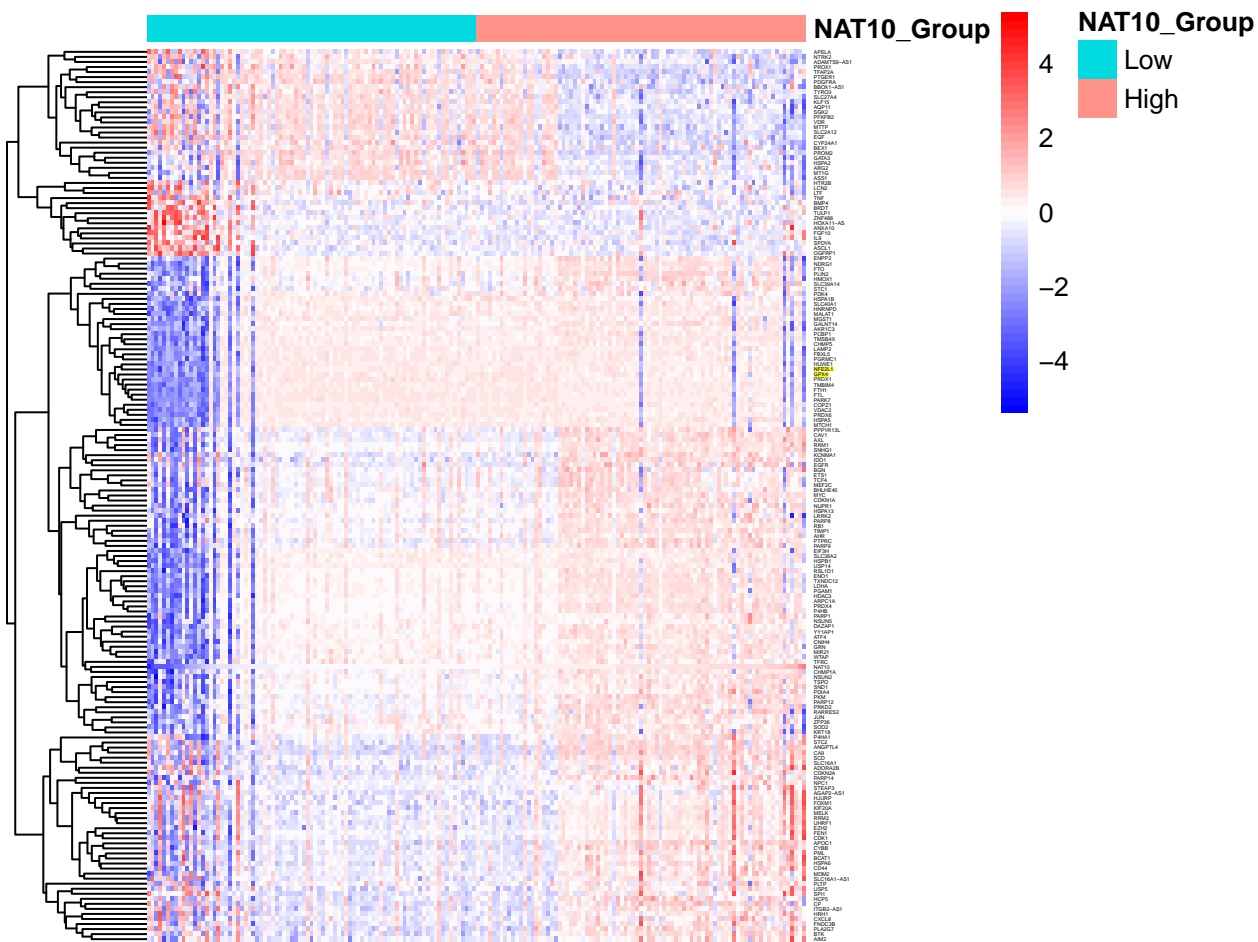

Supplement: Supplemental Information 4 [file peerj-13-20224-s004.zip › FIGURE3/Fig.3B.pdf]

# KEGG Pathway Enrichment

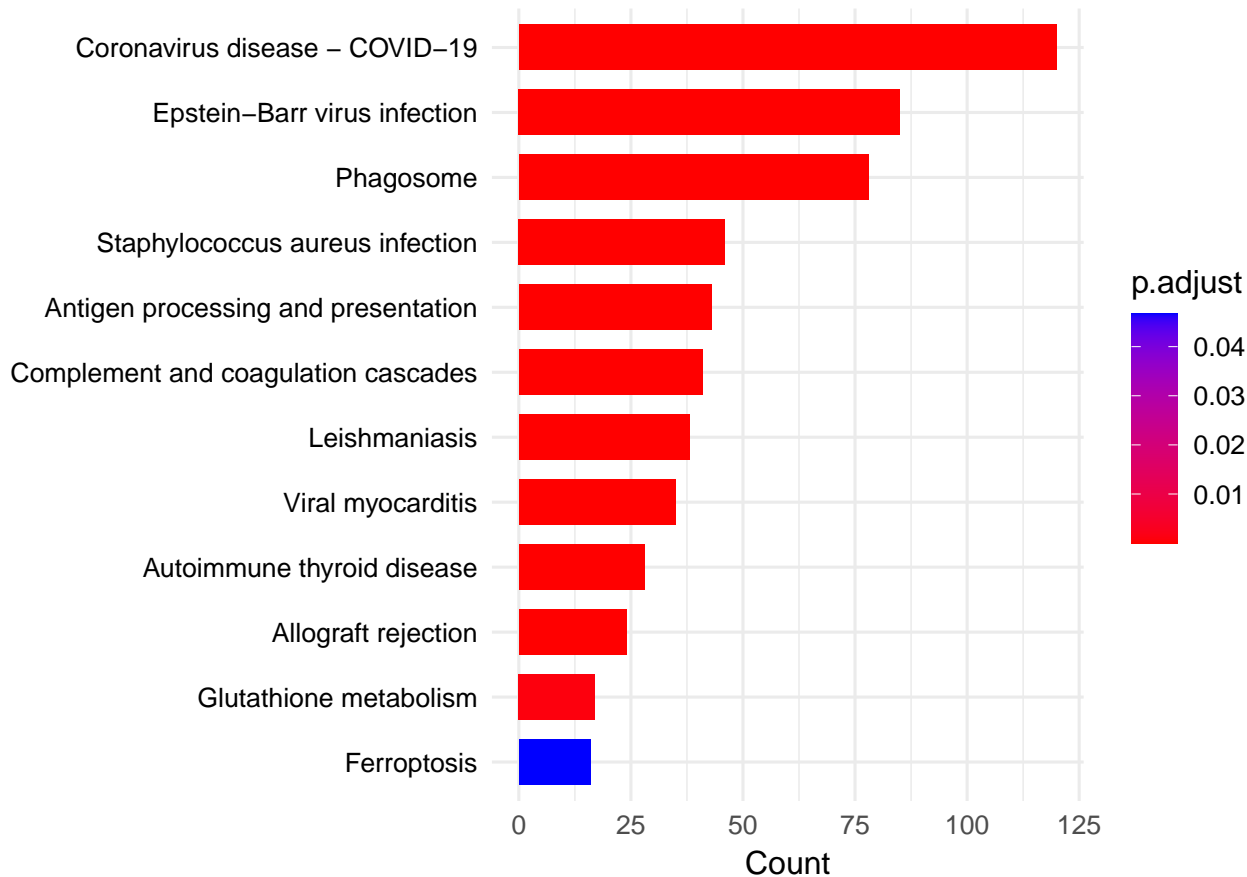

Supplement: Supplemental Information 4 [file peerj-13-20224-s004.zip › FIGURE3/Fig.3C.pdf]

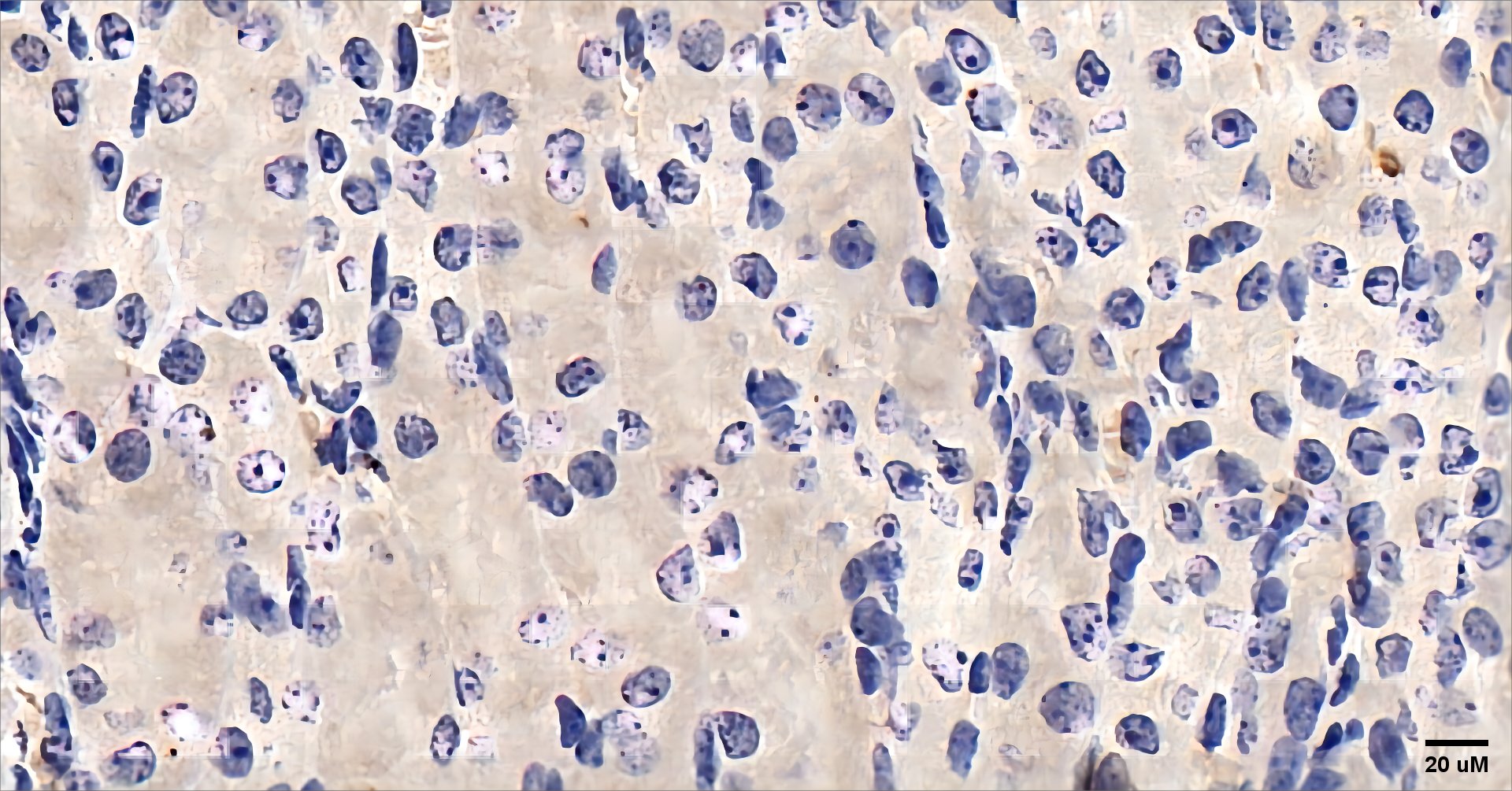

Supplement: Supplemental Information 5 [file peerj-13-20224-s005.zip › FIGURE4/FIG-4A/GPX4/组化/Normal.jpg]

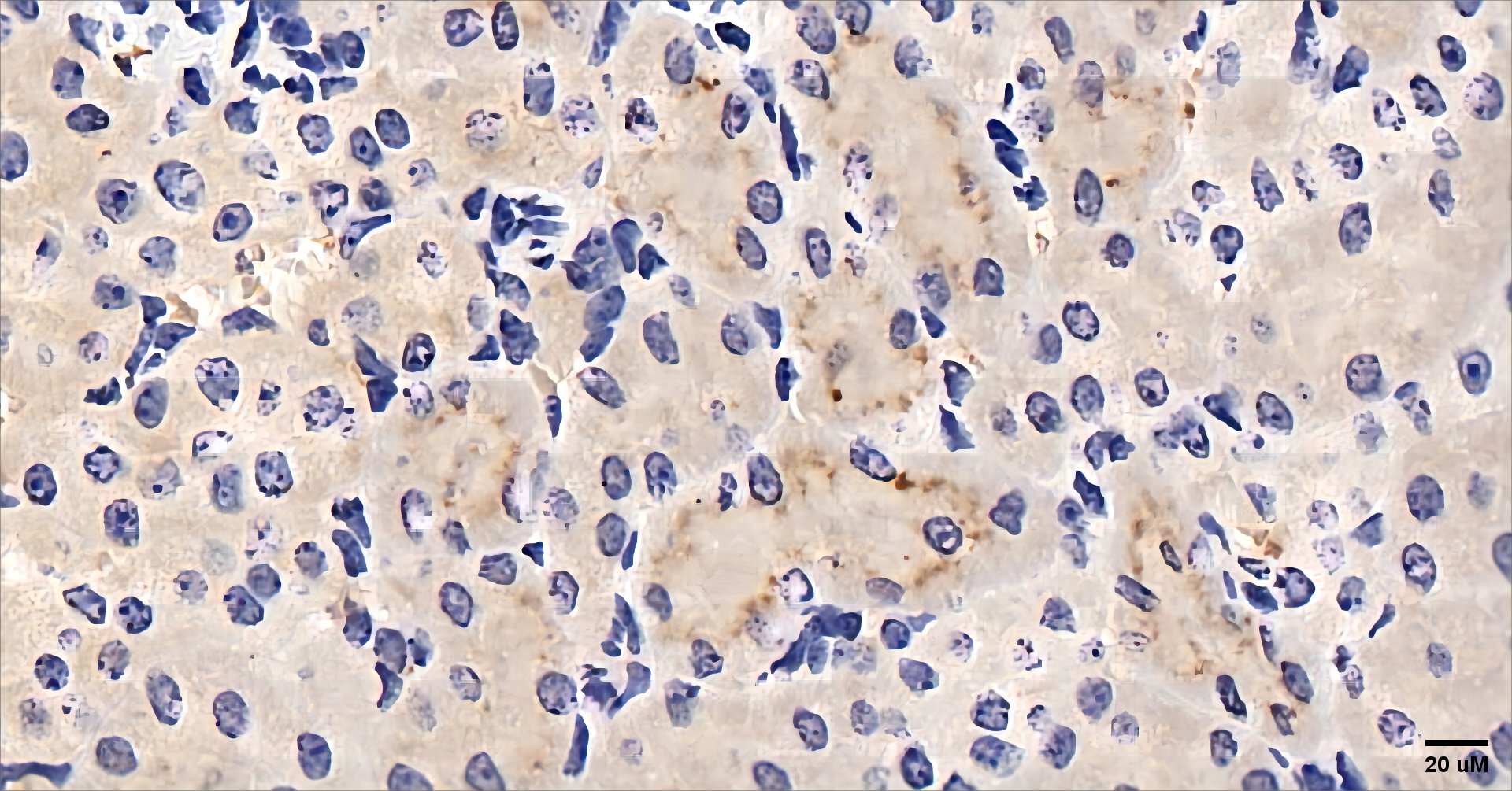

Supplement: Supplemental Information 5 [file peerj-13-20224-s005.zip › FIGURE4/FIG-4A/GPX4/组化/Tumor.jpg]

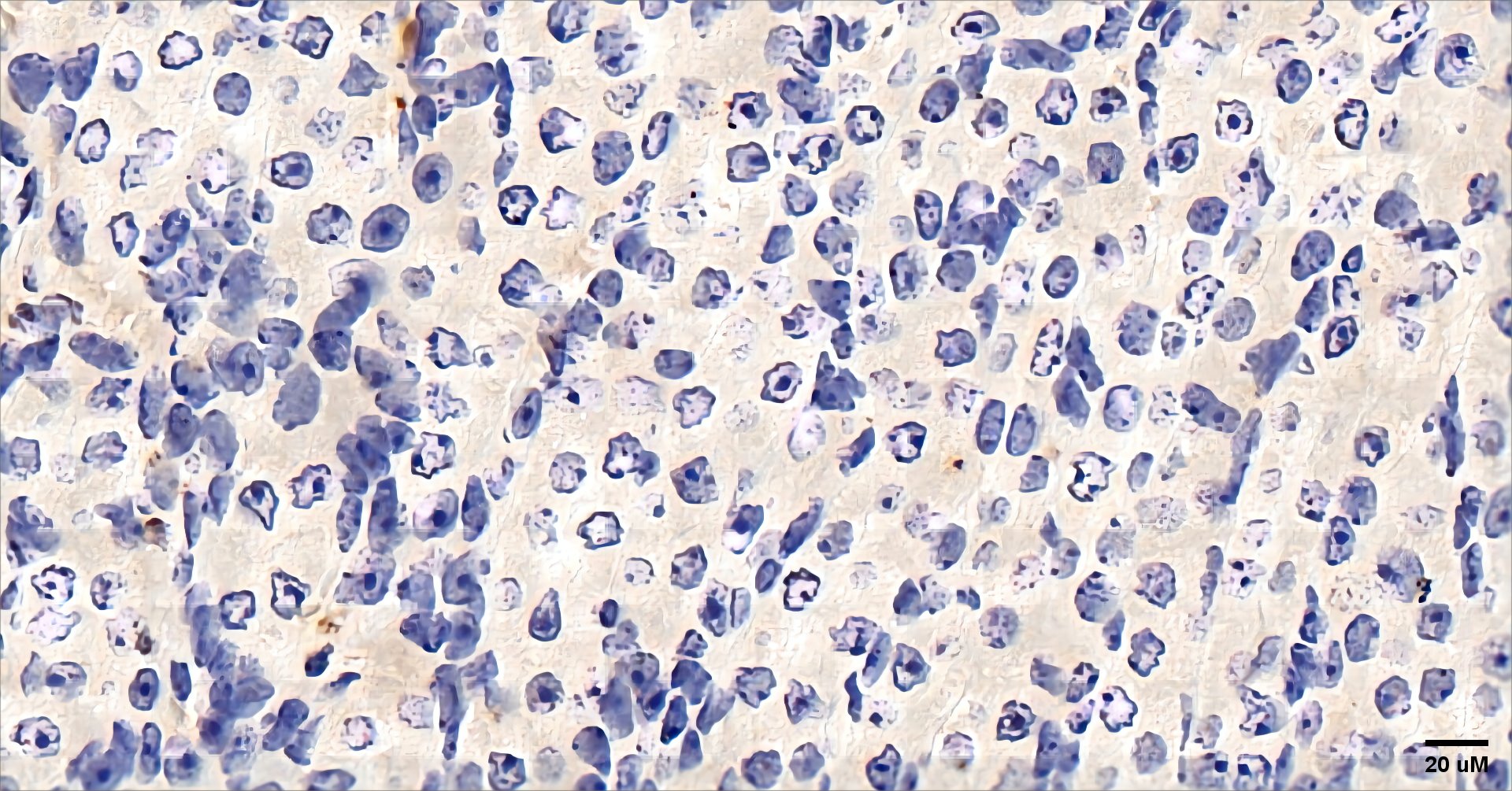

Supplement: Supplemental Information 5 [file peerj-13-20224-s005.zip › FIGURE4/FIG-4A/NFE2L1/组化/Normal.jpg]

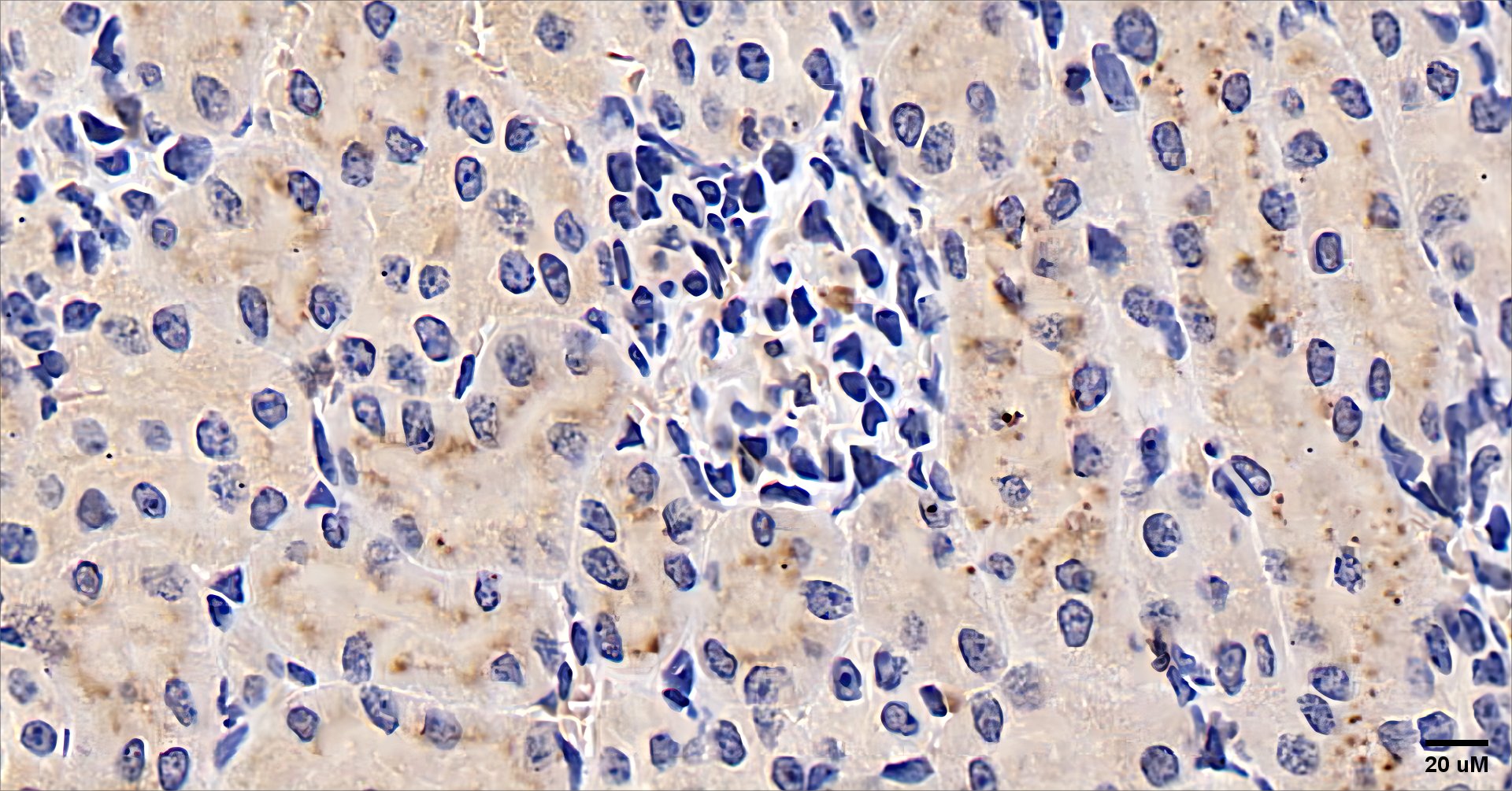

Supplement: Supplemental Information 5 [file peerj-13-20224-s005.zip › FIGURE4/FIG-4A/NFE2L1/组化/Tumor.jpg]

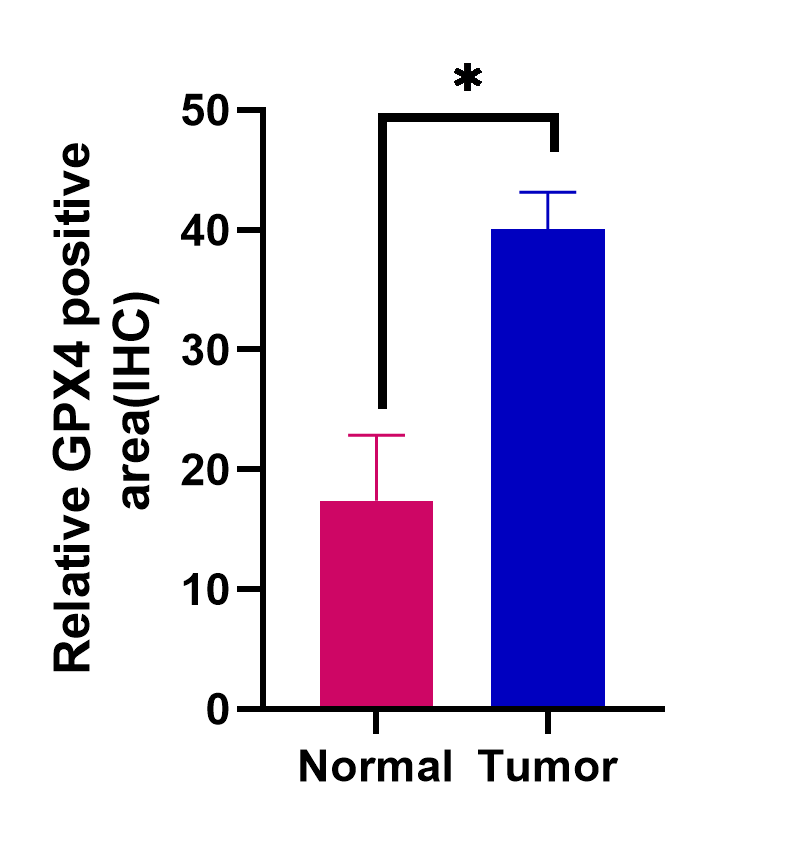

Supplement: Supplemental Information 5 [file peerj-13-20224-s005.zip › FIGURE4/FIG-4B/FIG-4B.tif]

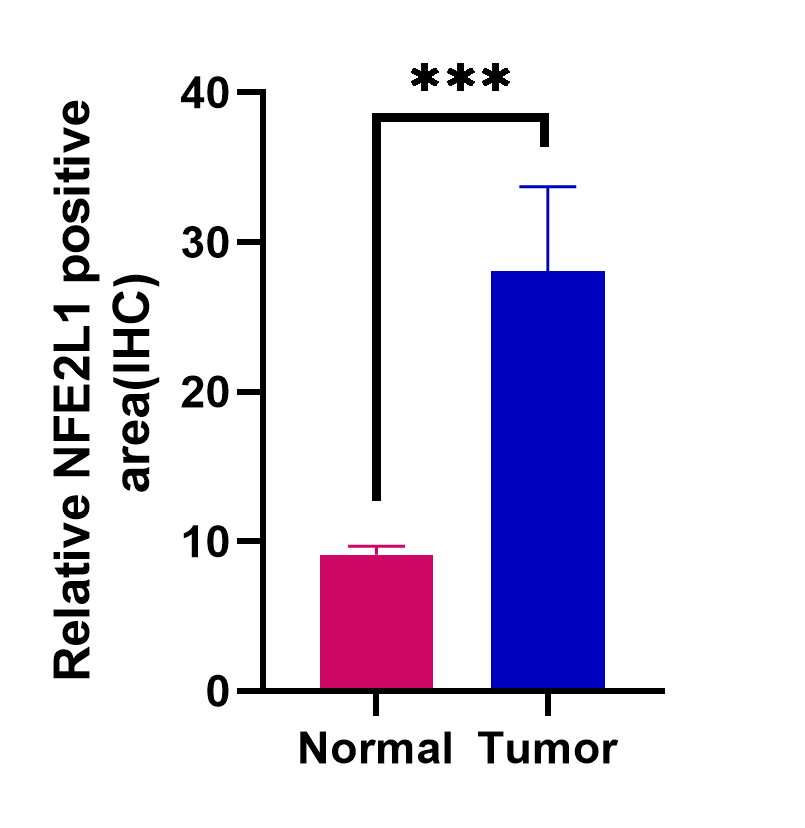

Supplement: Supplemental Information 5 [file peerj-13-20224-s005.zip › FIGURE4/FIG-4C/FIG-4C.tif]

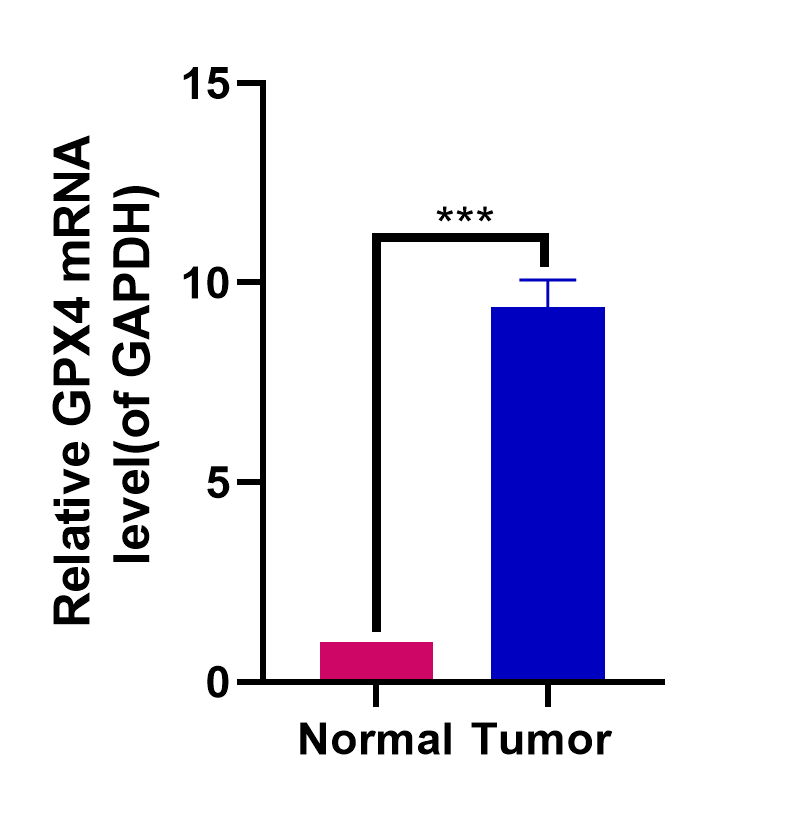

Supplement: Supplemental Information 5 [file peerj-13-20224-s005.zip › FIGURE4/FIG-4D/FIG-4D.tif]

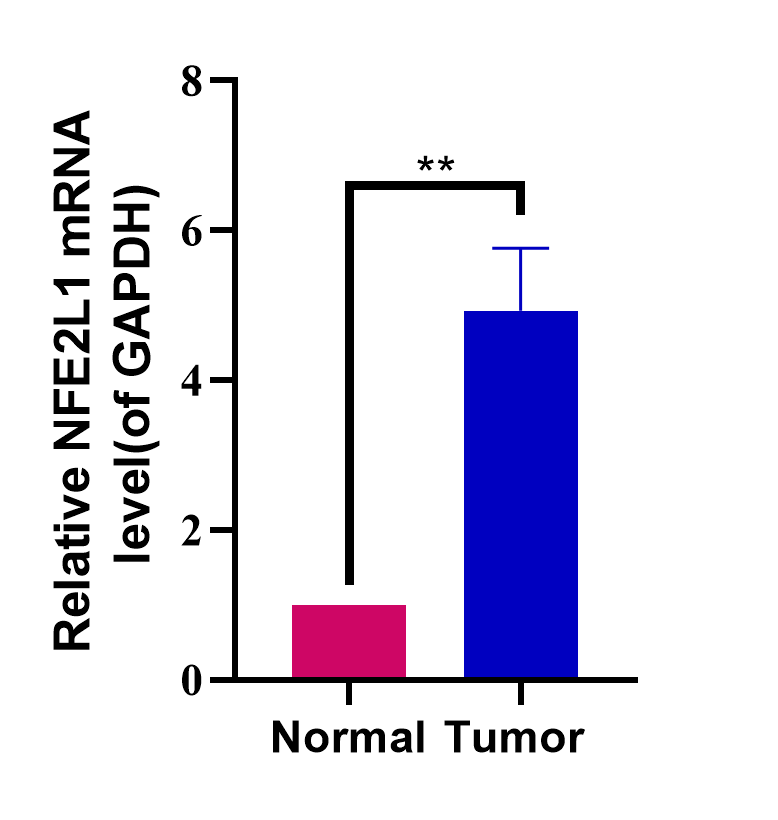

Supplement: Supplemental Information 5 [file peerj-13-20224-s005.zip › FIGURE4/FIG-4E/FIG-4E.tif]

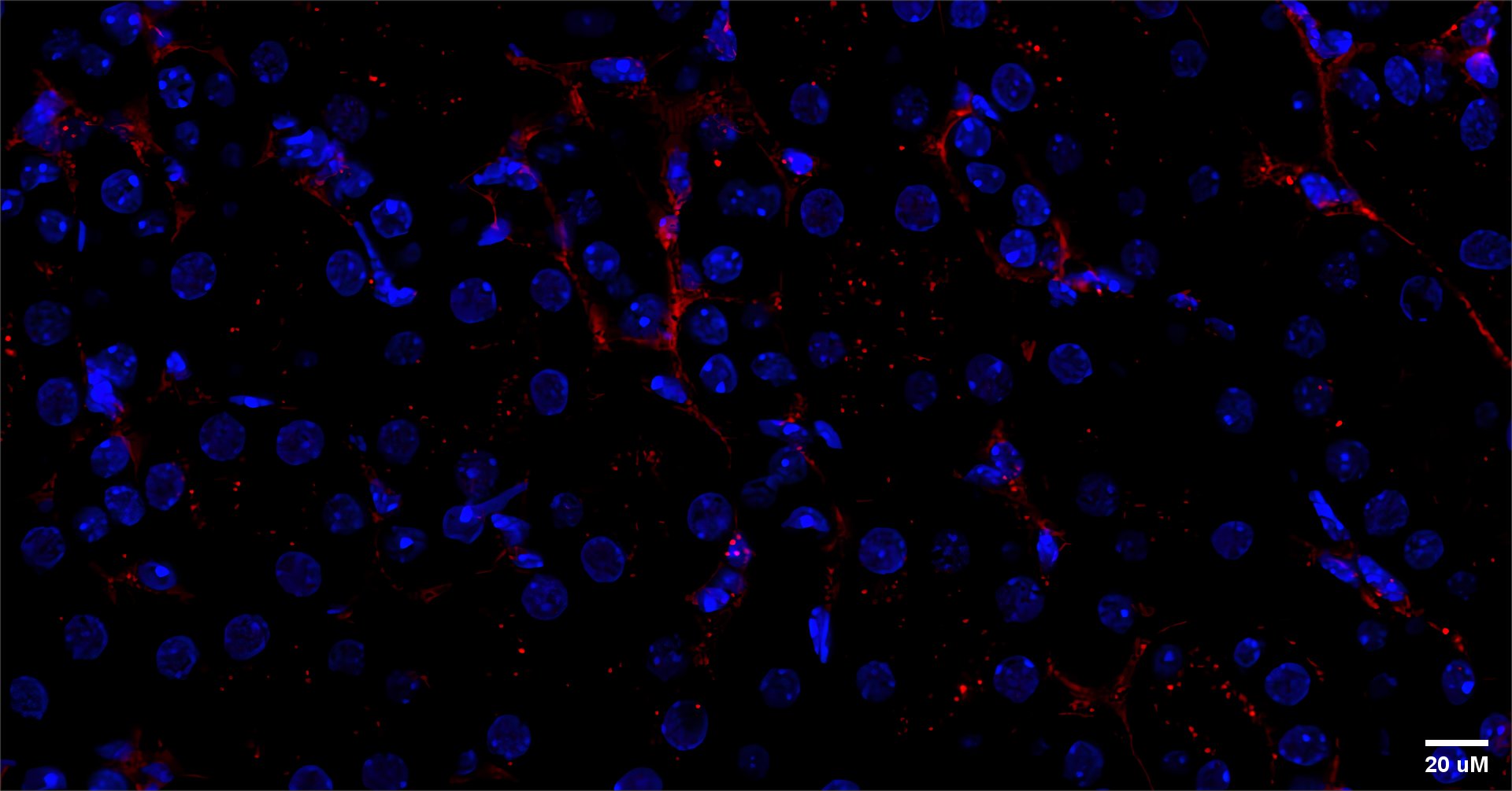

Supplement: Supplemental Information 5 [file peerj-13-20224-s005.zip › FIGURE4/FIG-4F/CHAC1/Normal.jpg]

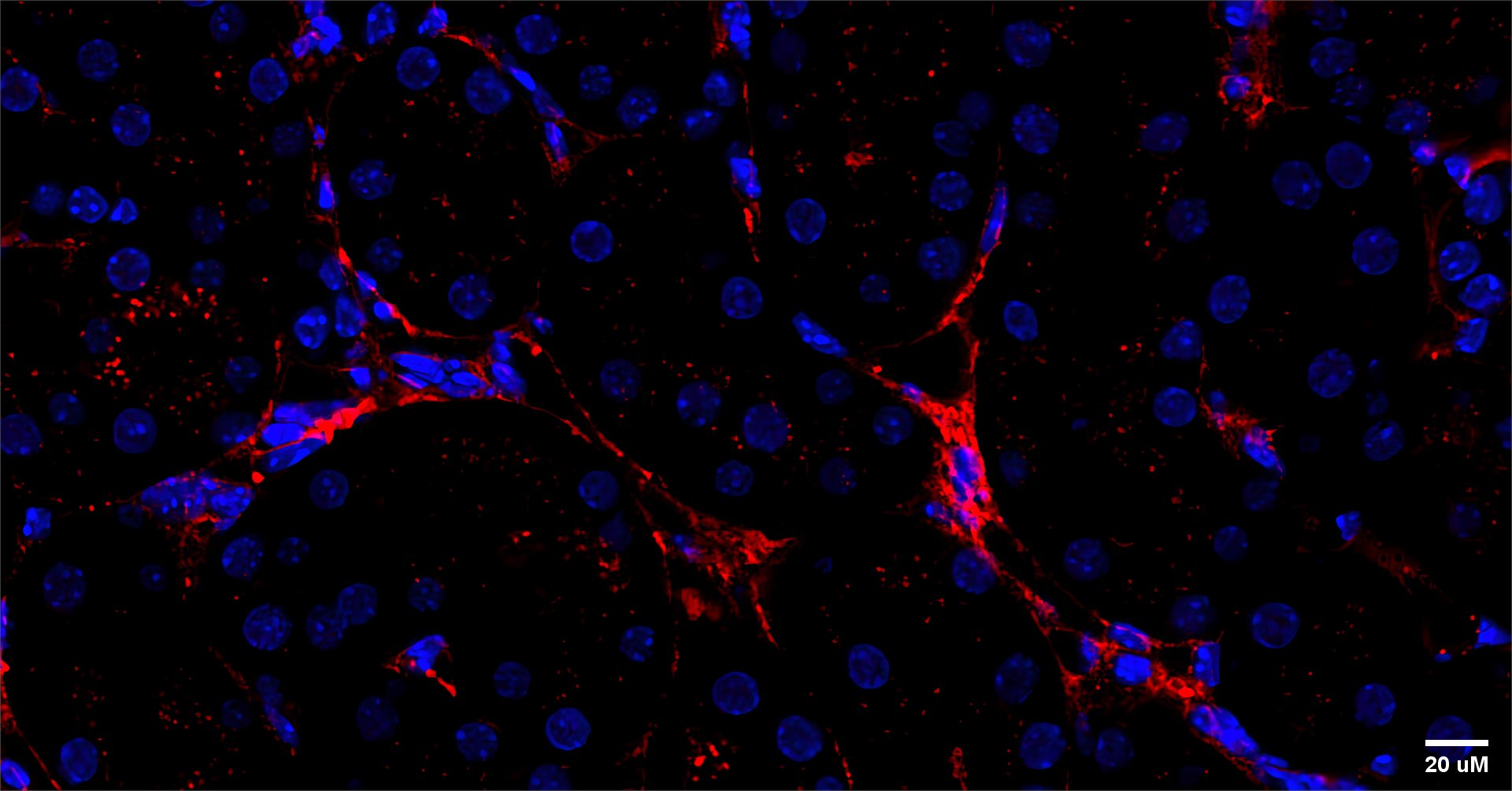

Supplement: Supplemental Information 5 [file peerj-13-20224-s005.zip › FIGURE4/FIG-4F/CHAC1/Tumor.jpg]

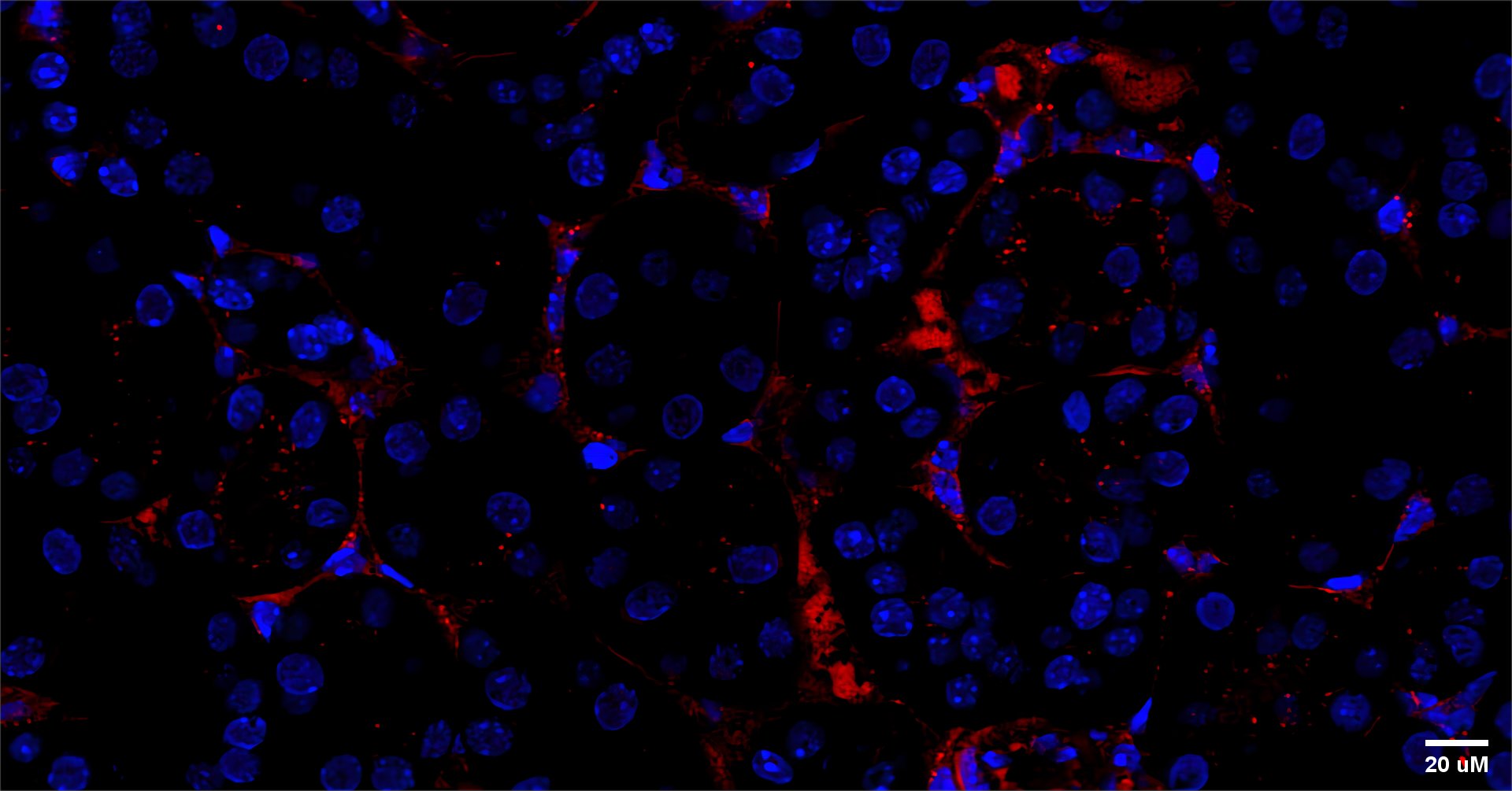

Supplement: Supplemental Information 5 [file peerj-13-20224-s005.zip › FIGURE4/FIG-4F/PTGS2/Normal.jpg]

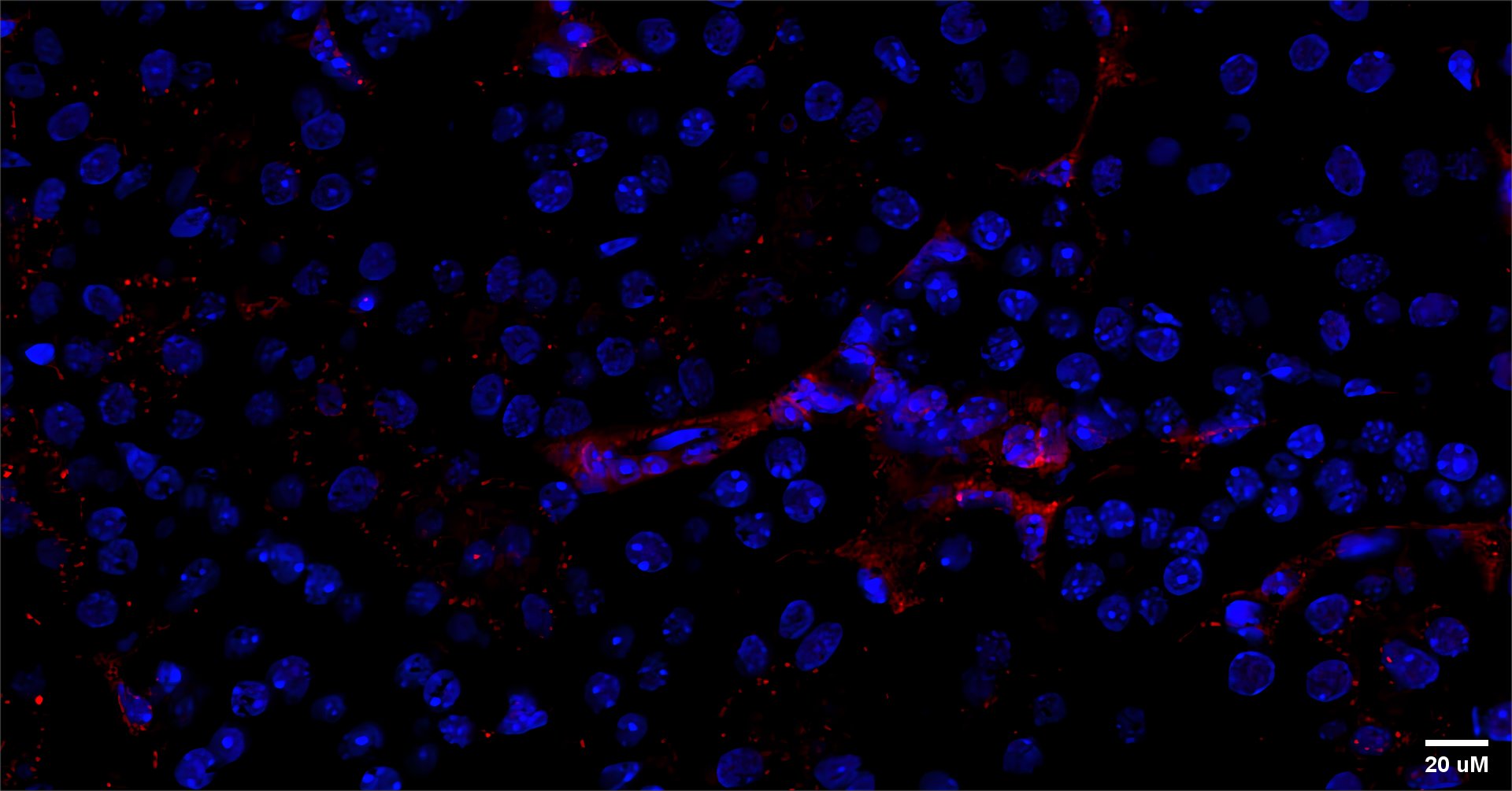

Supplement: Supplemental Information 5 [file peerj-13-20224-s005.zip › FIGURE4/FIG-4F/PTGS2/Tumor.jpg]

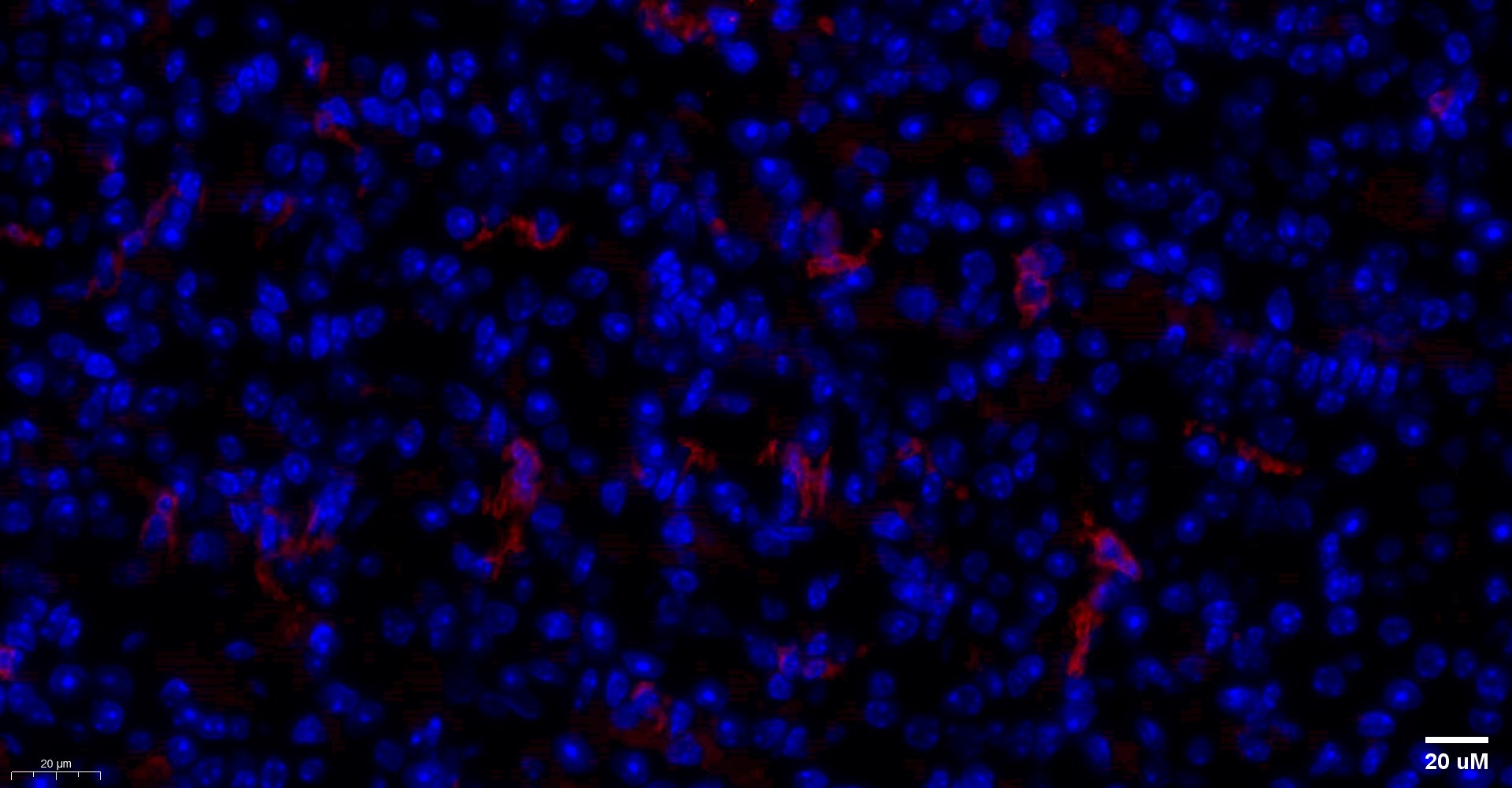

Supplement: Supplemental Information 5 [file peerj-13-20224-s005.zip › FIGURE4/FIG-4F/SLC7A11/Normal.jpg]

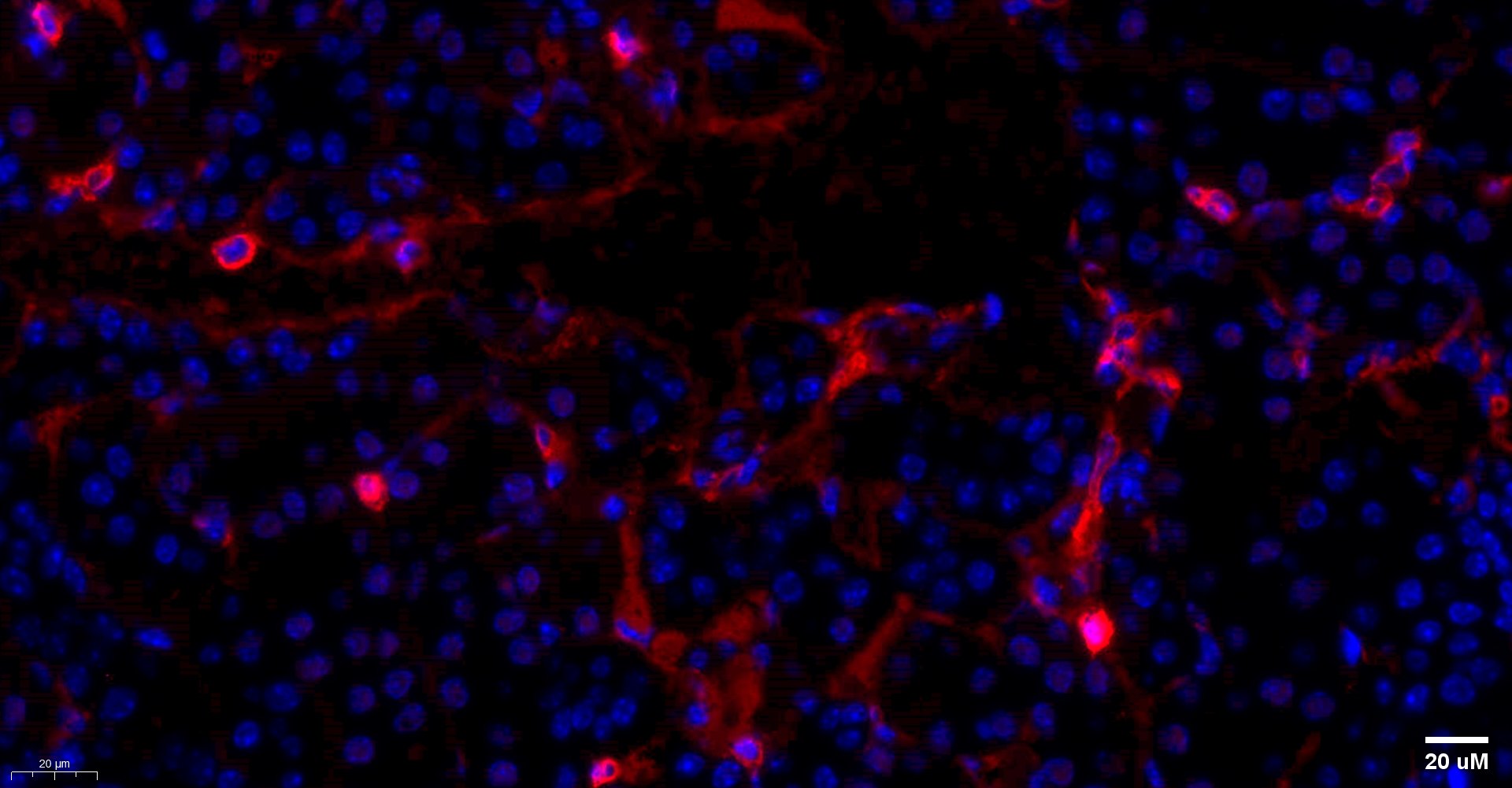

Supplement: Supplemental Information 5 [file peerj-13-20224-s005.zip › FIGURE4/FIG-4F/SLC7A11/Tumor.jpg]

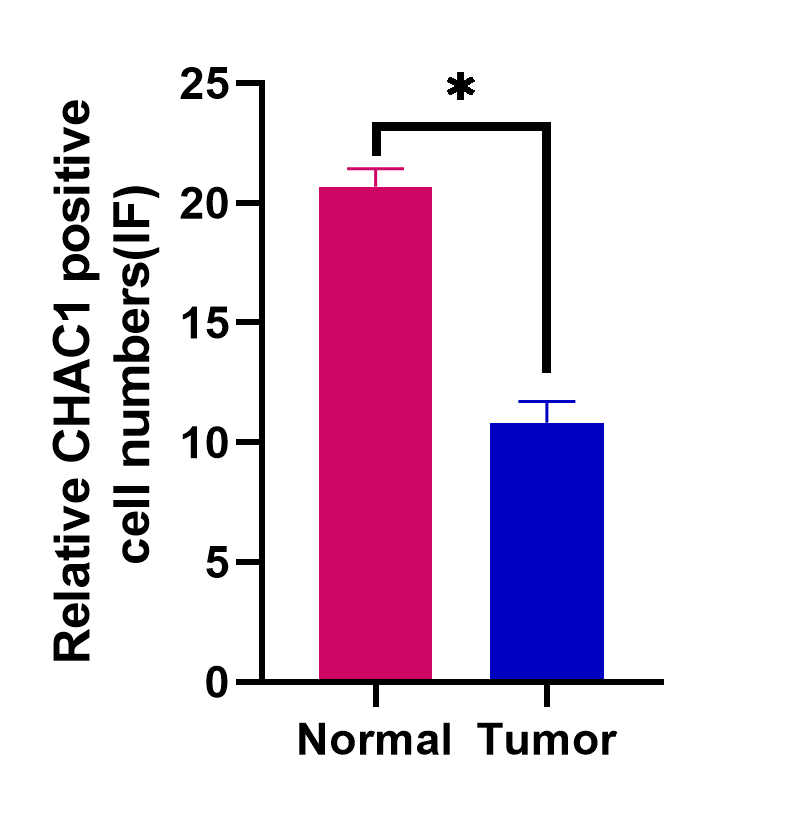

Supplement: Supplemental Information 5 [file peerj-13-20224-s005.zip › FIGURE4/FIG-4G/FIG-4G.tif]

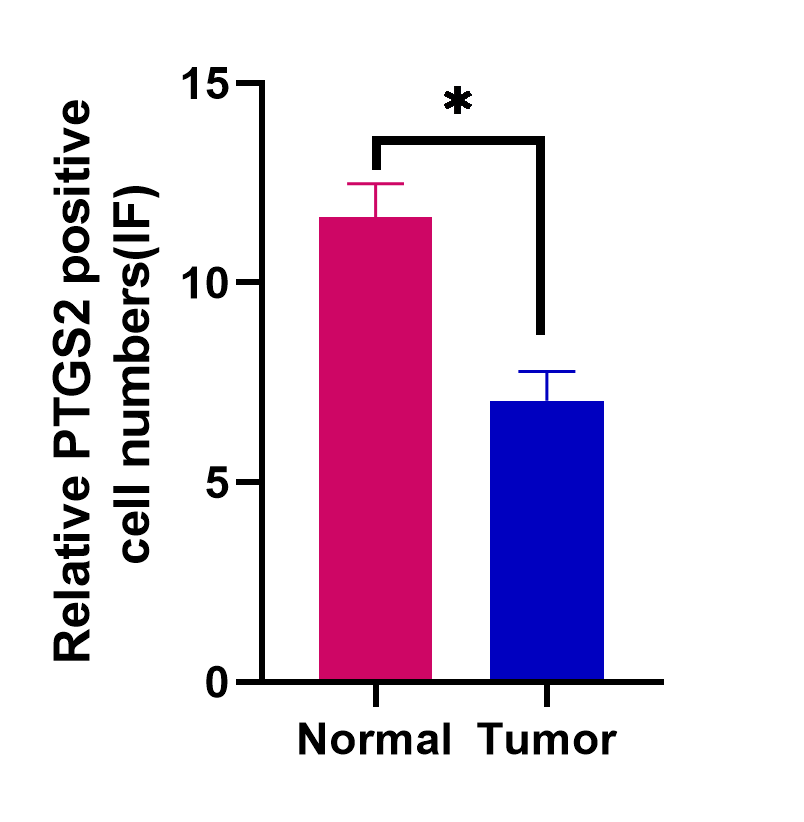

Supplement: Supplemental Information 5 [file peerj-13-20224-s005.zip › FIGURE4/FIG-4H/FIG-4H.tif]

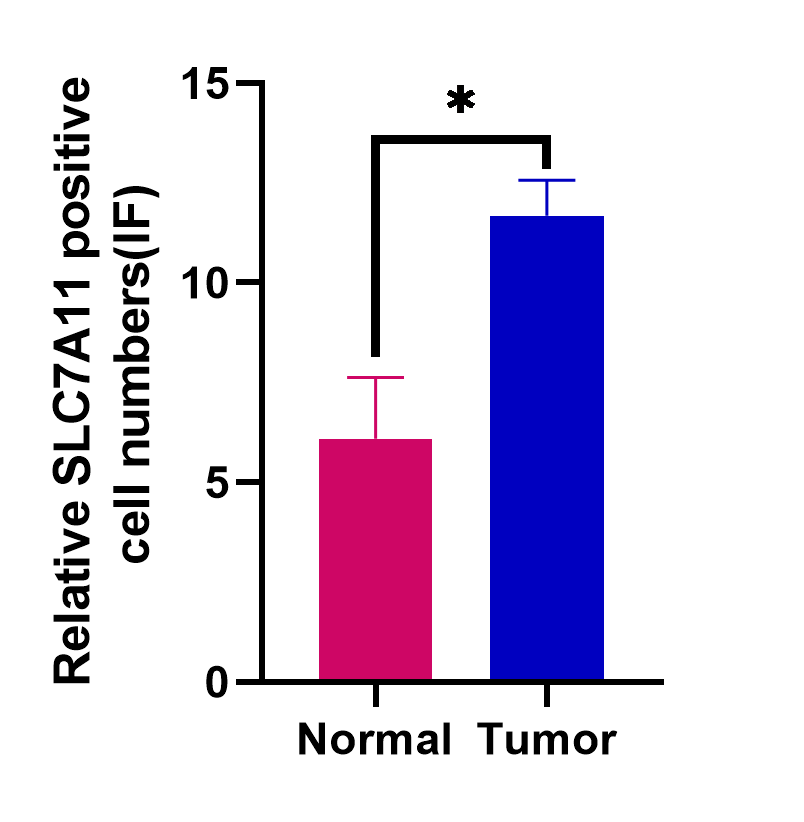

Supplement: Supplemental Information 5 [file peerj-13-20224-s005.zip › FIGURE4/FIG-4I/FIG-4I.tif]

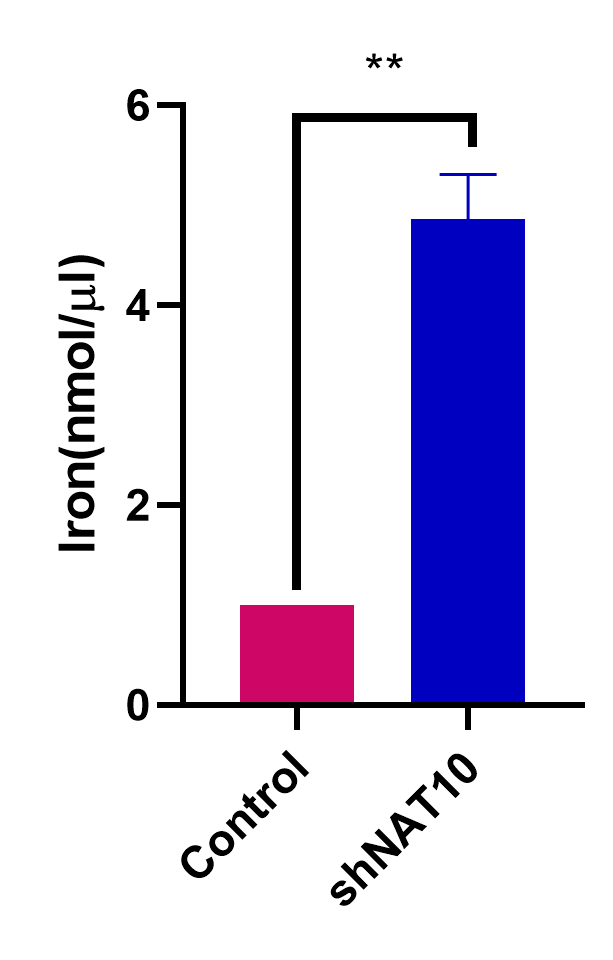

Supplement: Supplemental Information 5 [file peerj-13-20224-s005.zip › FIGURE4/FIG-4J/FIG-4J.tif]

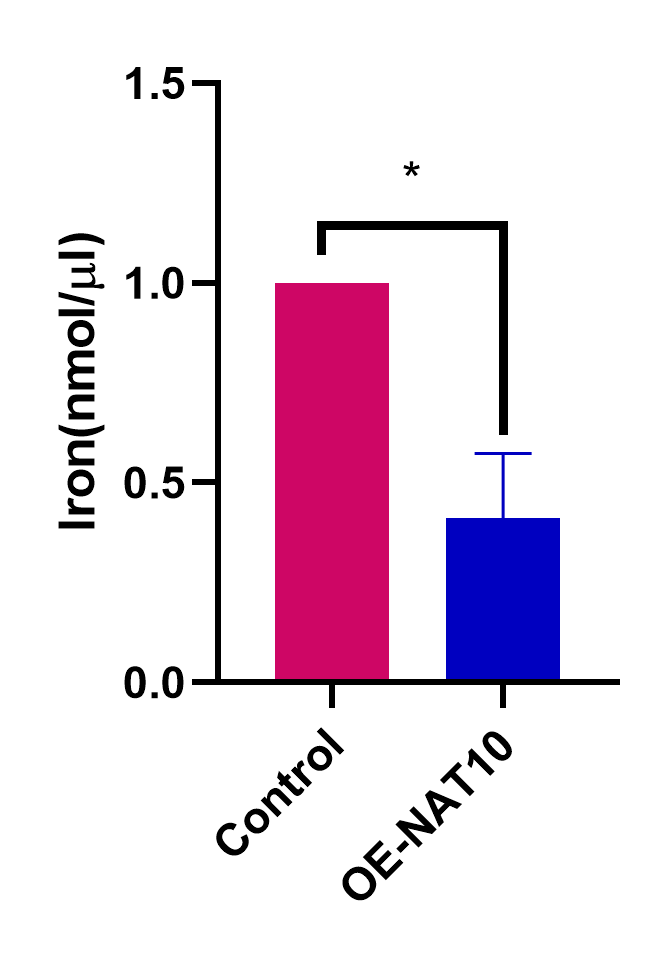

Supplement: Supplemental Information 5 [file peerj-13-20224-s005.zip › FIGURE4/FIG-4K/FIG-4K.tif]

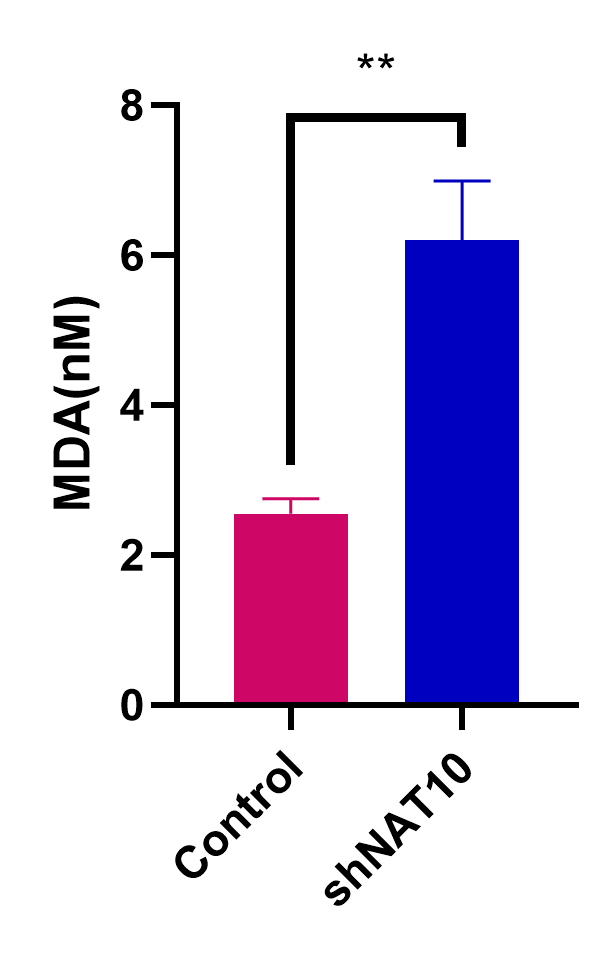

Supplement: Supplemental Information 5 [file peerj-13-20224-s005.zip › FIGURE4/FIG-4L/FIG-4L.tif]

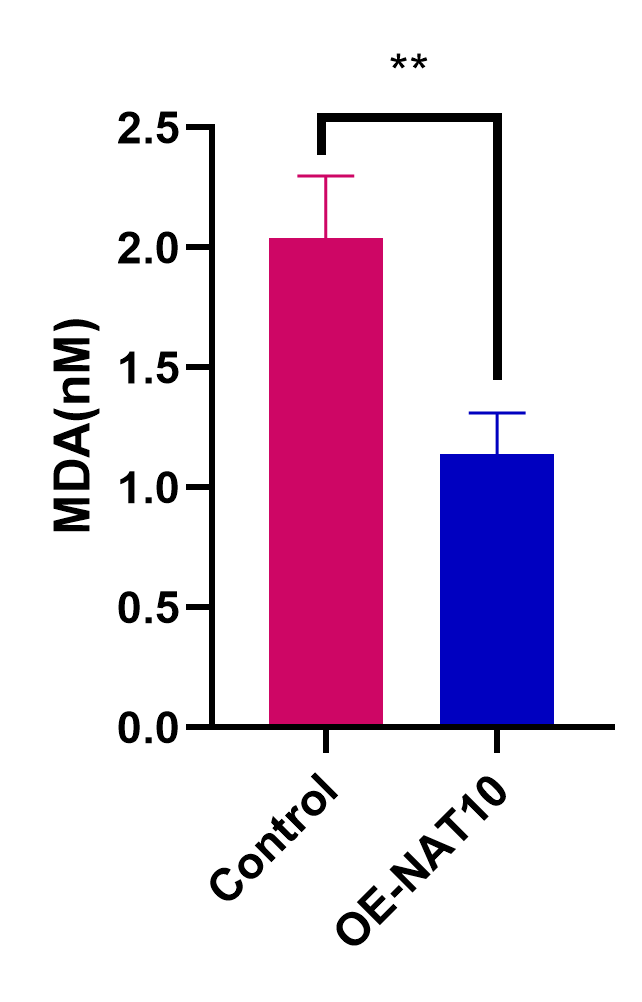

Supplement: Supplemental Information 5 [file peerj-13-20224-s005.zip › FIGURE4/FIG-4M/FIG-4M.tif]

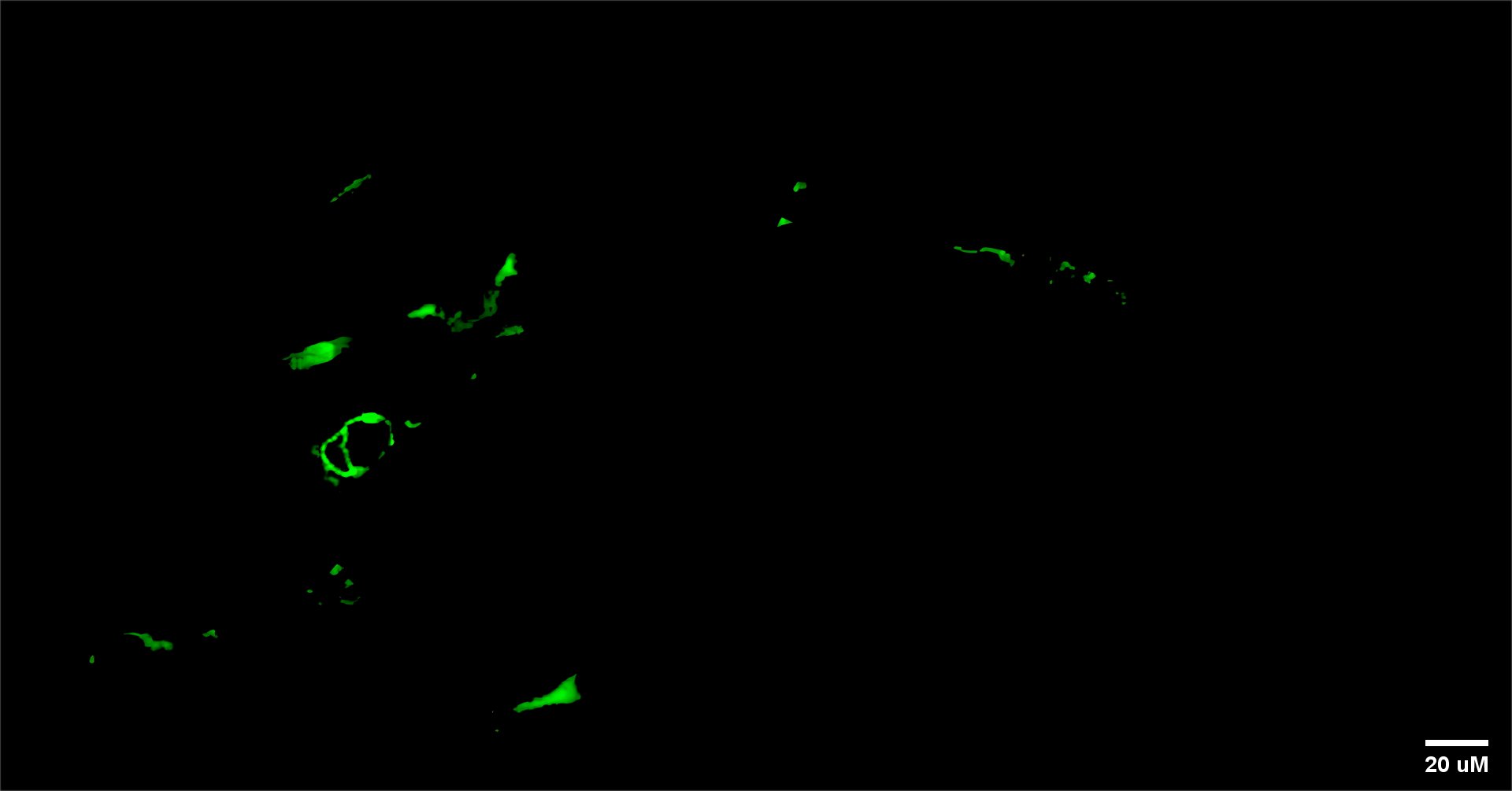

Supplement: Supplemental Information 5 [file peerj-13-20224-s005.zip › FIGURE4/FIG-4N-O/FIG-4N/Control.jpg]

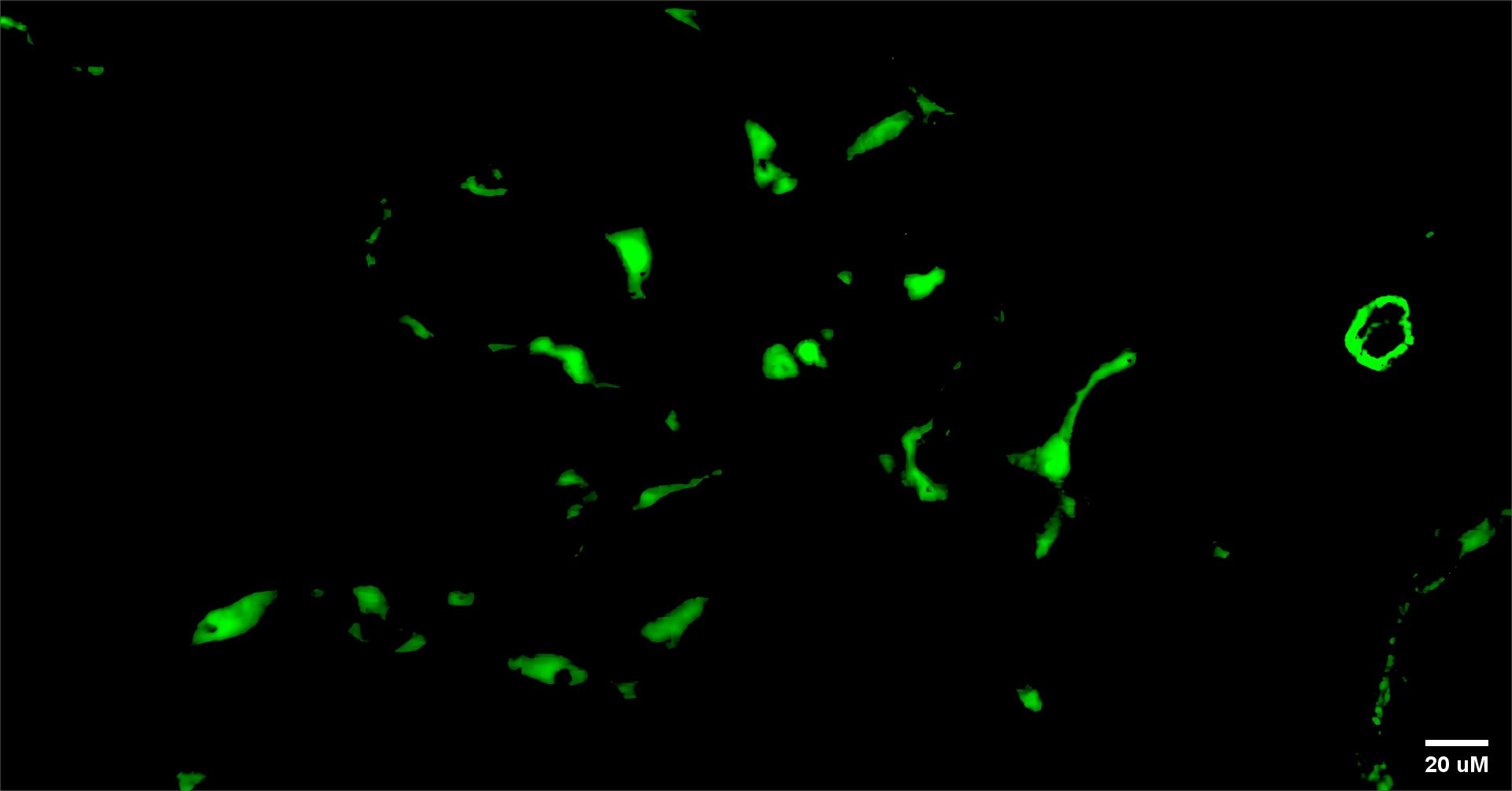

Supplement: Supplemental Information 5 [file peerj-13-20224-s005.zip › FIGURE4/FIG-4N-O/FIG-4N/shNAT10.jpg]

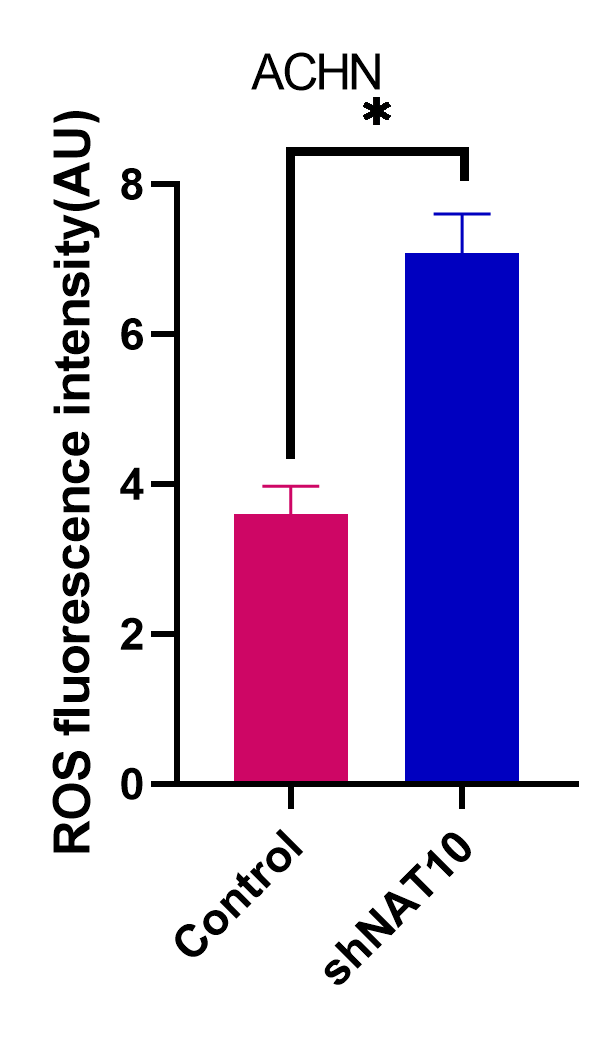

Supplement: Supplemental Information 5 [file peerj-13-20224-s005.zip › FIGURE4/FIG-4N-O/FIG-4O.tif]

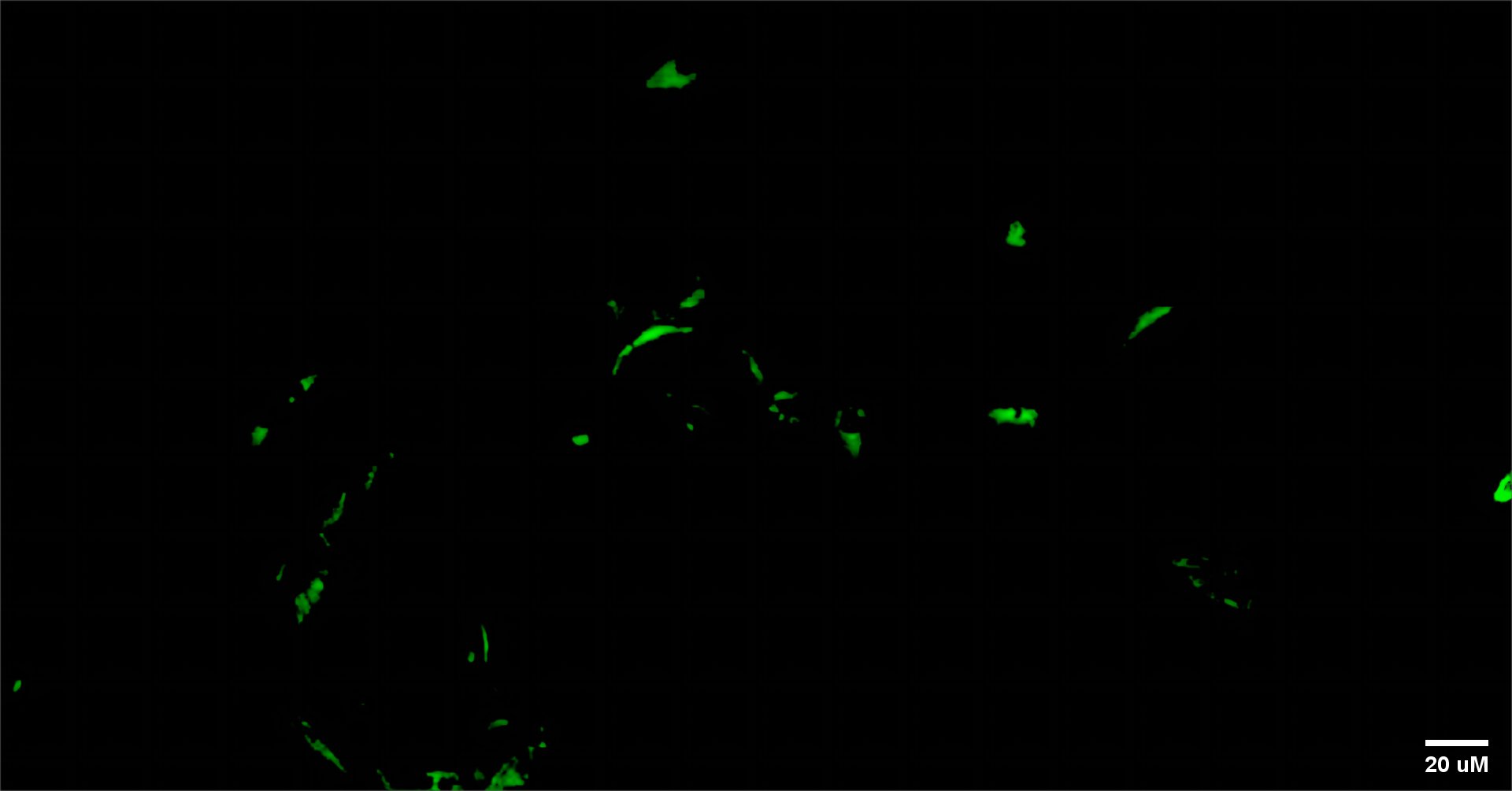

Supplement: Supplemental Information 5 [file peerj-13-20224-s005.zip › FIGURE4/FIG-4P-Q/FIG-4P/Control.jpg]

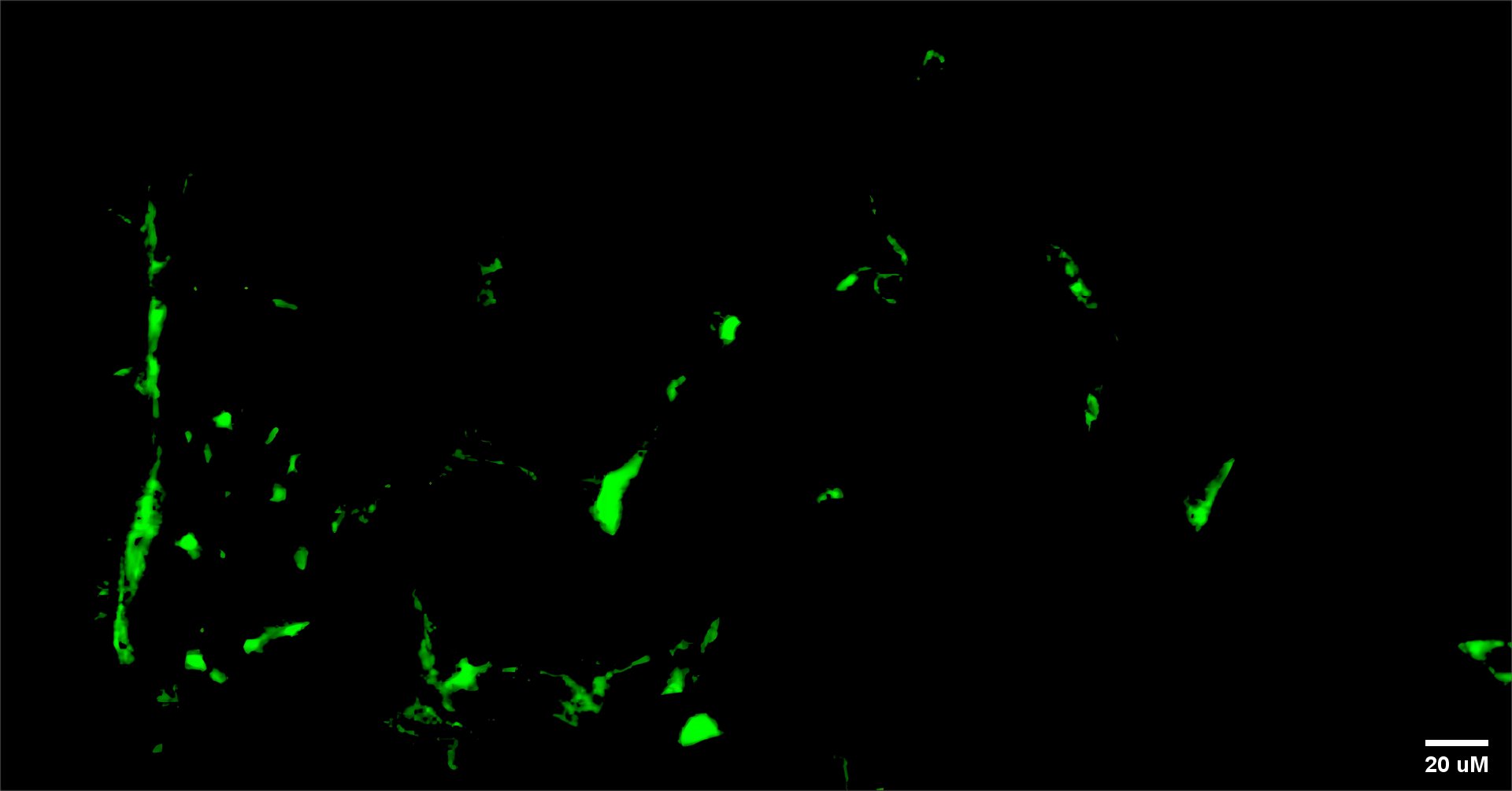

Supplement: Supplemental Information 5 [file peerj-13-20224-s005.zip › FIGURE4/FIG-4P-Q/FIG-4P/OE-NAT10.jpg]

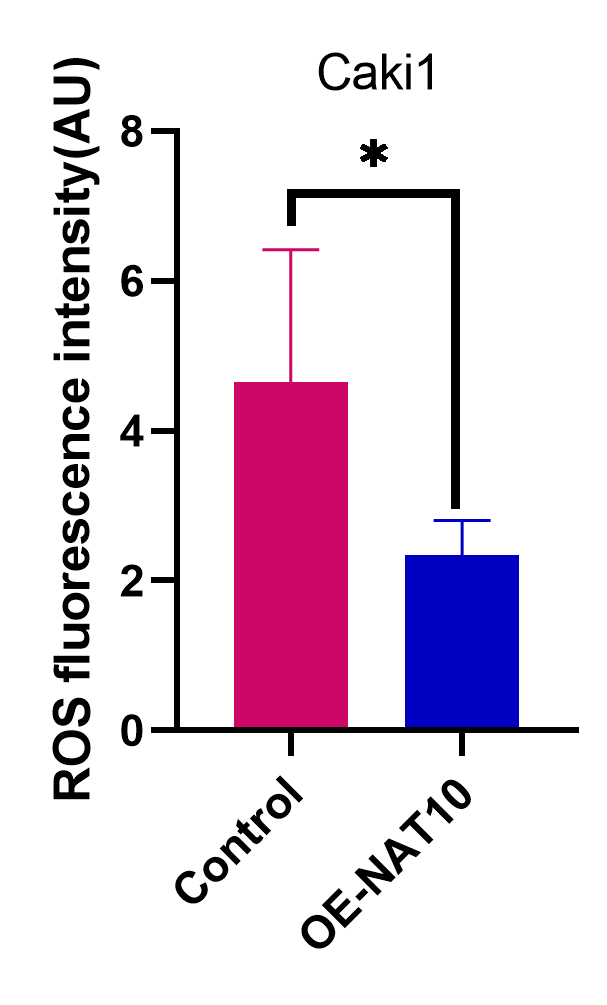

Supplement: Supplemental Information 5 [file peerj-13-20224-s005.zip › FIGURE4/FIG-4P-Q/FIG-4Q.tif]

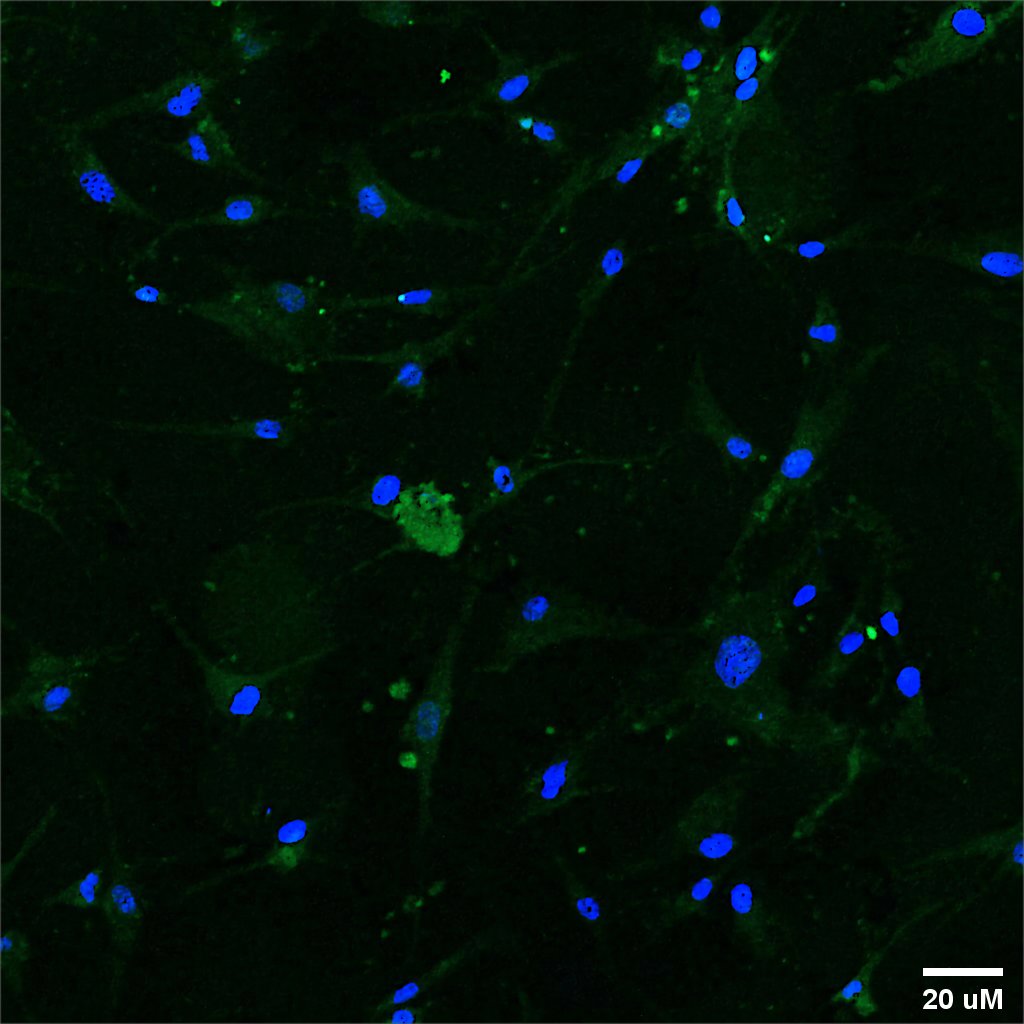

Supplement: Supplemental Information 6 [file peerj-13-20224-s006.zip › FIGURE5/FIG-5-E-F/FIG-5E/GPX4-Control.jpg]

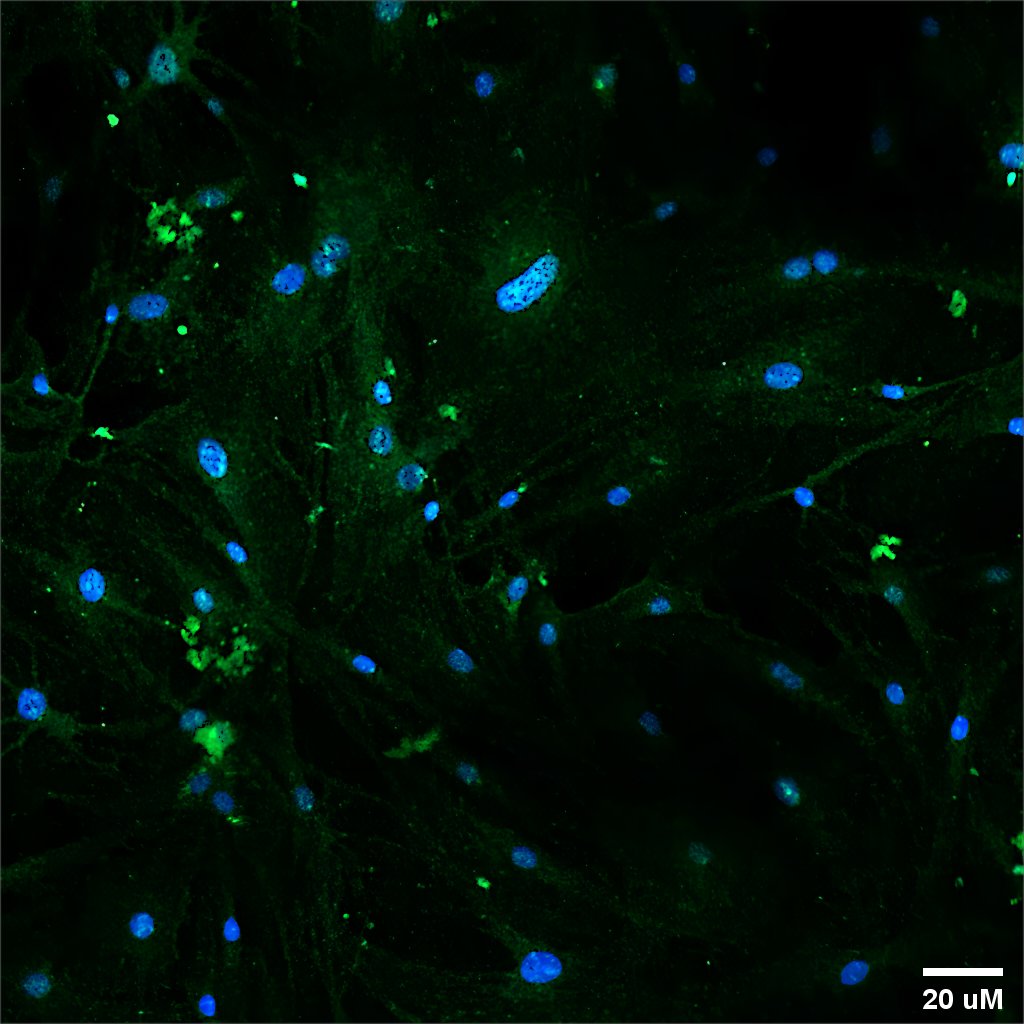

Supplement: Supplemental Information 6 [file peerj-13-20224-s006.zip › FIGURE5/FIG-5-E-F/FIG-5E/GPX4-OE.jpg]

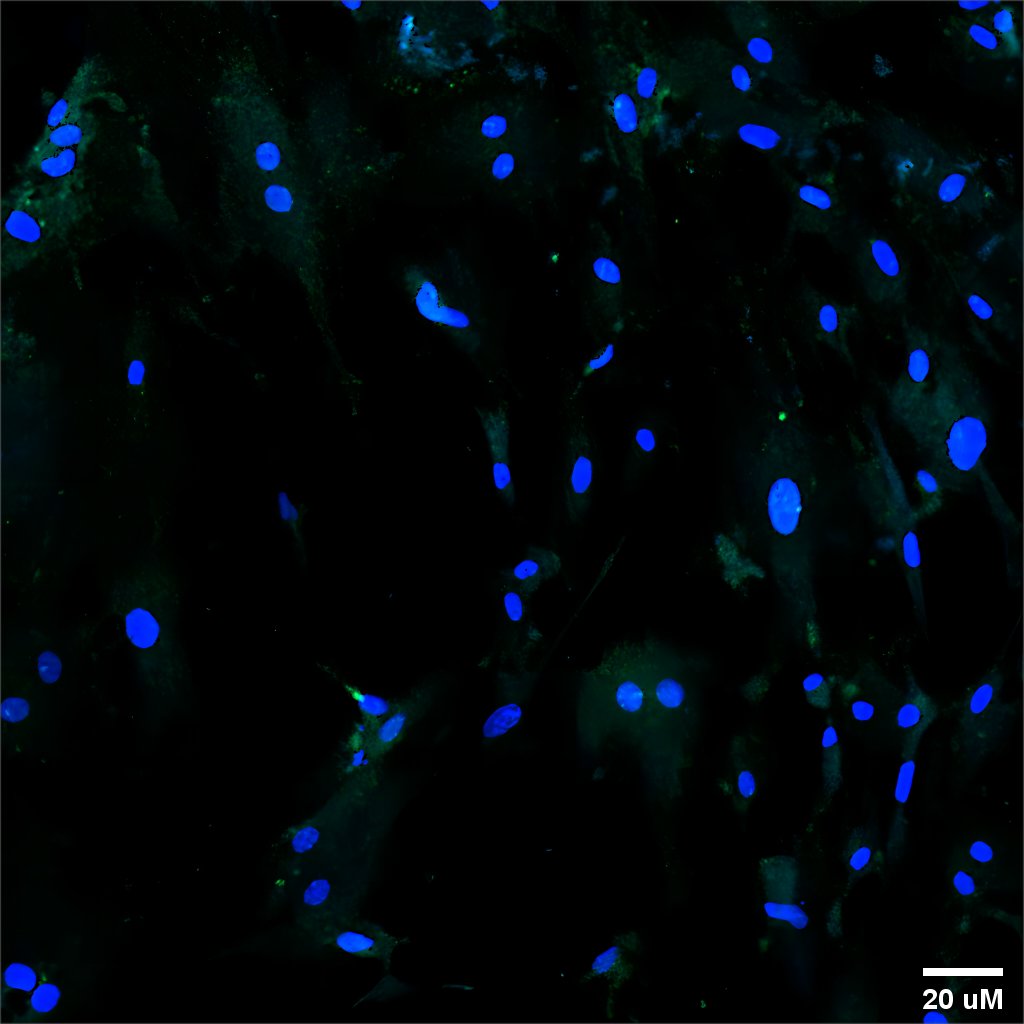

Supplement: Supplemental Information 6 [file peerj-13-20224-s006.zip › FIGURE5/FIG-5-E-F/FIG-5E/GPX4-sh.jpg]

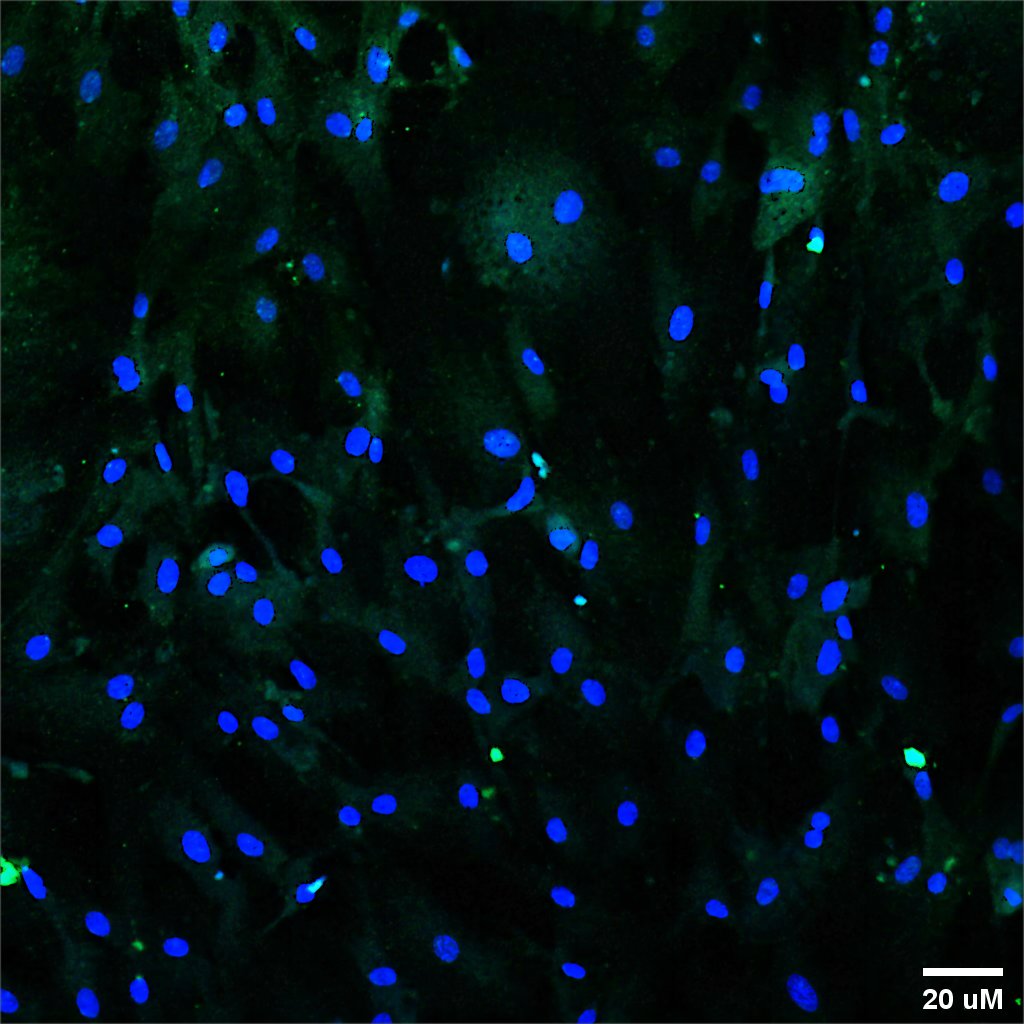

Supplement: Supplemental Information 6 [file peerj-13-20224-s006.zip › FIGURE5/FIG-5-E-F/FIG-5E/NFE2L1-Control.jpg]

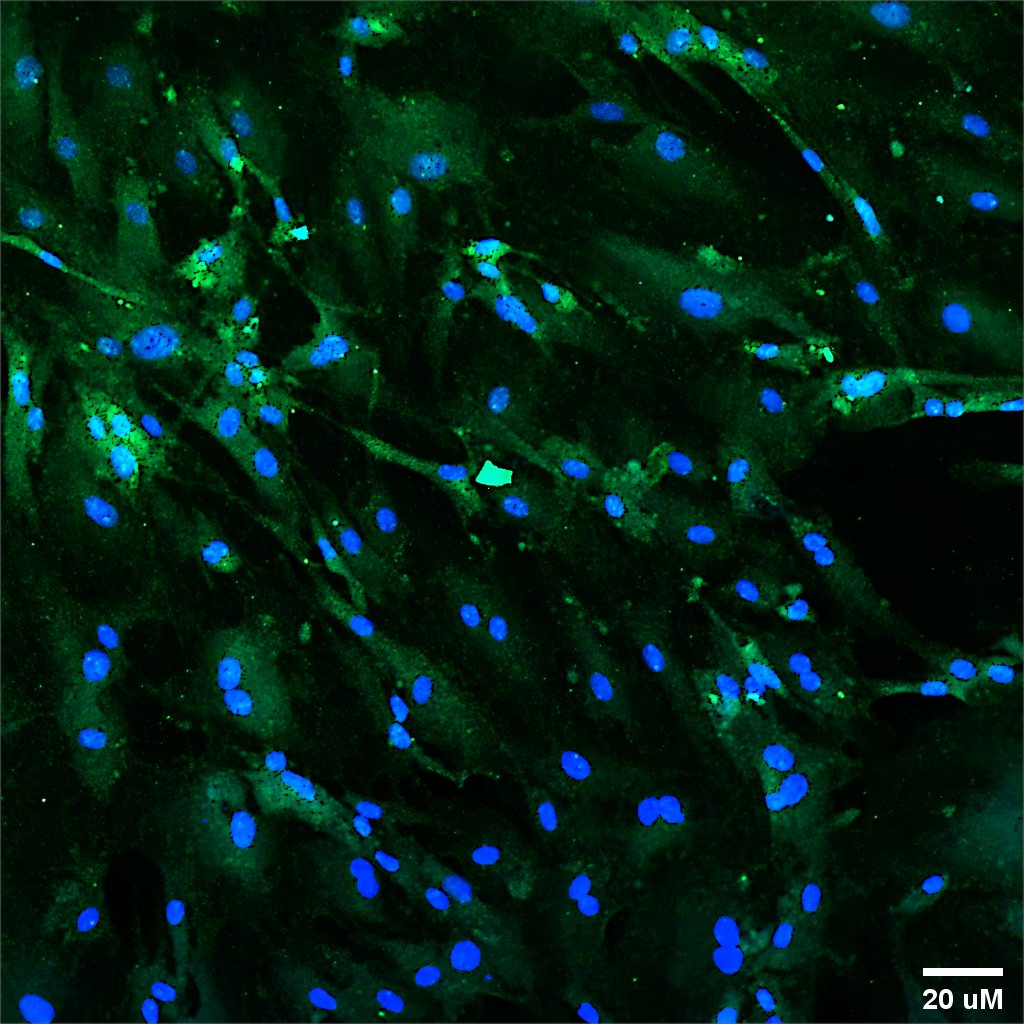

Supplement: Supplemental Information 6 [file peerj-13-20224-s006.zip › FIGURE5/FIG-5-E-F/FIG-5E/NFE2L1-OE.jpg]

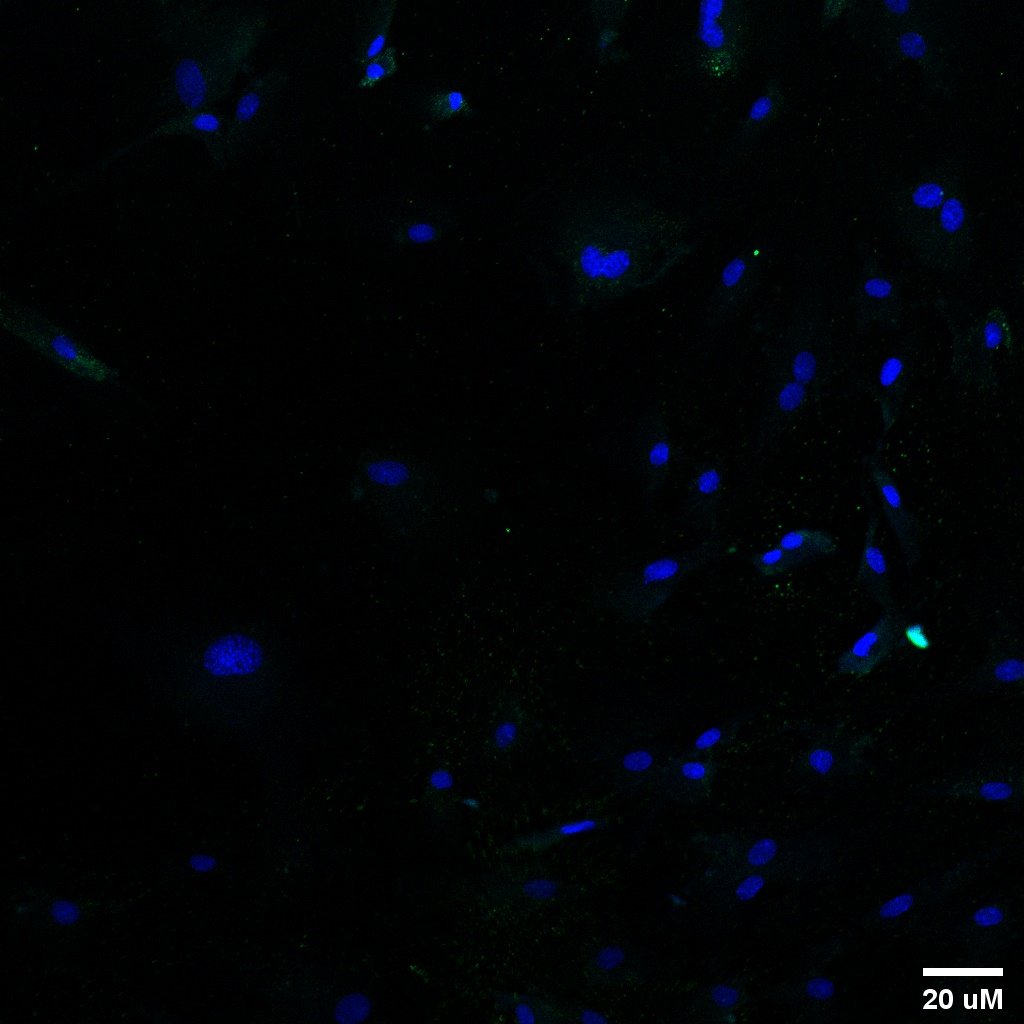

Supplement: Supplemental Information 6 [file peerj-13-20224-s006.zip › FIGURE5/FIG-5-E-F/FIG-5E/NFE2L1-sh.jpg]

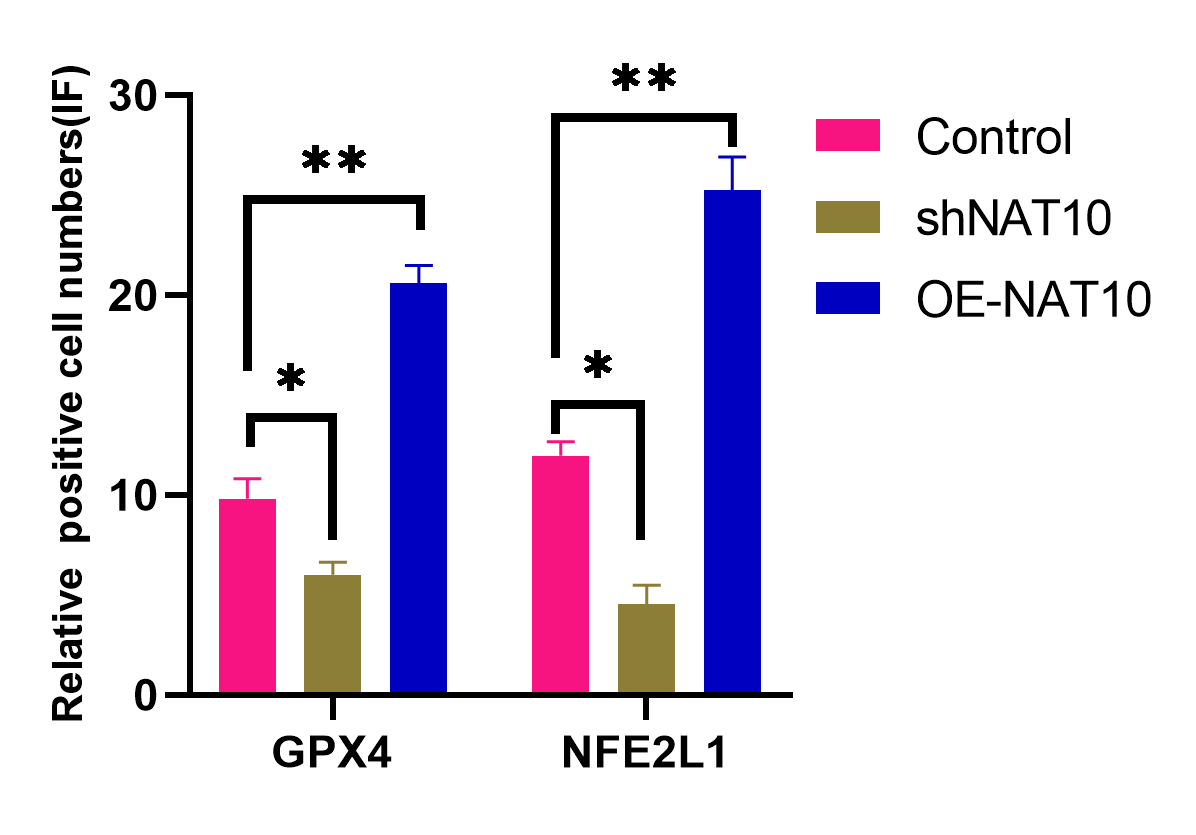

Supplement: Supplemental Information 6 [file peerj-13-20224-s006.zip › FIGURE5/FIG-5-E-F/FIG-5F.tif]

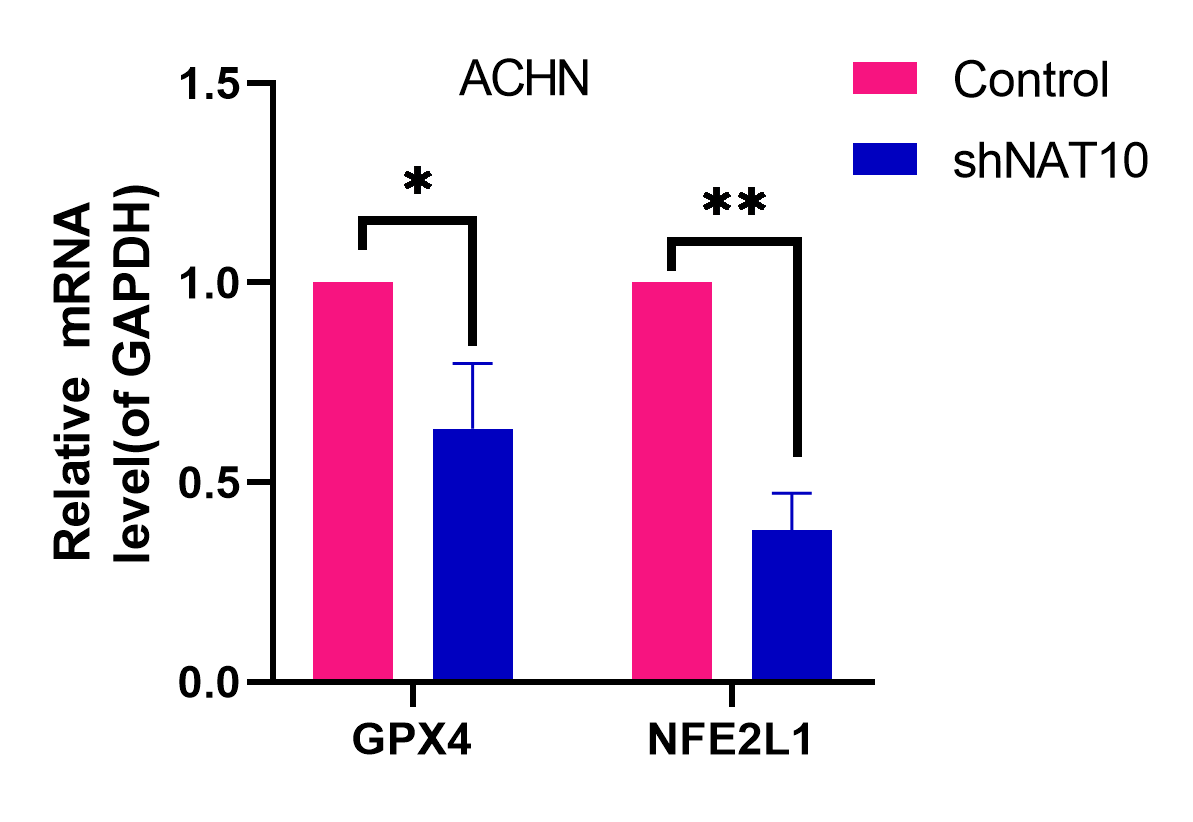

Supplement: Supplemental Information 6 [file peerj-13-20224-s006.zip › FIGURE5/FIG-5A/FIG-5A.tif]

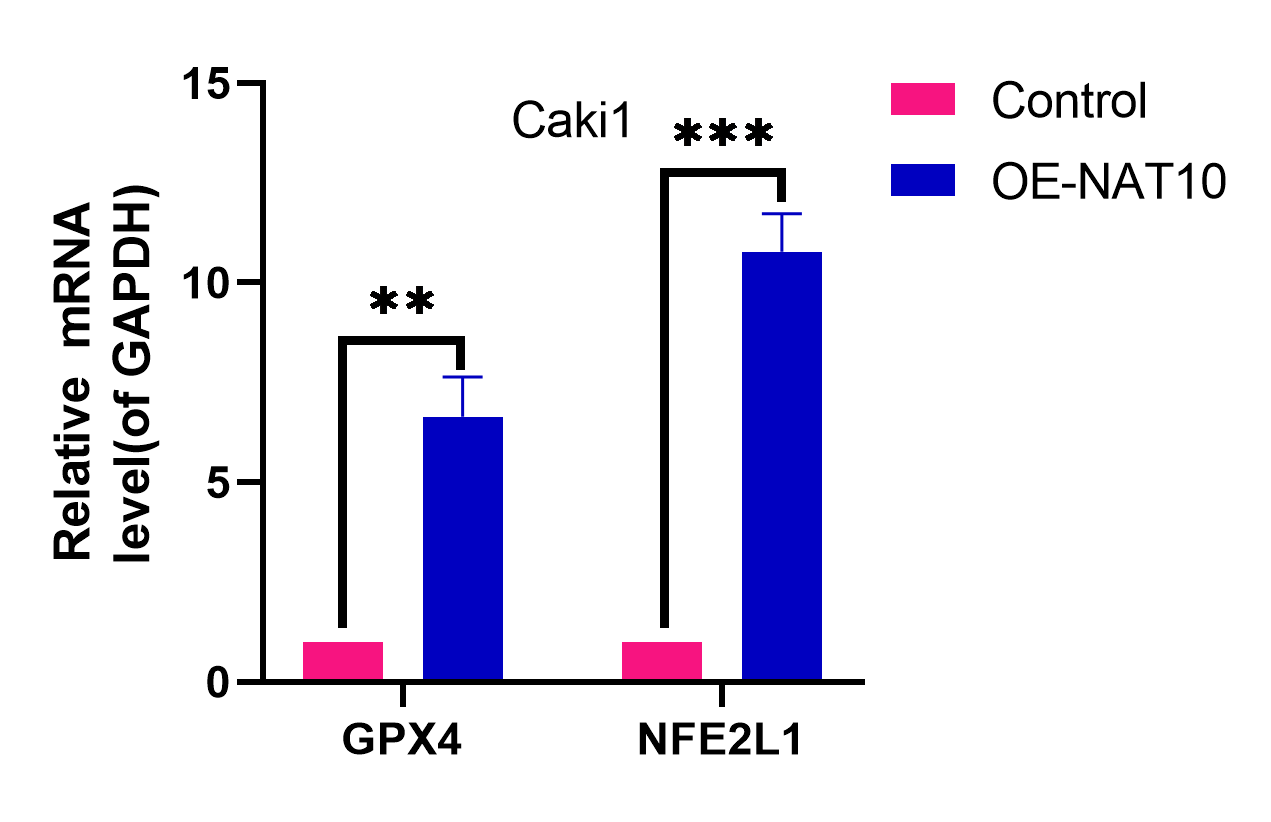

Supplement: Supplemental Information 6 [file peerj-13-20224-s006.zip › FIGURE5/FIG-5B/FIG-5B.tif]

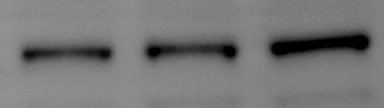

Supplement: Supplemental Information 6 [file peerj-13-20224-s006.zip › FIGURE5/FIG-5C-D/1.tif]

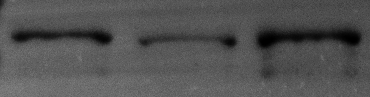

Supplement: Supplemental Information 6 [file peerj-13-20224-s006.zip › FIGURE5/FIG-5C-D/2.tif]

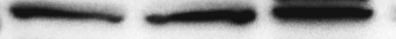

Supplement: Supplemental Information 6 [file peerj-13-20224-s006.zip › FIGURE5/FIG-5C-D/3.tif]

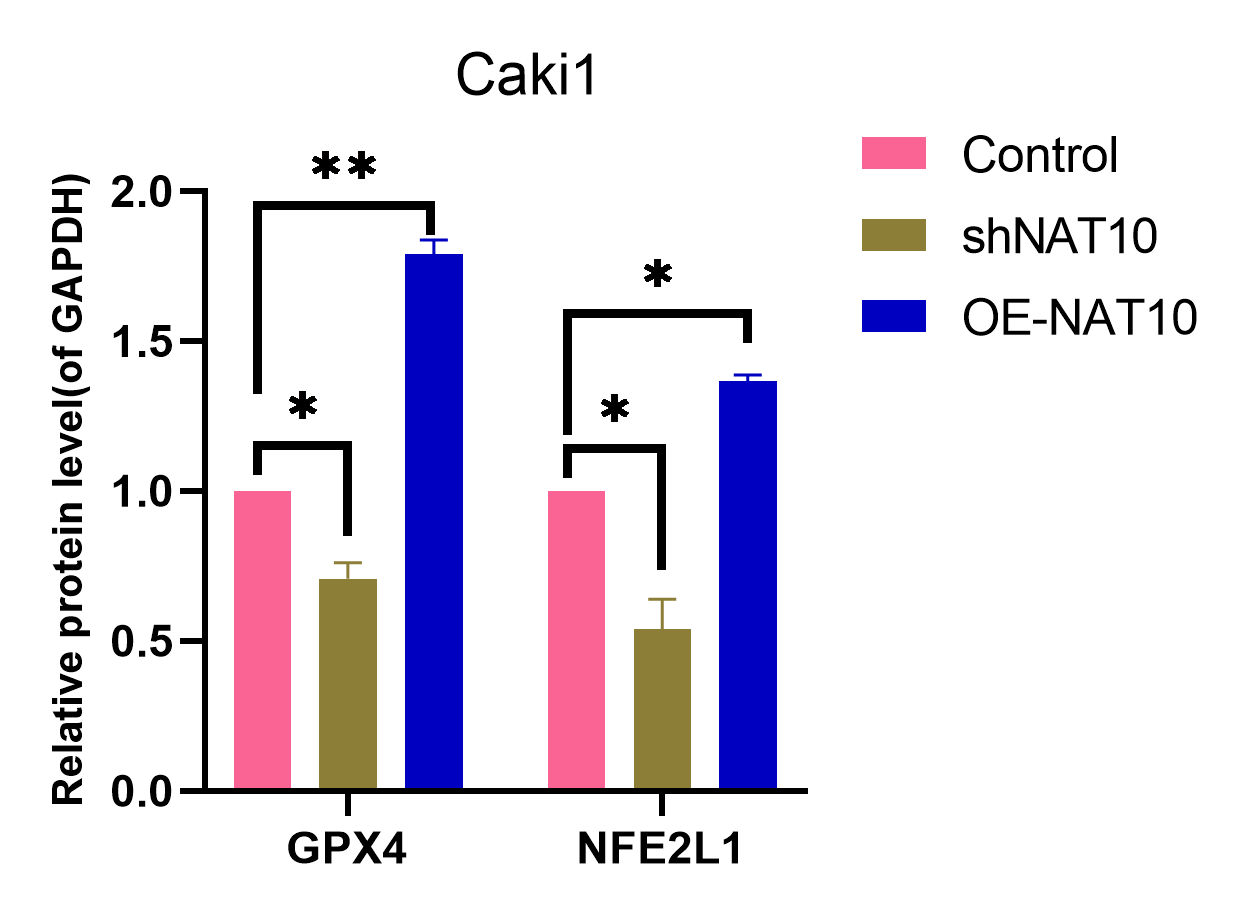

Supplement: Supplemental Information 6 [file peerj-13-20224-s006.zip › FIGURE5/FIG-5C-D/Fig.5D.tif]

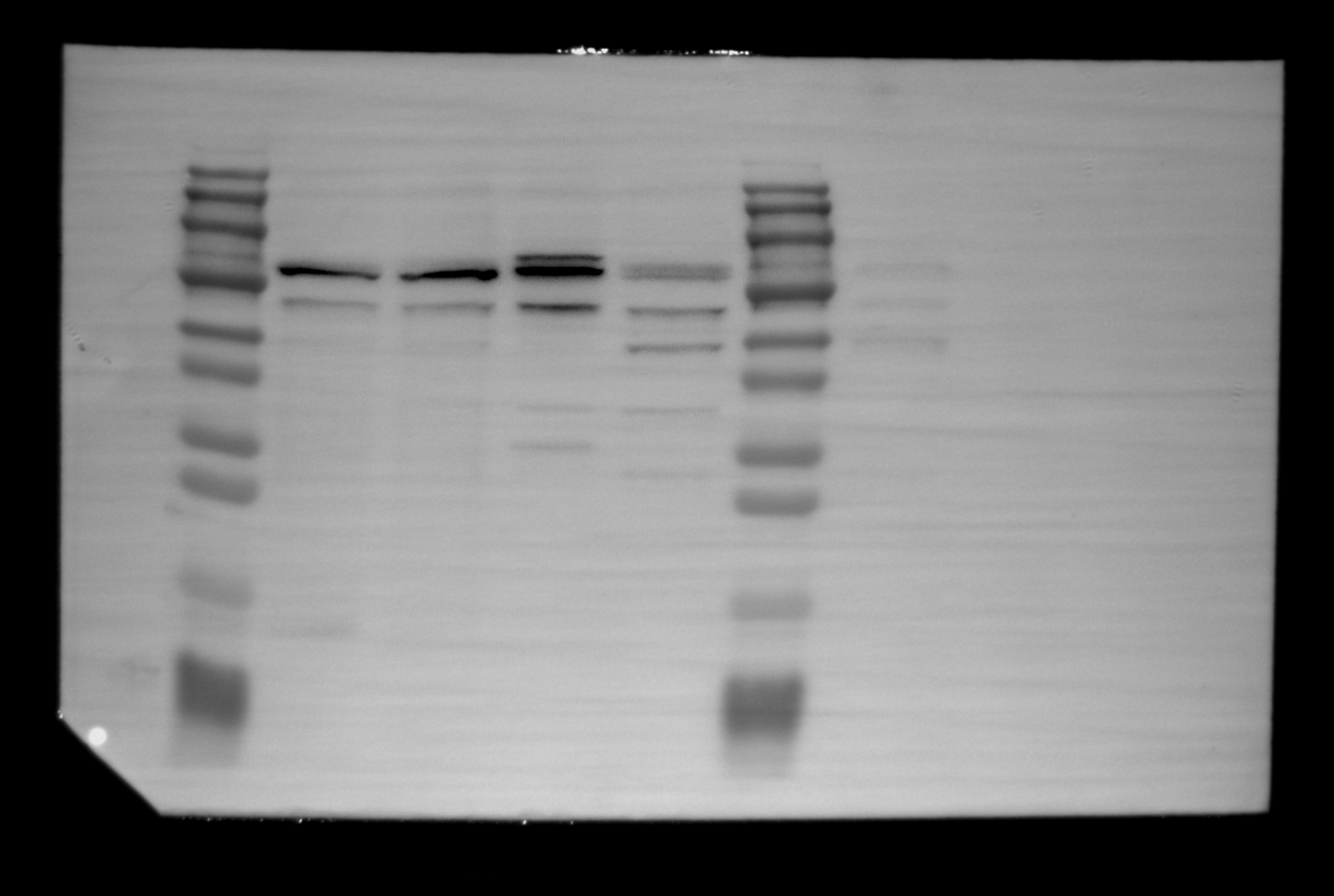

Supplement: Supplemental Information 6 [file peerj-13-20224-s006.zip › FIGURE5/FIG-5C-D/GAPDH.tif]

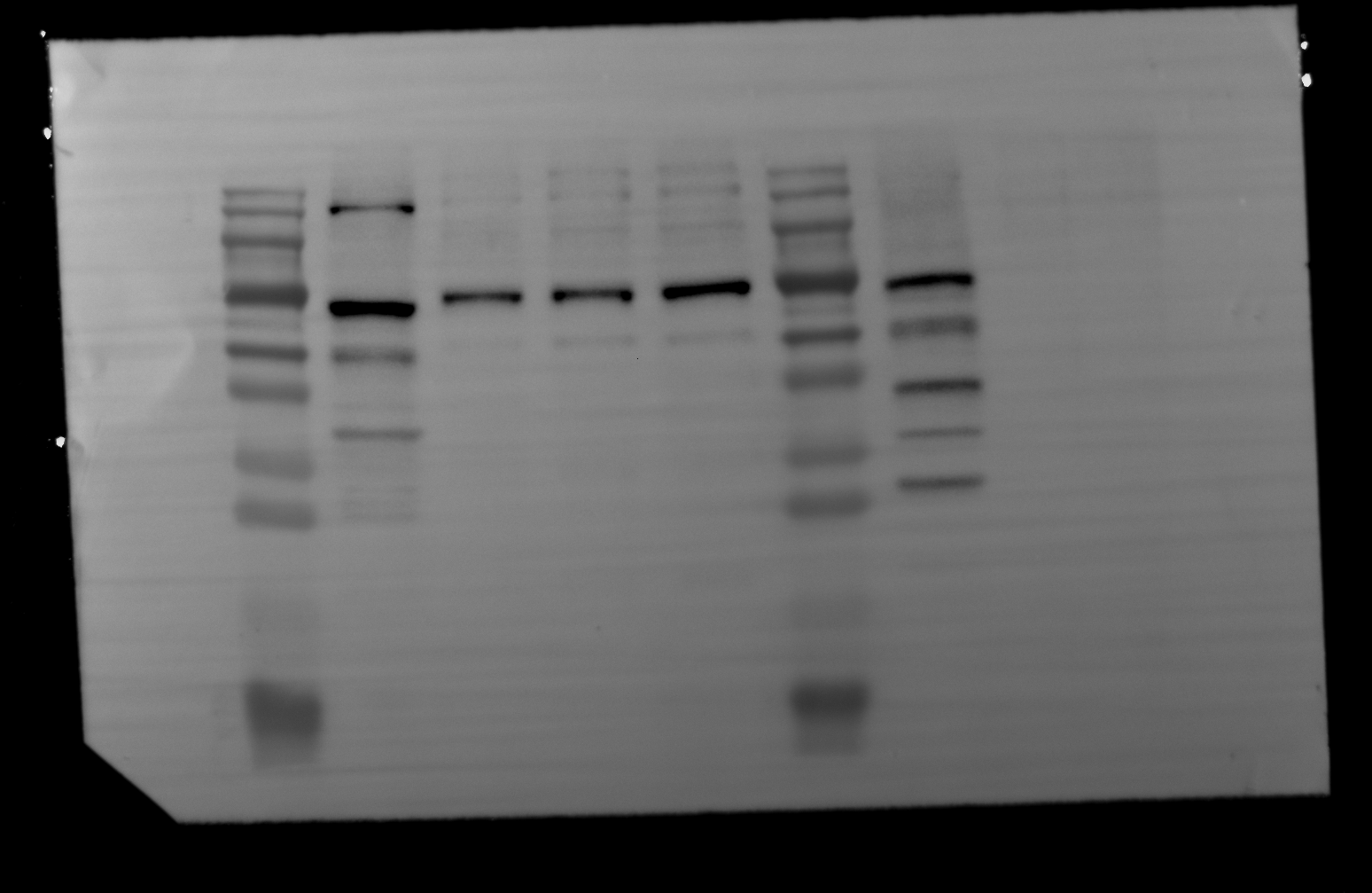

Supplement: Supplemental Information 6 [file peerj-13-20224-s006.zip › FIGURE5/FIG-5C-D/GPX4.tif]

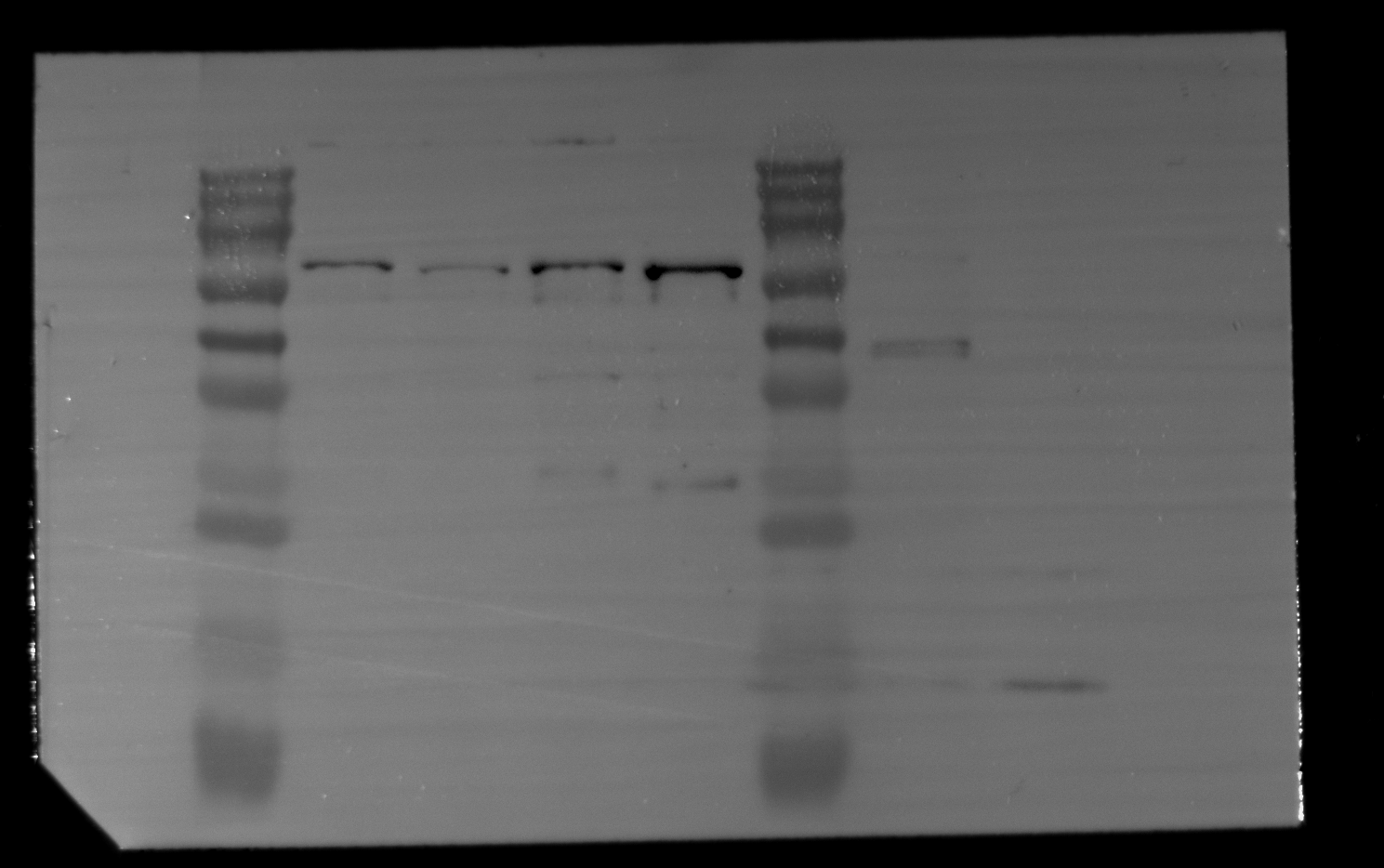

Supplement: Supplemental Information 6 [file peerj-13-20224-s006.zip › FIGURE5/FIG-5C-D/NFE2L1.tif]

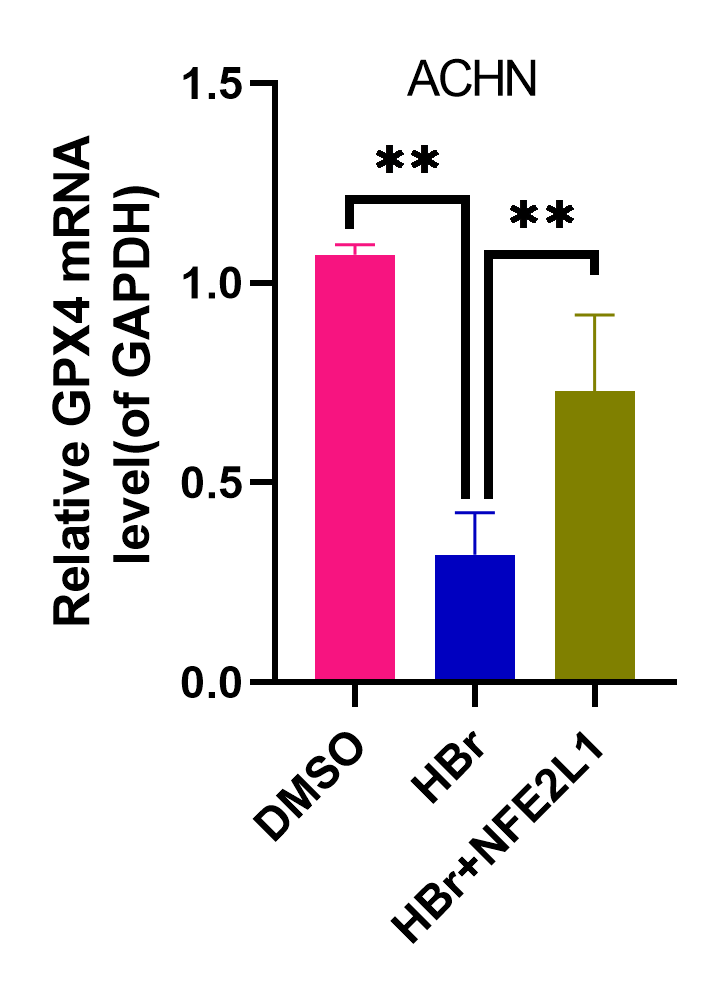

Supplement: Supplemental Information 7 [file peerj-13-20224-s007.zip › FIGURE6/FIG-6A-B/FIG-6A.tif]

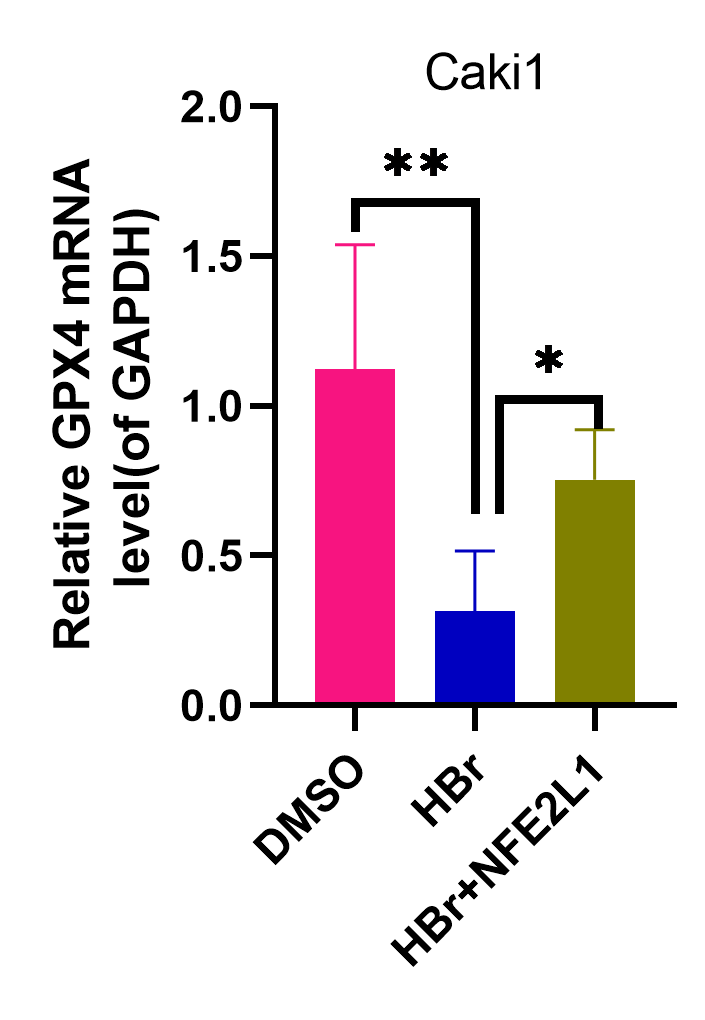

Supplement: Supplemental Information 7 [file peerj-13-20224-s007.zip › FIGURE6/FIG-6A-B/FIG-6B.tif]

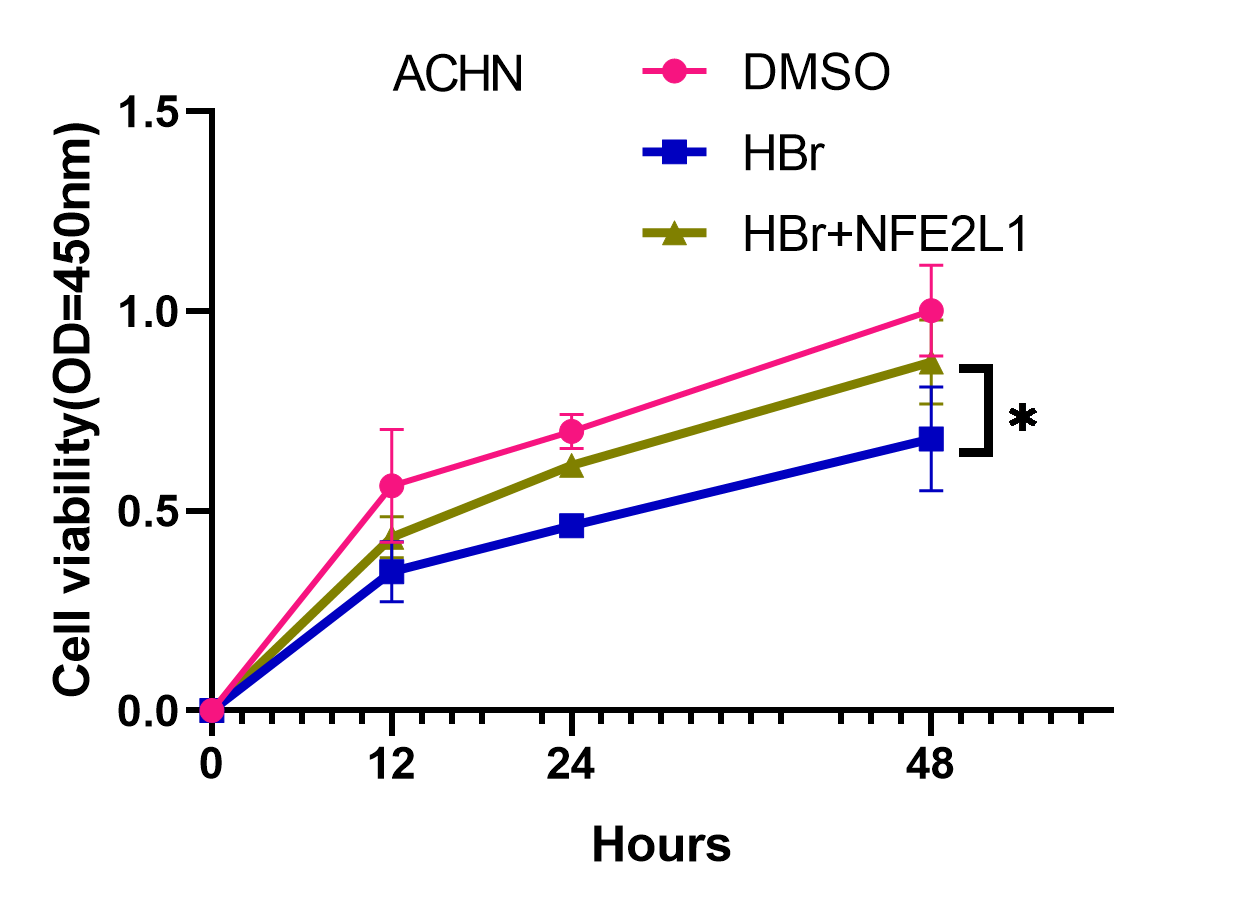

Supplement: Supplemental Information 7 [file peerj-13-20224-s007.zip › FIGURE6/FIG-6C-D/FIG-6C.tif]

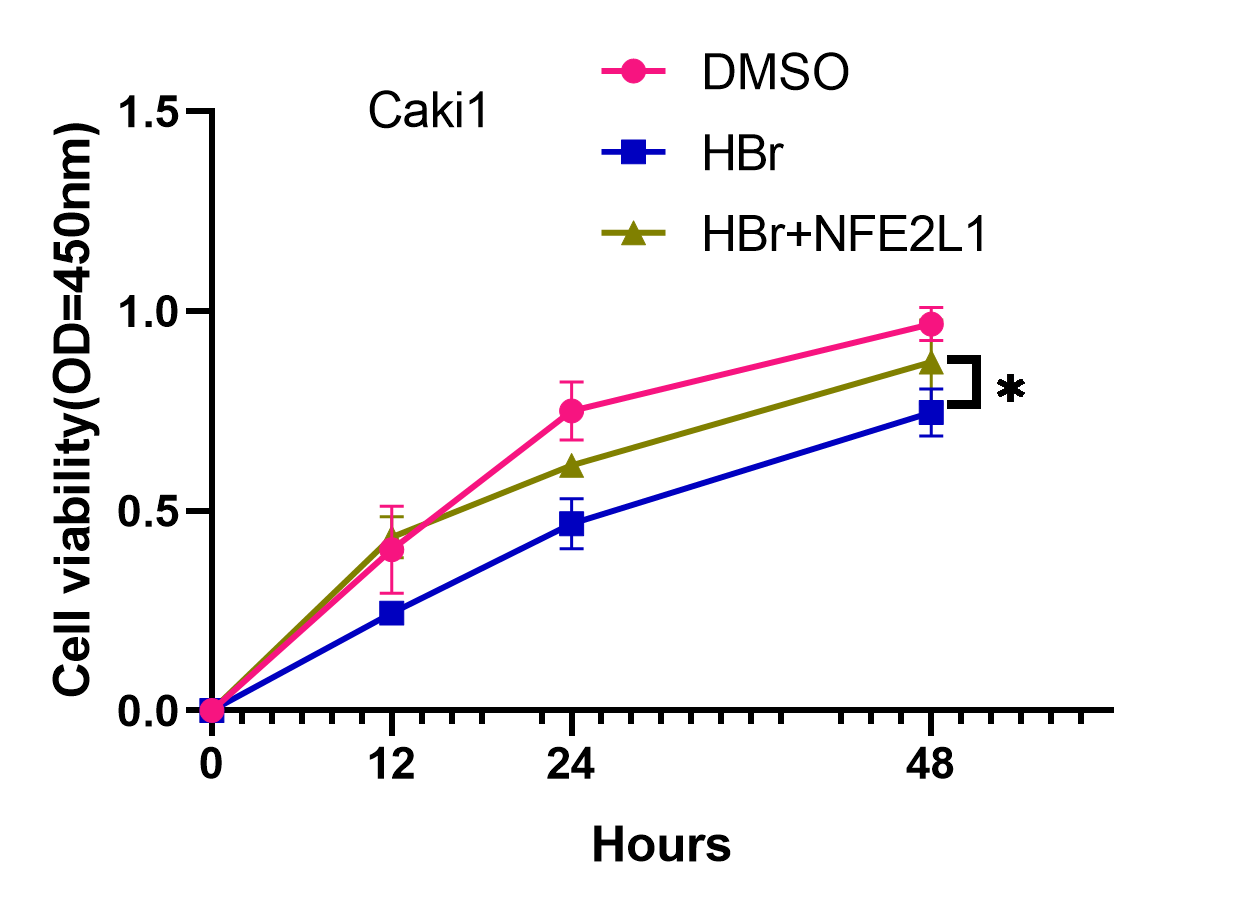

Supplement: Supplemental Information 7 [file peerj-13-20224-s007.zip › FIGURE6/FIG-6C-D/FIG-6D.tif]

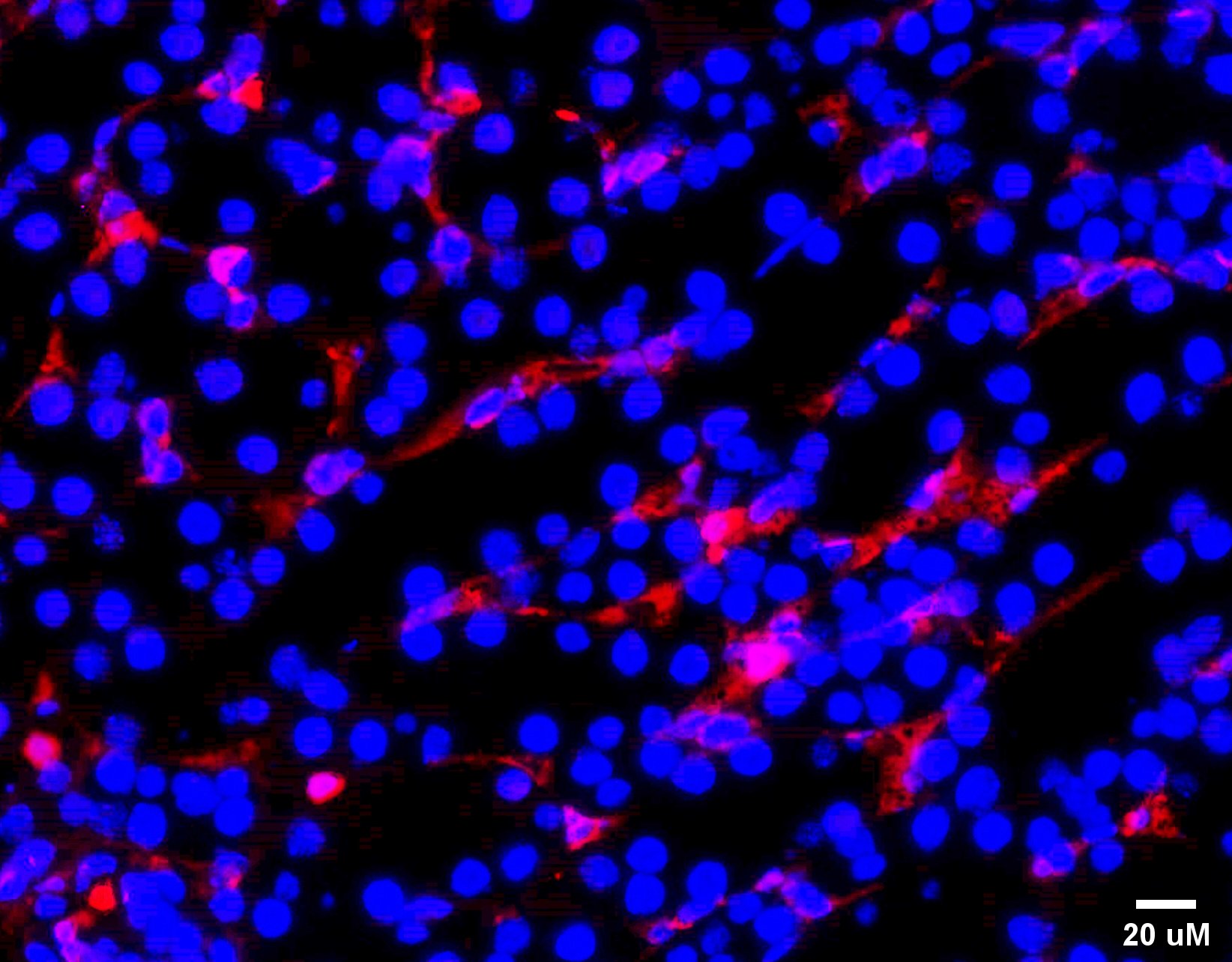

Supplement: Supplemental Information 7 [file peerj-13-20224-s007.zip › FIGURE6/FIG-6E/ACHN/GPX4/DMSO.jpg]

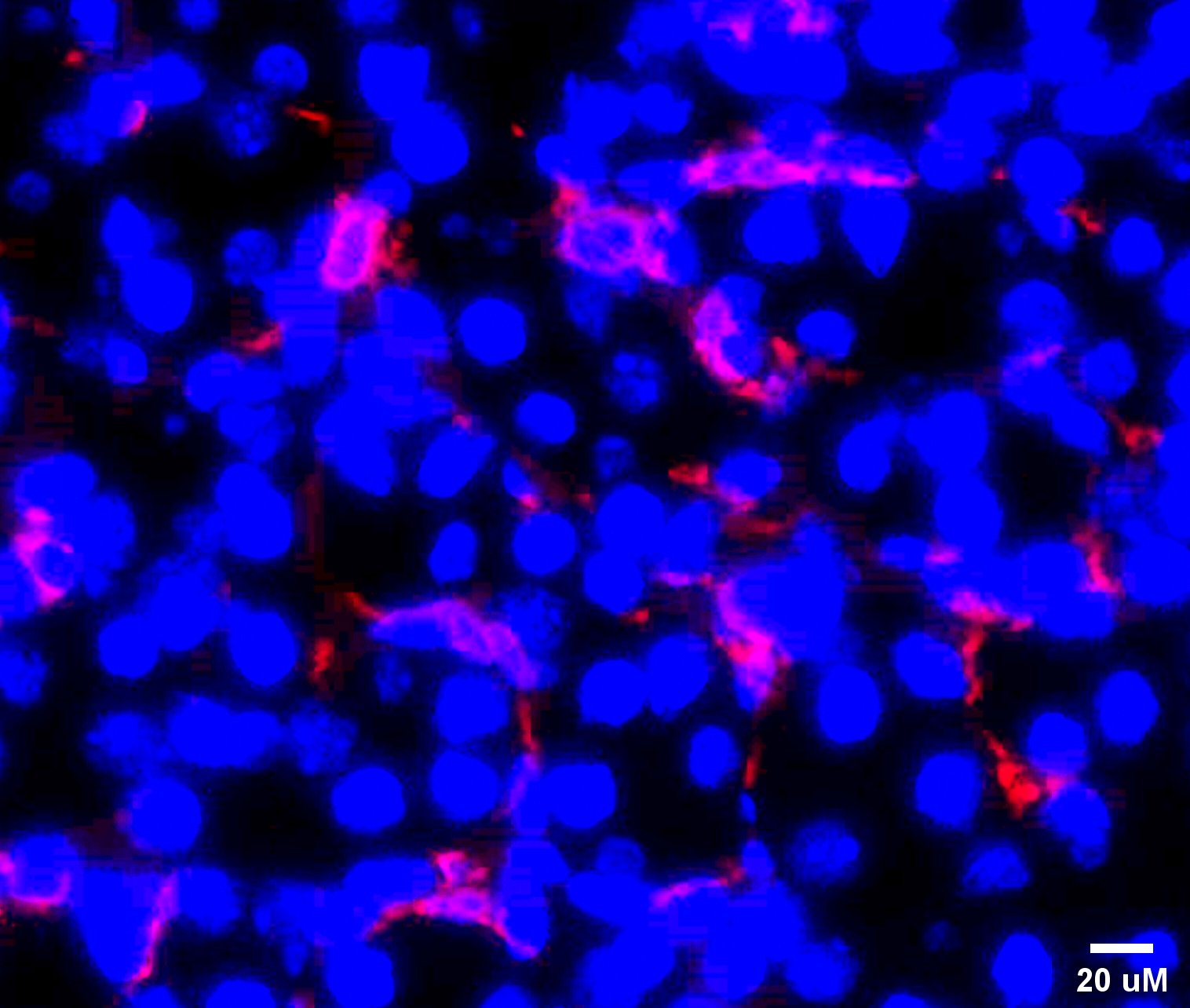

Supplement: Supplemental Information 7 [file peerj-13-20224-s007.zip › FIGURE6/FIG-6E/ACHN/GPX4/HBr+NFE2L1.jpg]

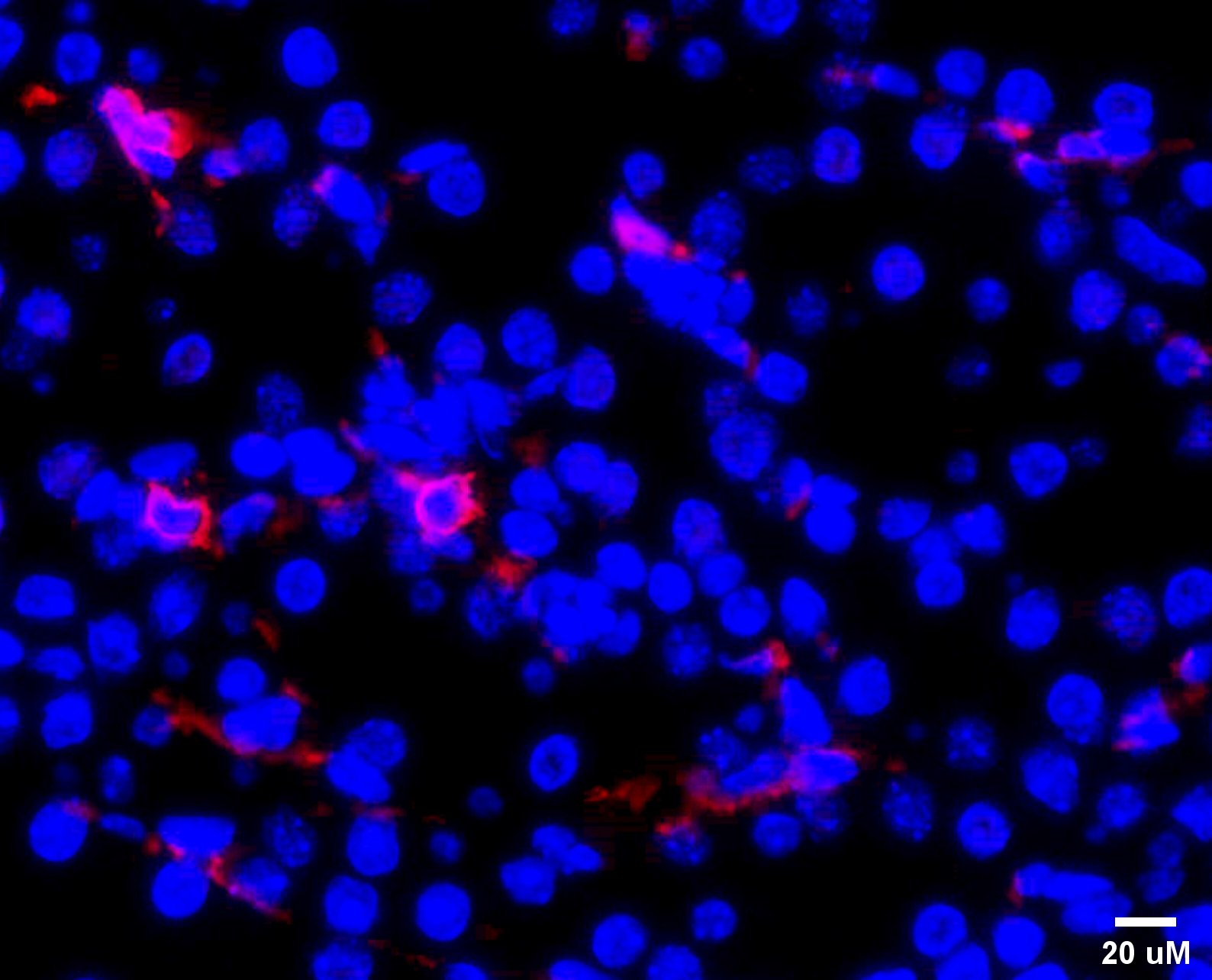

Supplement: Supplemental Information 7 [file peerj-13-20224-s007.zip › FIGURE6/FIG-6E/ACHN/GPX4/HBr.jpg]

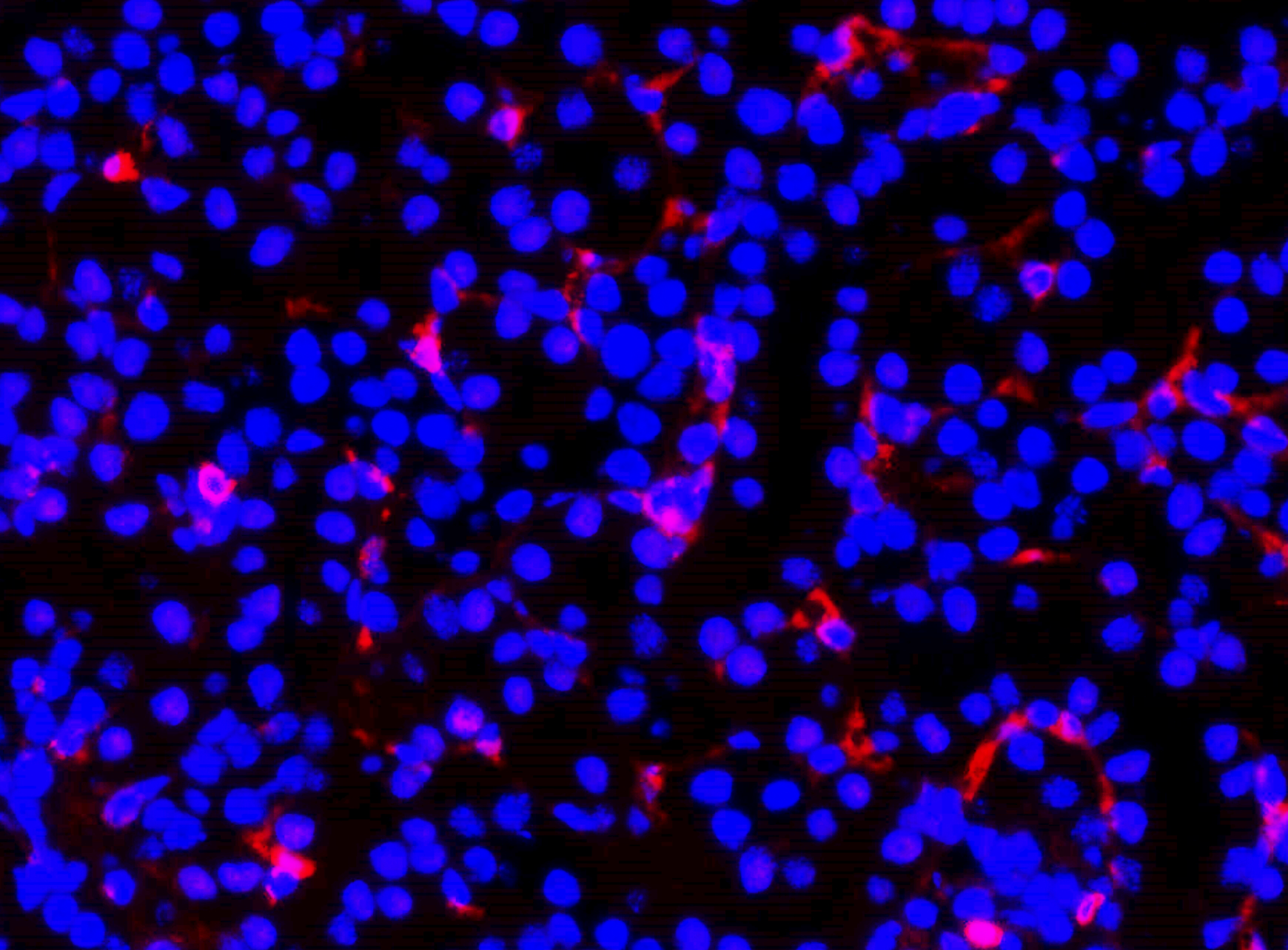

Supplement: Supplemental Information 7 [file peerj-13-20224-s007.zip › FIGURE6/FIG-6E/CAKI1/GPX4/DMSO.tif]

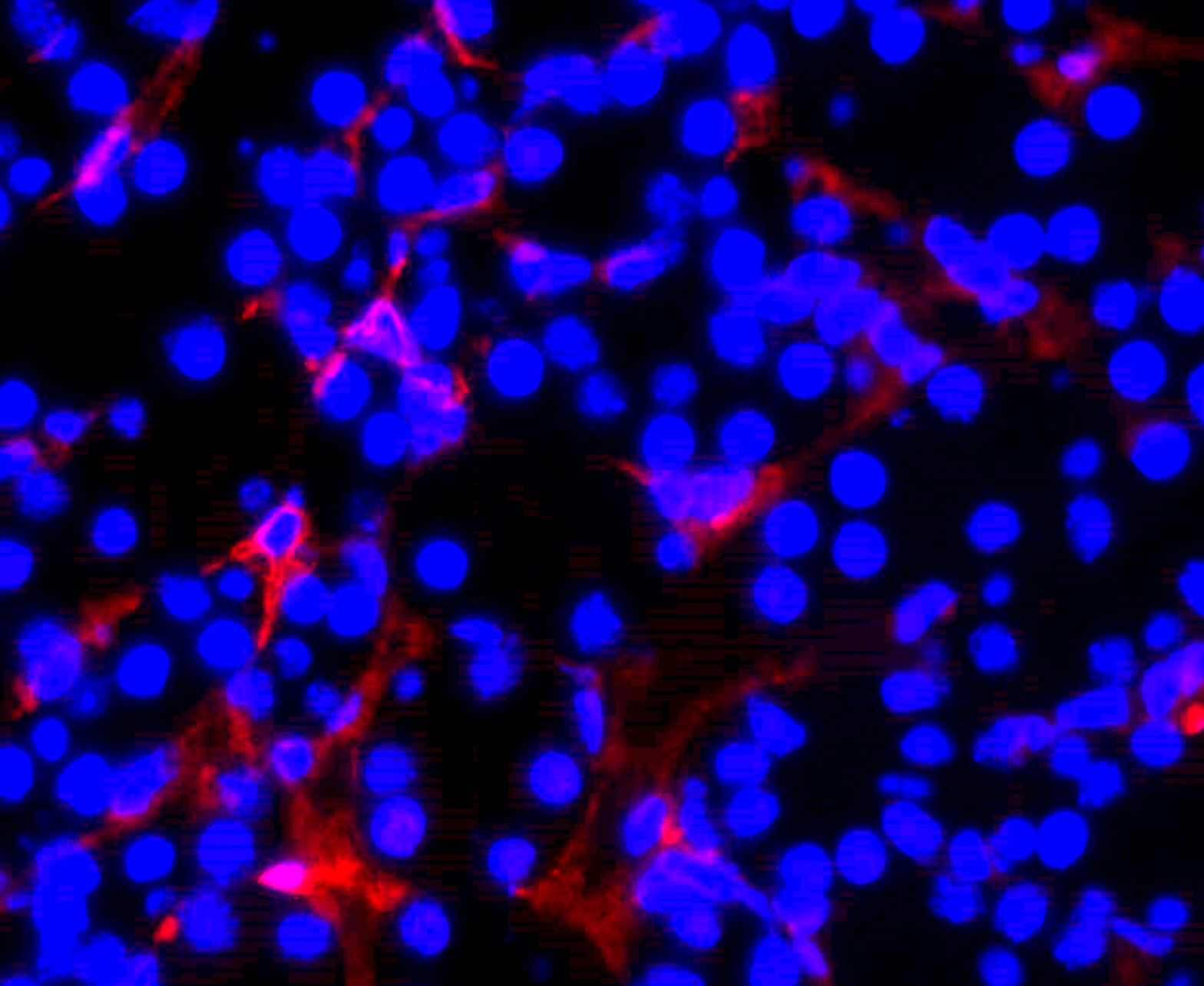

Supplement: Supplemental Information 7 [file peerj-13-20224-s007.zip › FIGURE6/FIG-6E/CAKI1/GPX4/HBr+NFE2L1.tif]

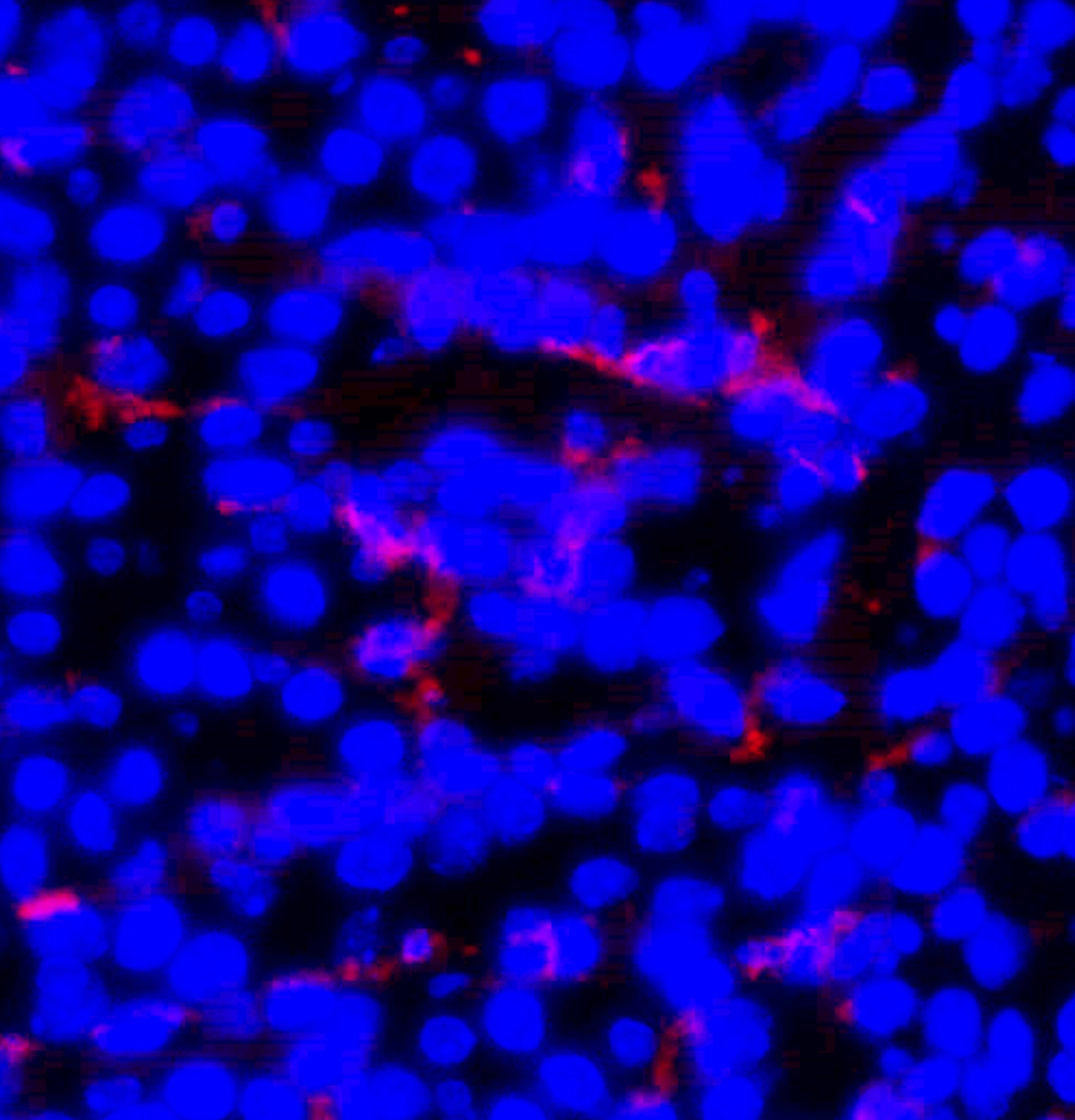

Supplement: Supplemental Information 7 [file peerj-13-20224-s007.zip › FIGURE6/FIG-6E/CAKI1/GPX4/HBr.tif]

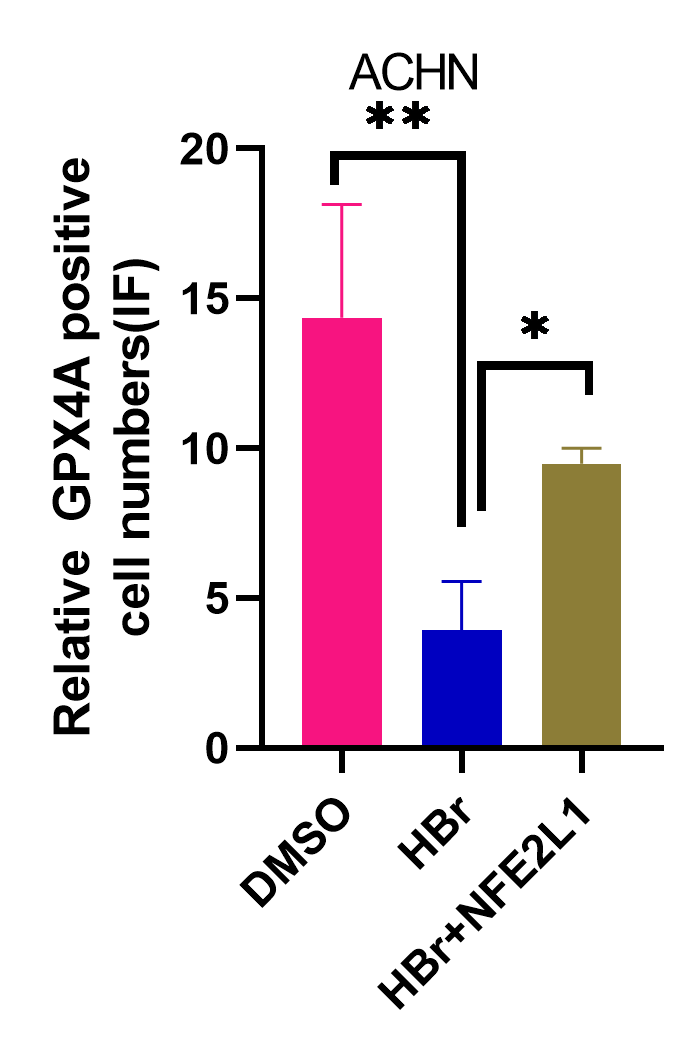

Supplement: Supplemental Information 7 [file peerj-13-20224-s007.zip › FIGURE6/FIG-6F/FIG-6F.tif]

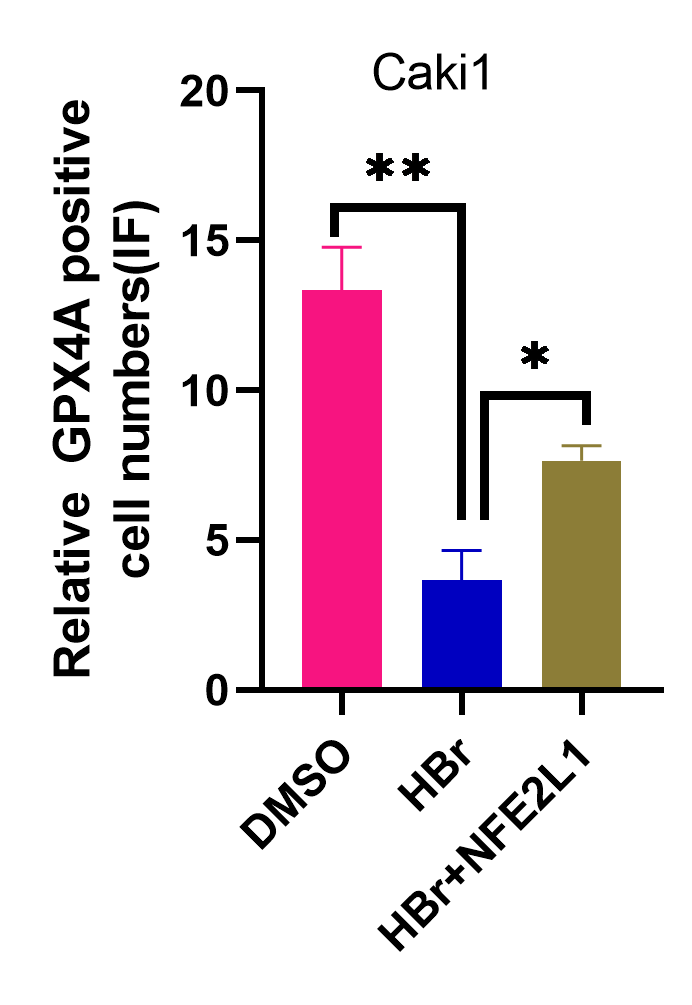

Supplement: Supplemental Information 7 [file peerj-13-20224-s007.zip › FIGURE6/FIG-6G/Fig.6G.tif]

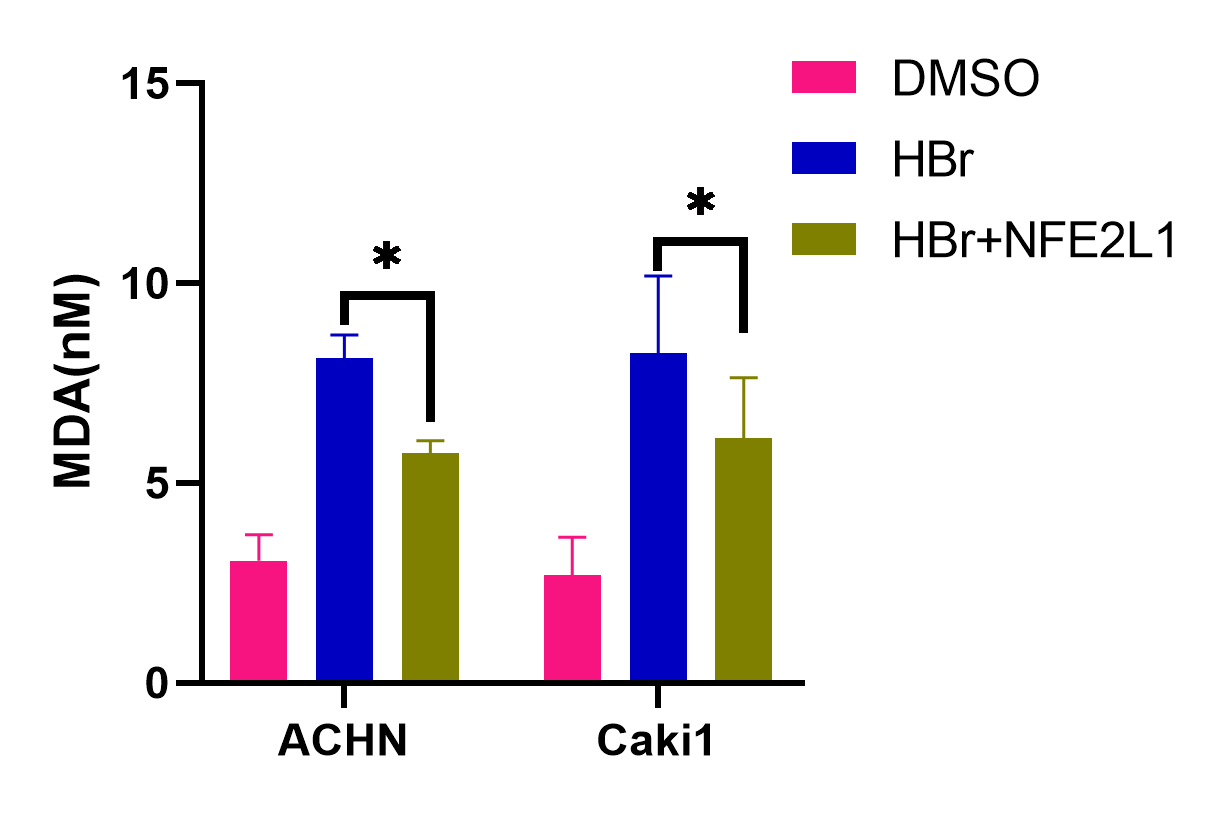

Supplement: Supplemental Information 7 [file peerj-13-20224-s007.zip › FIGURE6/FIG-6H/FIG-6H.tif]

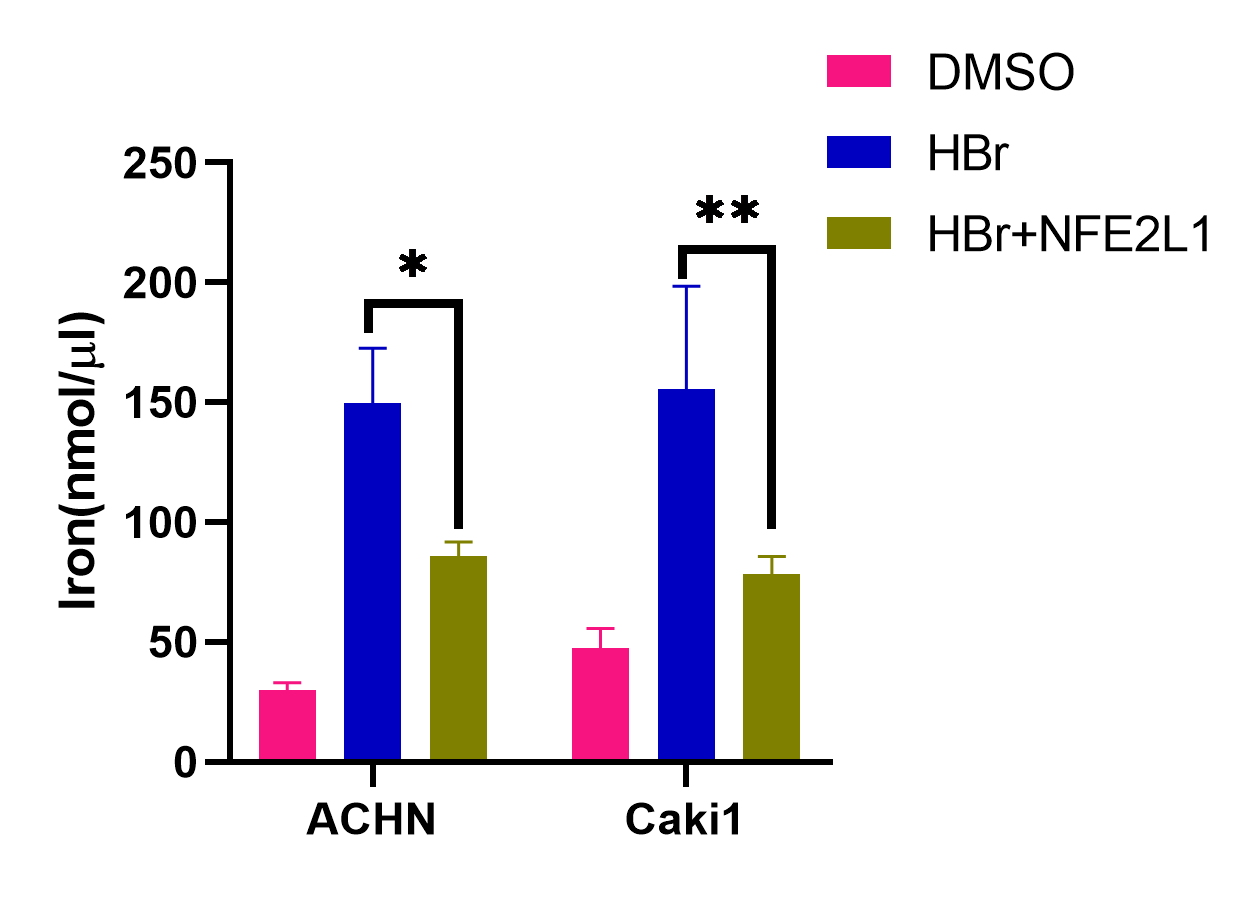

Supplement: Supplemental Information 7 [file peerj-13-20224-s007.zip › FIGURE6/FIG-6I/FIG-6I.tif]

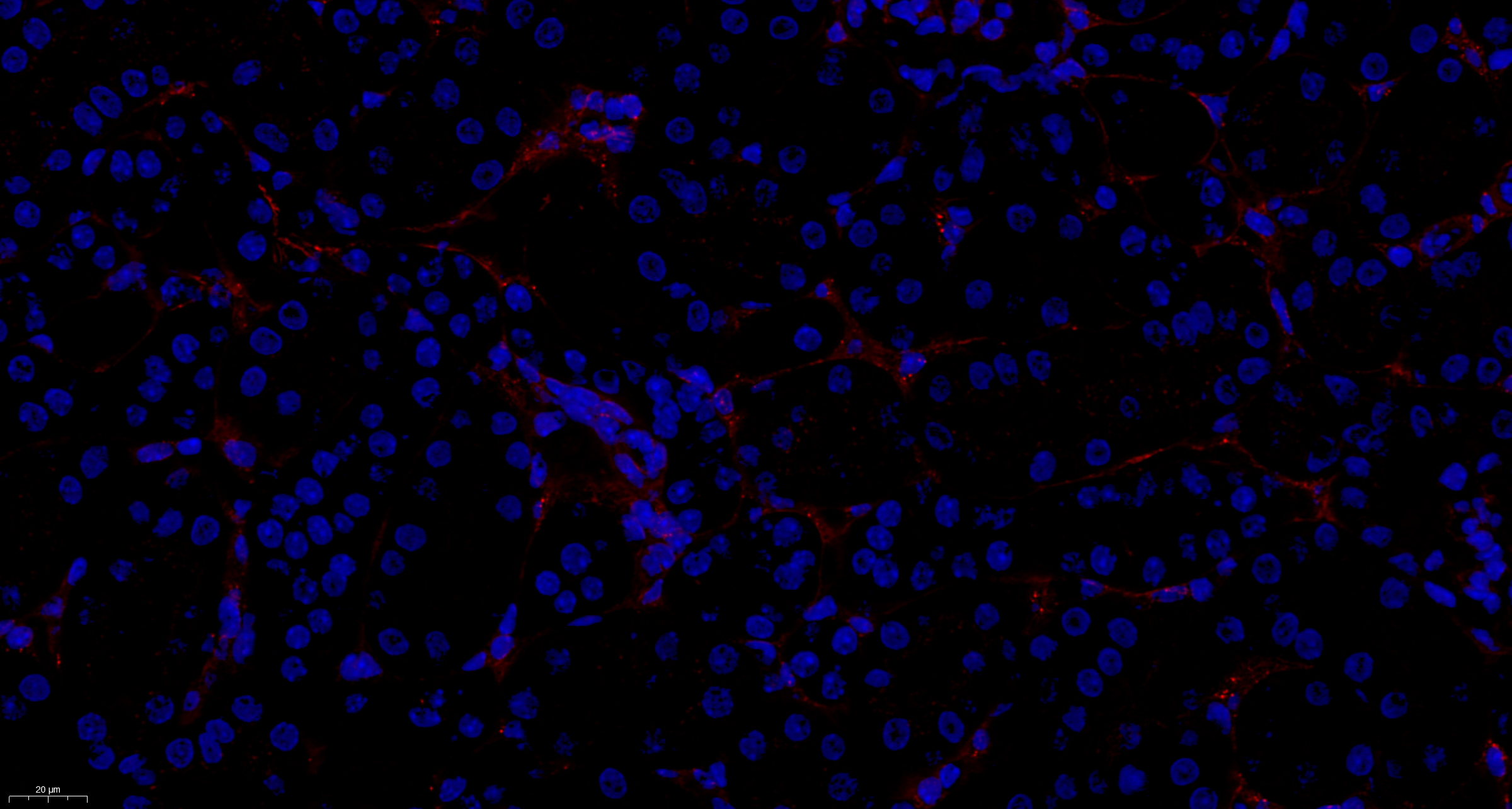

Supplement: Supplemental Information 7 [file peerj-13-20224-s007.zip › FIGURE6/FIG-6J/ACHN--CHAC1/ACHN/DMSO.jpg]

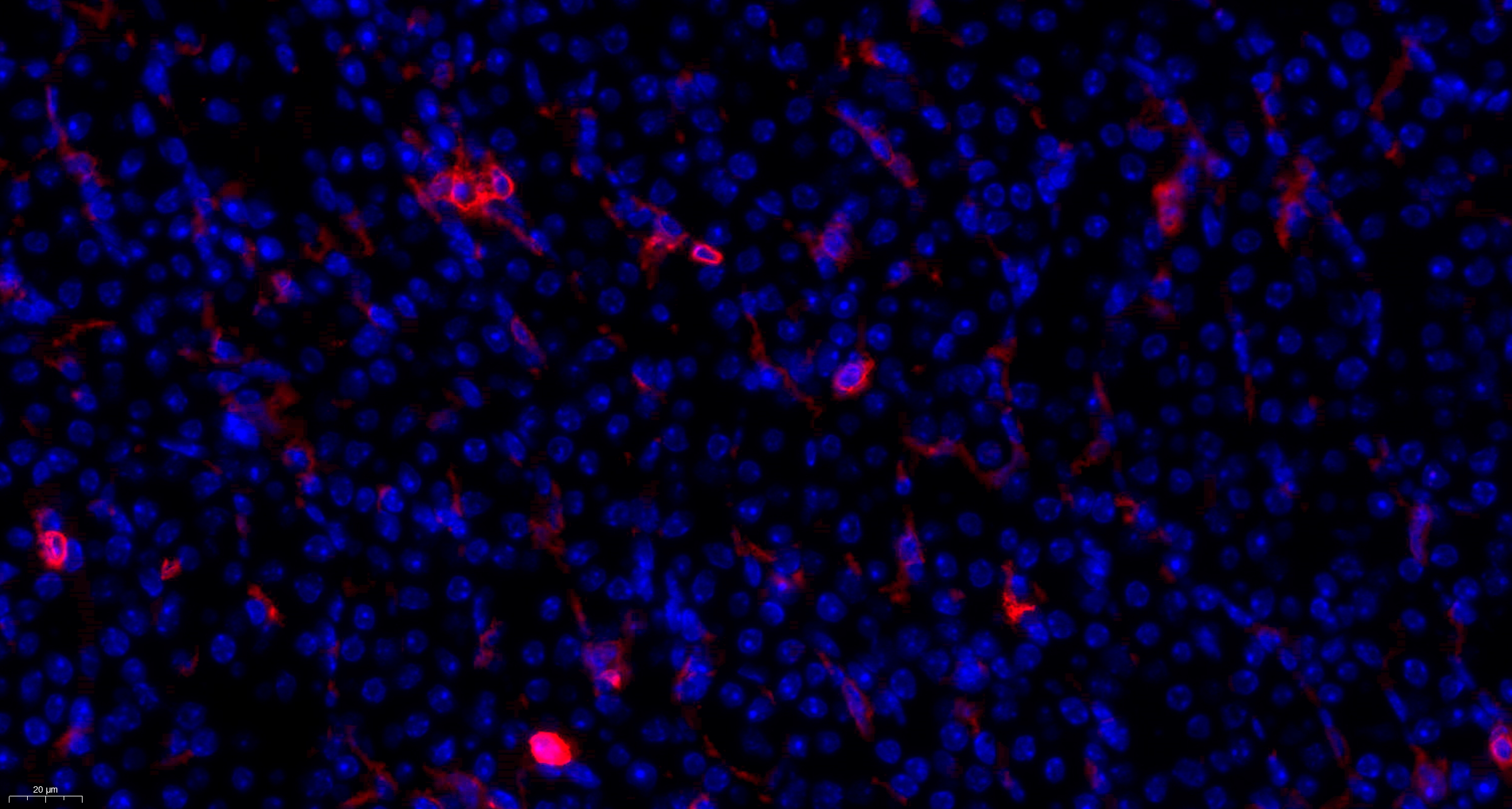

Supplement: Supplemental Information 7 [file peerj-13-20224-s007.zip › FIGURE6/FIG-6J/ACHN--CHAC1/ACHN/HBR+NFE.jpg]

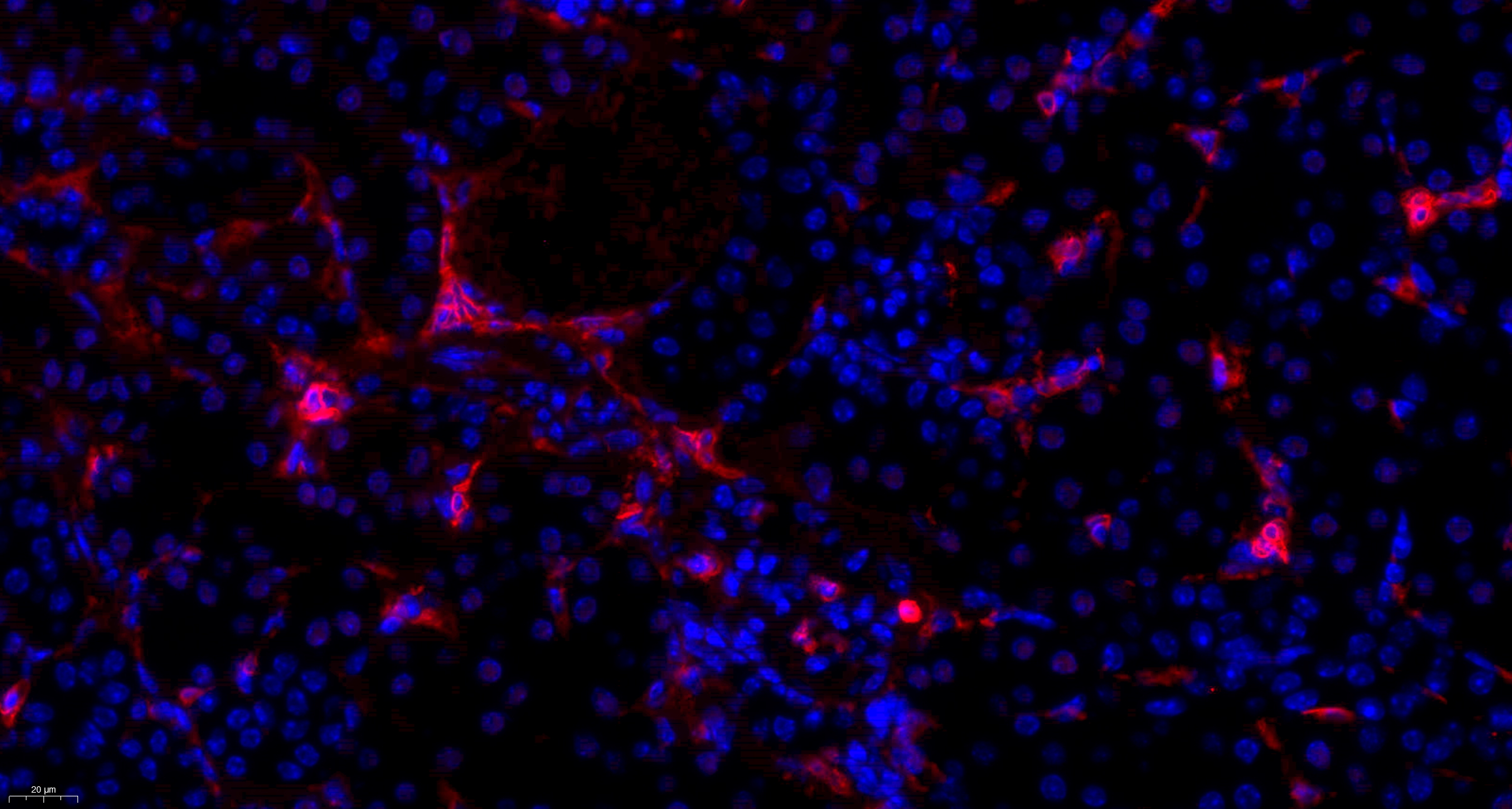

Supplement: Supplemental Information 7 [file peerj-13-20224-s007.zip › FIGURE6/FIG-6J/ACHN--CHAC1/ACHN/HBr.jpg]

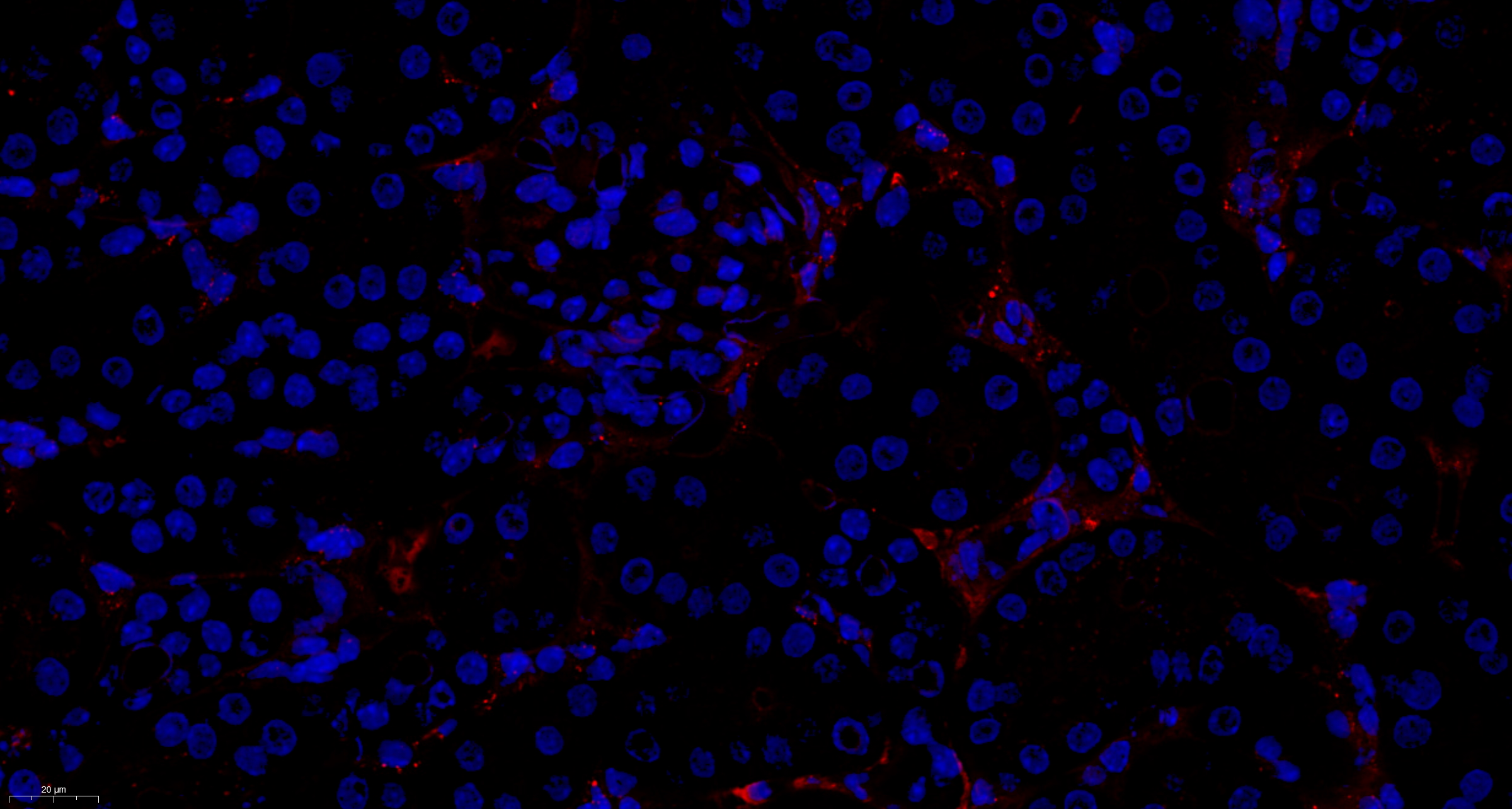

Supplement: Supplemental Information 7 [file peerj-13-20224-s007.zip › FIGURE6/FIG-6J/ACHN--PTGS2/ACHN/DMSO.jpg]

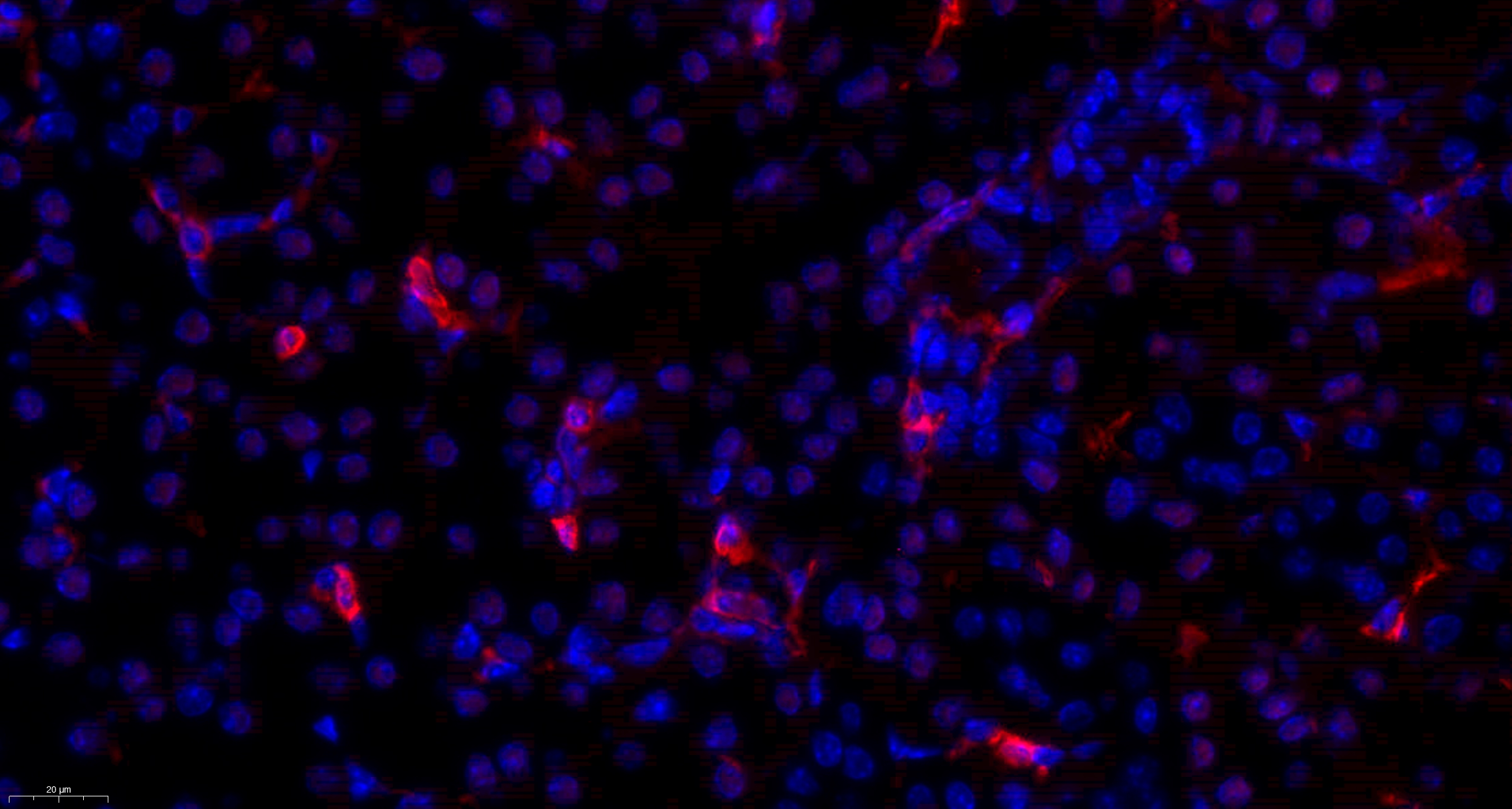

Supplement: Supplemental Information 7 [file peerj-13-20224-s007.zip › FIGURE6/FIG-6J/ACHN--PTGS2/ACHN/HBR+NFE.jpg]

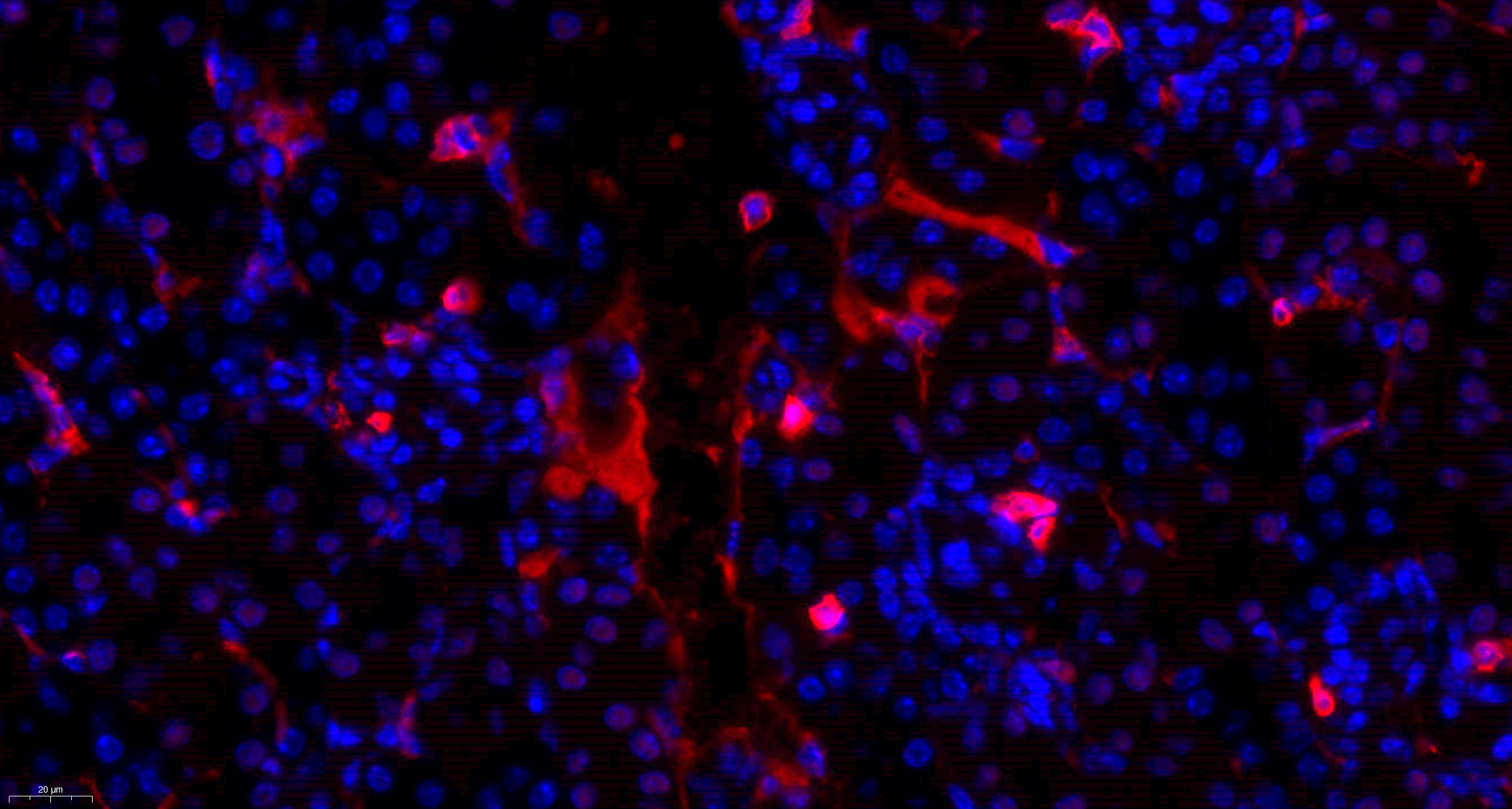

Supplement: Supplemental Information 7 [file peerj-13-20224-s007.zip › FIGURE6/FIG-6J/ACHN--PTGS2/ACHN/HBr.jpg]

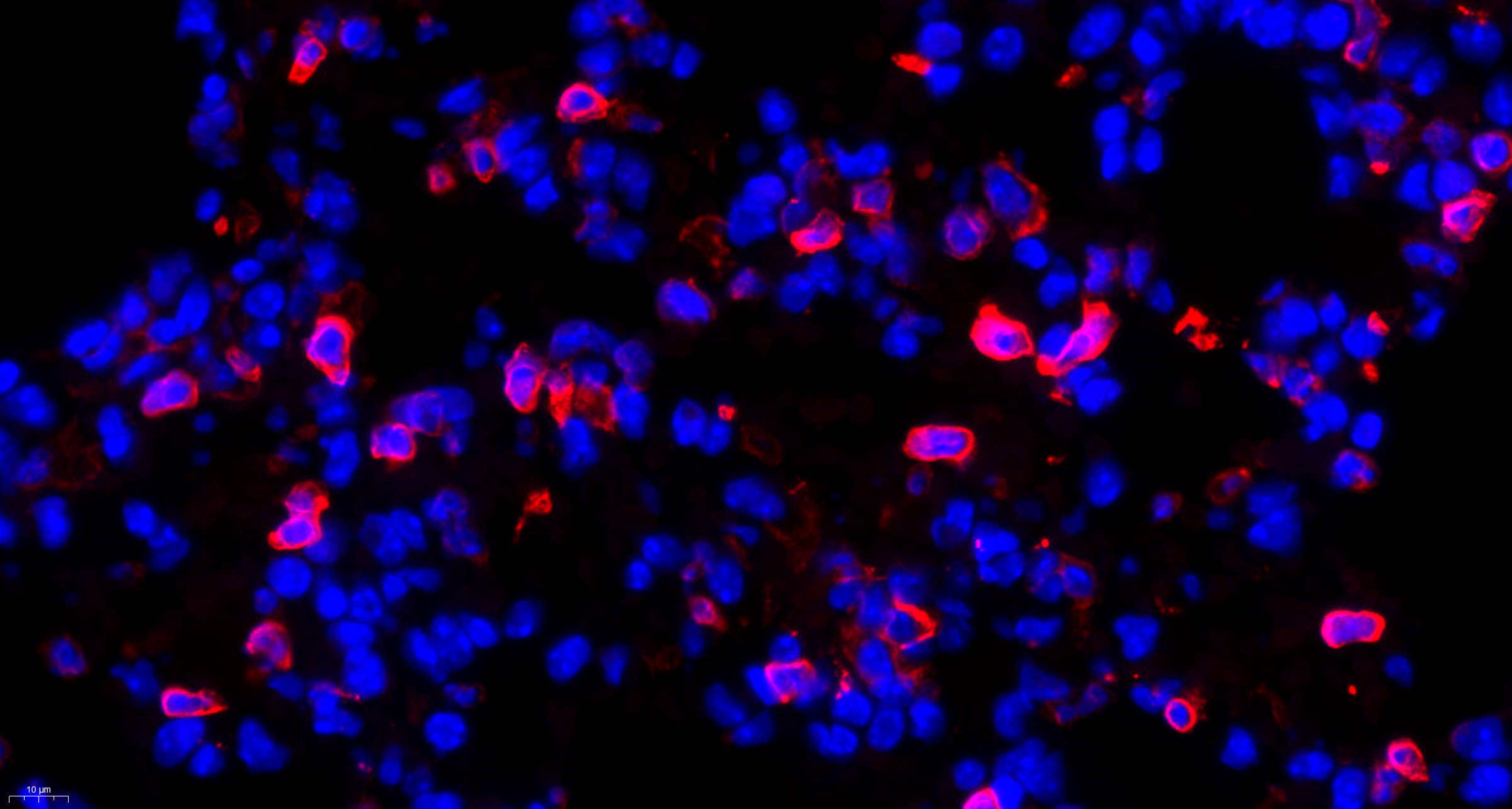

Supplement: Supplemental Information 7 [file peerj-13-20224-s007.zip › FIGURE6/FIG-6J/ACHN--SLC7A11/ACHN/DMSO.jpg]

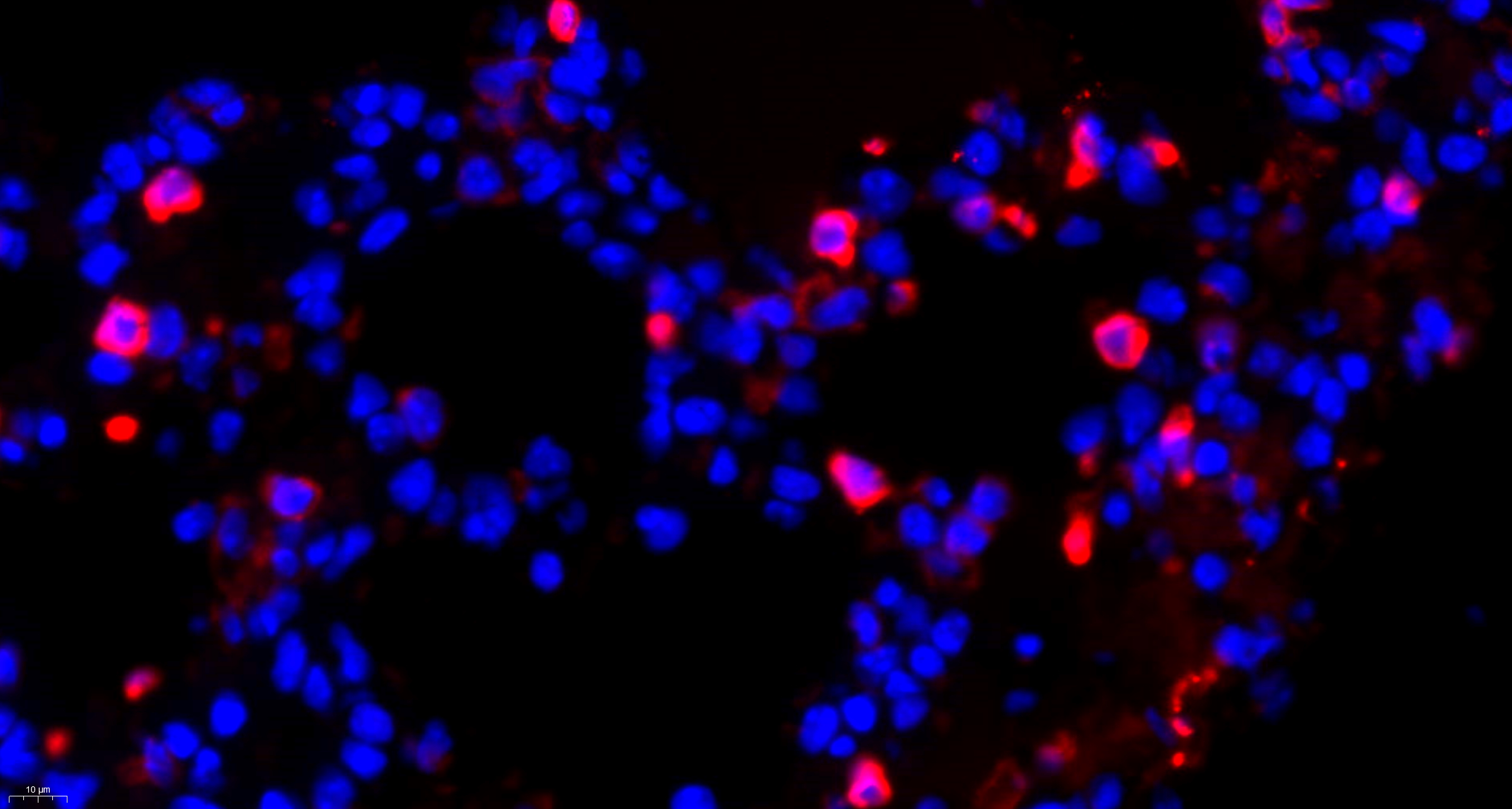

Supplement: Supplemental Information 7 [file peerj-13-20224-s007.zip › FIGURE6/FIG-6J/ACHN--SLC7A11/ACHN/HBR+NFE.jpg]
