# Supplementary material for: NAT10 inhibits ferroptosis and promotes the progression of renal clear cell carcinoma by regulating the NFE2L1-GPX4 signaling pathway
Source: PeerJ. 2025 Oct 31;13:e20224. doi: 10.7717/peerj.20224 (PMC12581918; doi:10.7717/peerj.20224)
Supplement: Supplemental Information 4 [file peerj-13-20224-s004.zip › FIGURE3/Fig.3A.pdf]

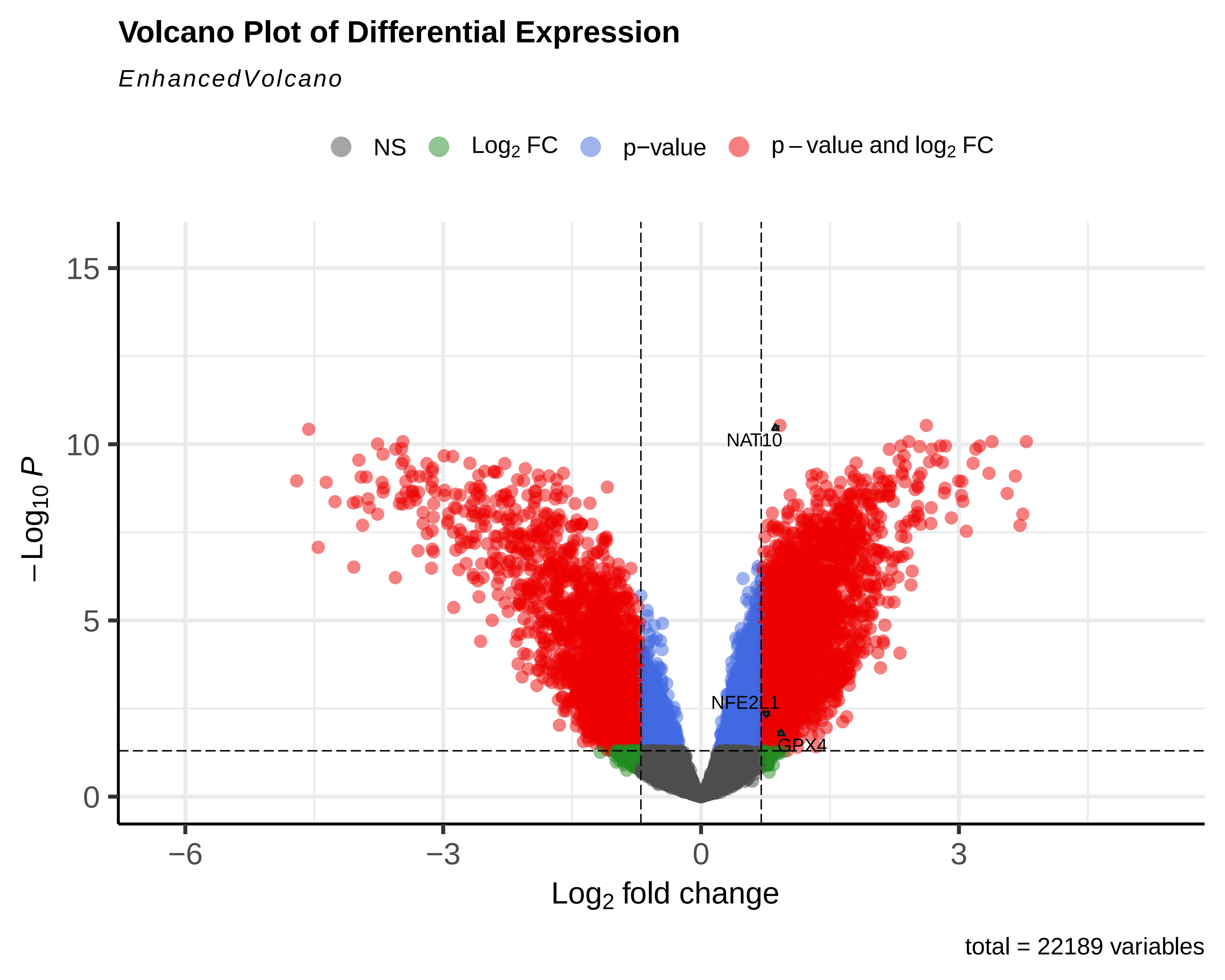

Volcano Plot of Differential Expression
EnhancedVolcano
NS
Log2 FC
p−value
p – value and log2 FC
15
10
5
0
−Log10 P
NAT10
NFE2L1
GPX4
−6
−3
0
3
Log2 fold change
total = 22189 variables
