# Supplementary figures and images for: NAT10 inhibits ferroptosis and promotes the progression of renal clear cell carcinoma by regulating the NFE2L1-GPX4 signaling pathway (part 2 of 2)
Source: PeerJ. 2025 Oct 31;13:e20224. doi: 10.7717/peerj.20224 (PMC12581918; doi:10.7717/peerj.20224)

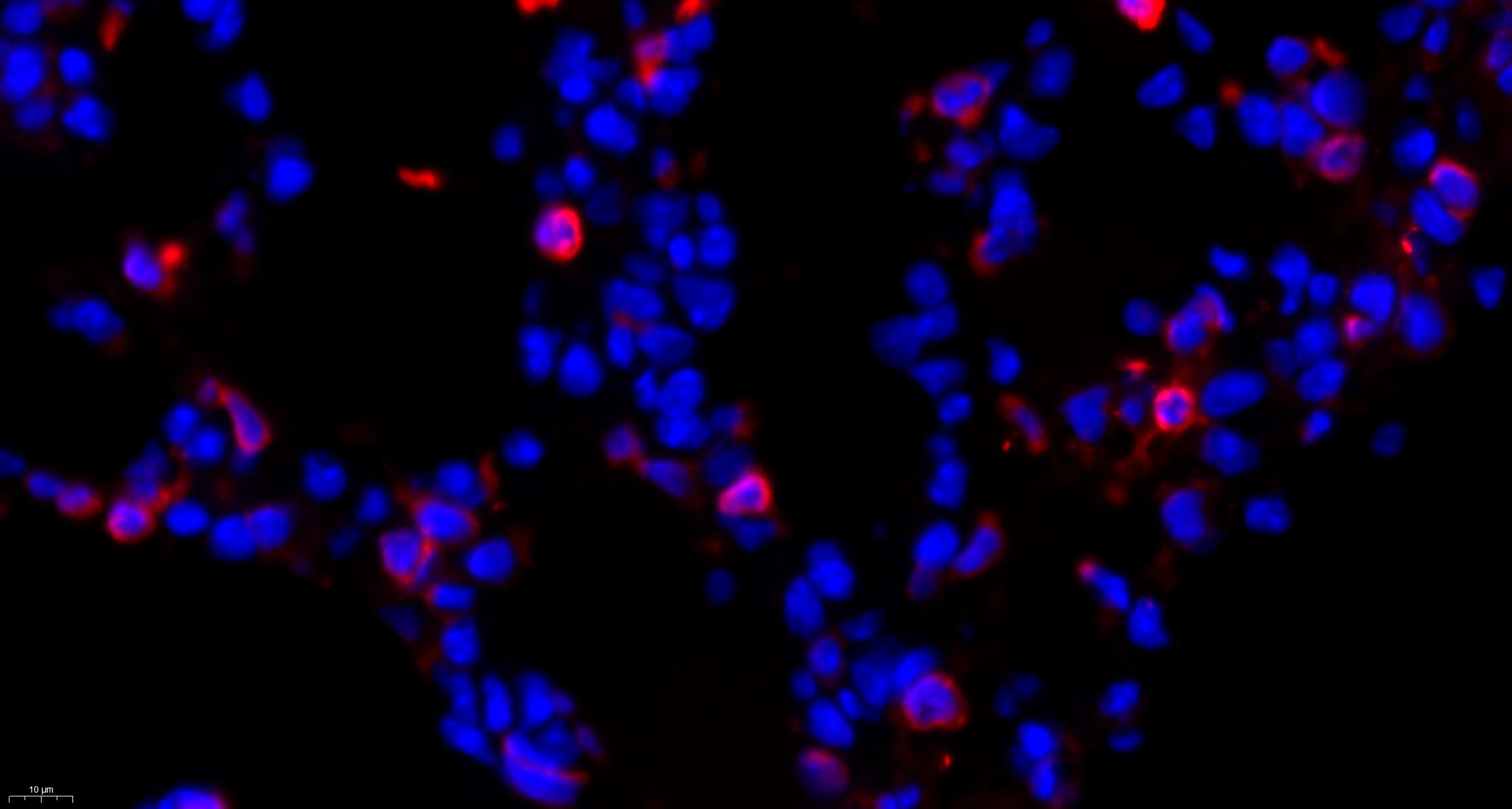

Supplement: Supplemental Information 7 [file peerj-13-20224-s007.zip › FIGURE6/FIG-6J/ACHN--SLC7A11/ACHN/HBr.jpg]

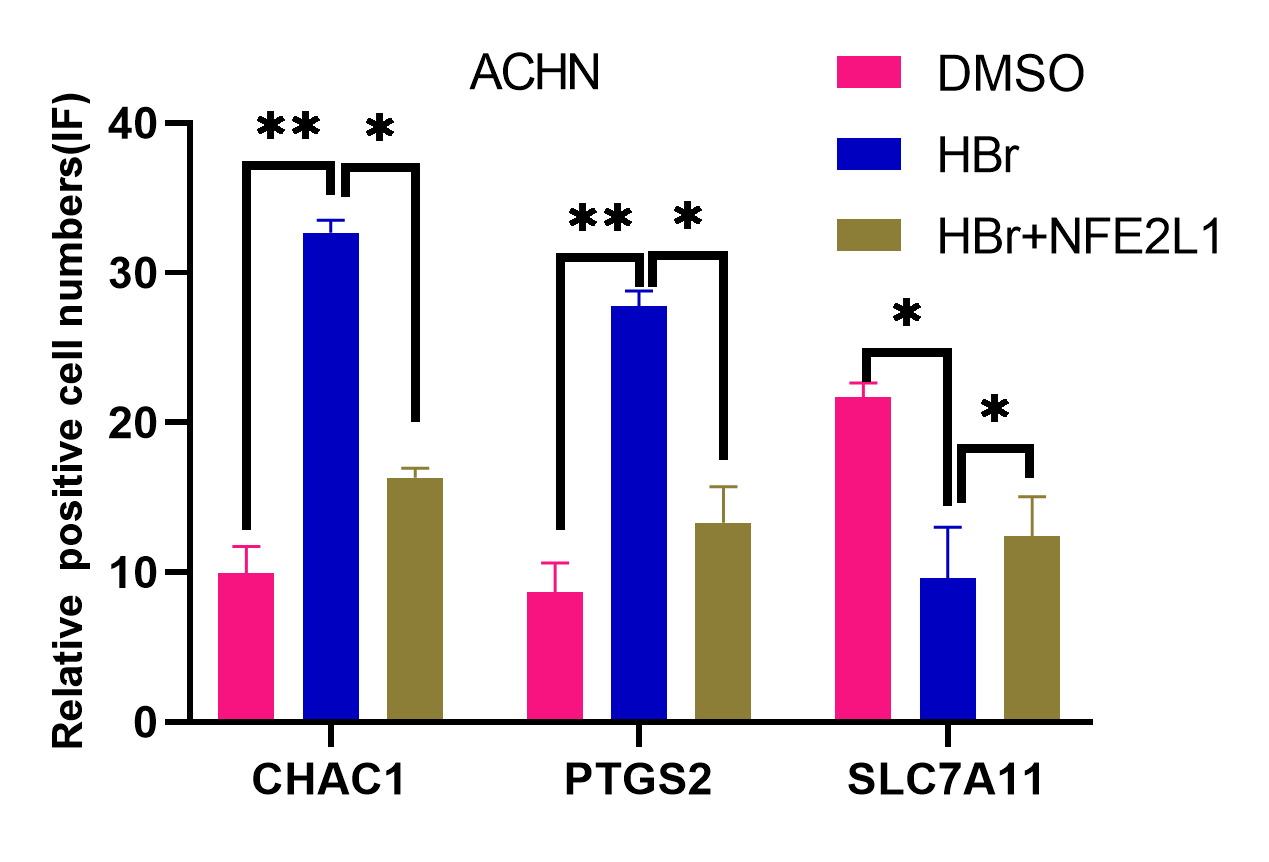

Supplement: Supplemental Information 7 [file peerj-13-20224-s007.zip › FIGURE6/FIG-6K/FIG-6K.tif]

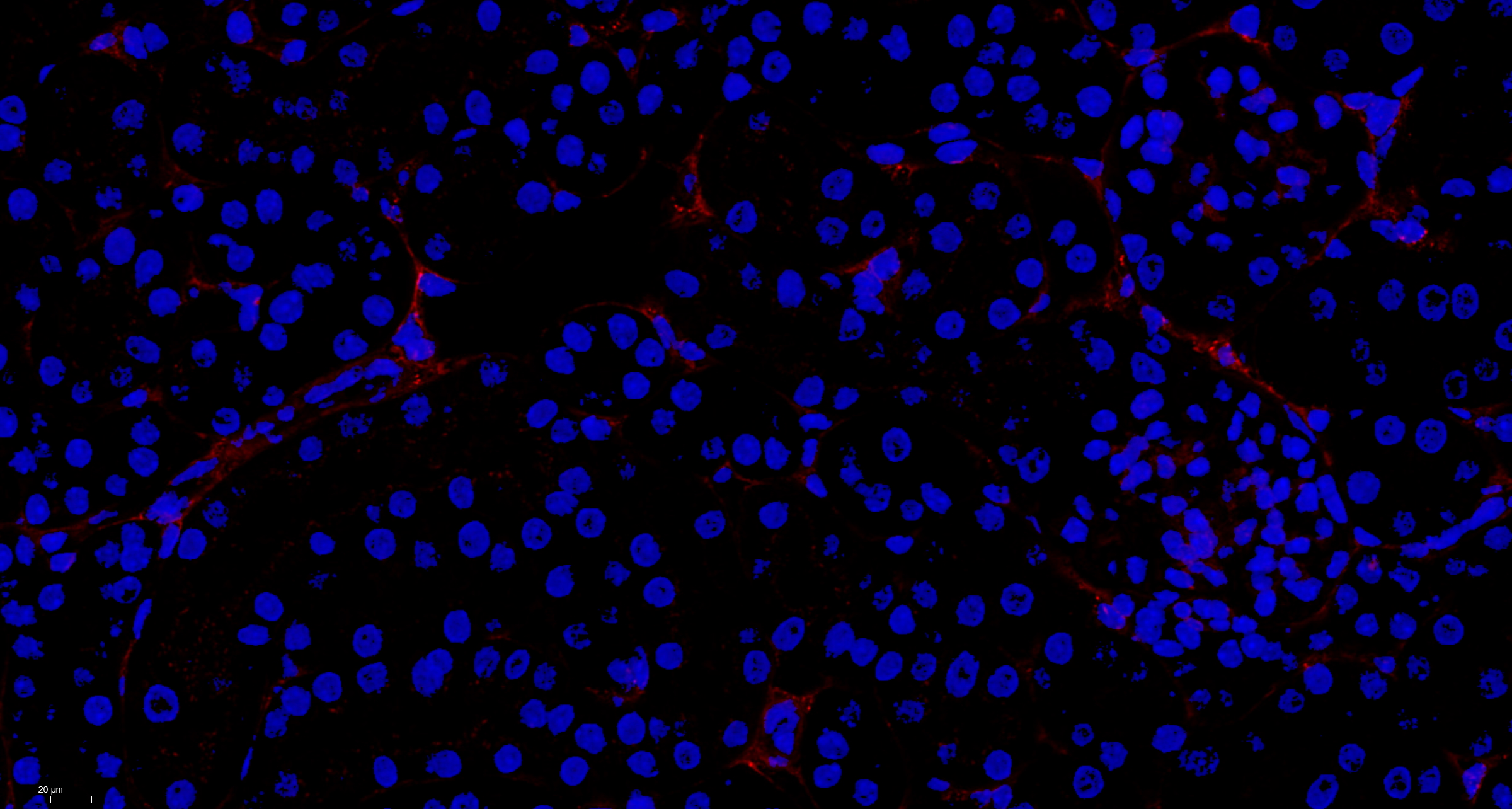

Supplement: Supplemental Information 7 [file peerj-13-20224-s007.zip › FIGURE6/FIG-6L/Caki1--CHAC1/CAK-1I/DMSO.jpg]

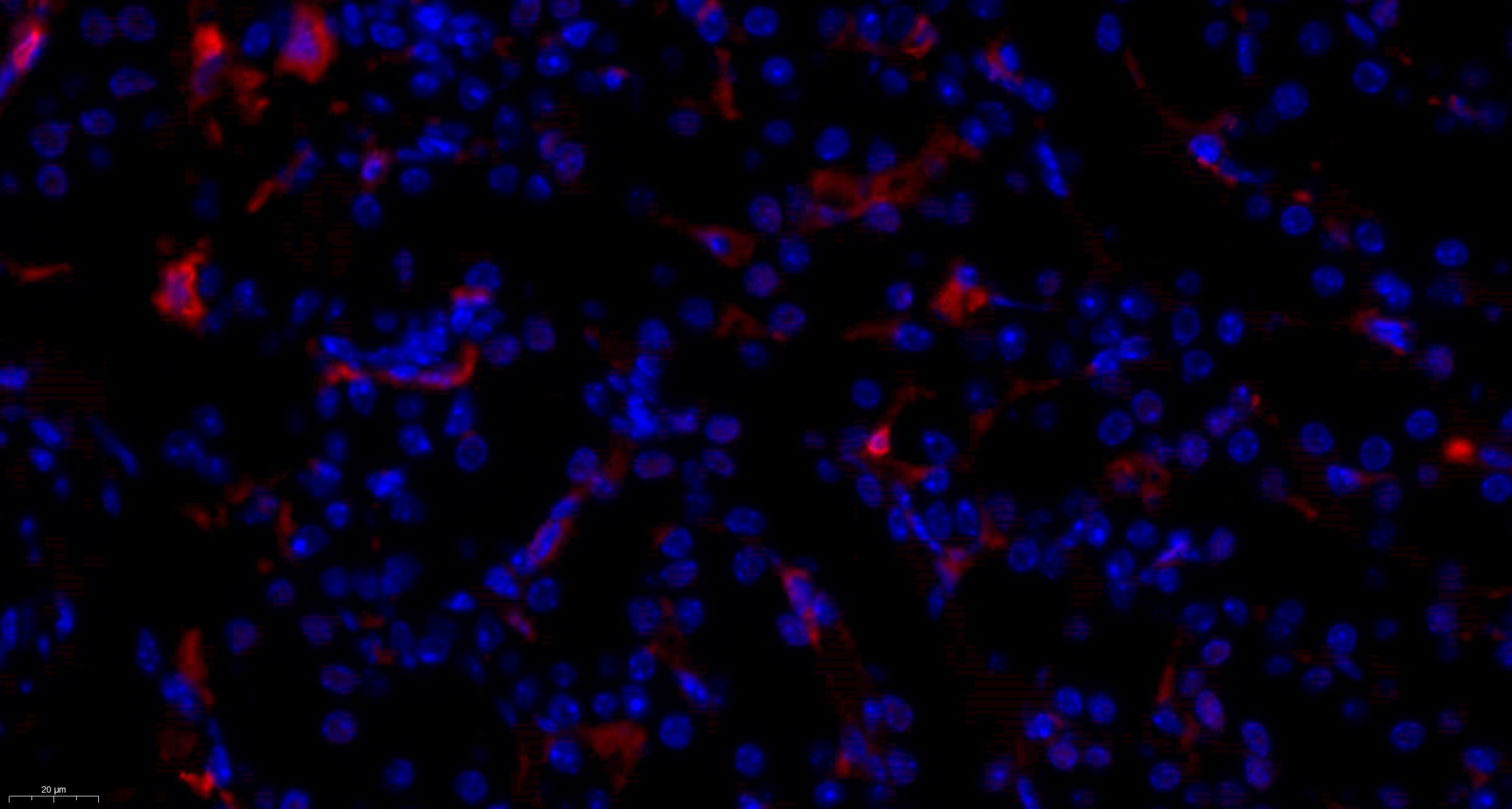

Supplement: Supplemental Information 7 [file peerj-13-20224-s007.zip › FIGURE6/FIG-6L/Caki1--CHAC1/CAK-1I/HBR+NFE.jpg]

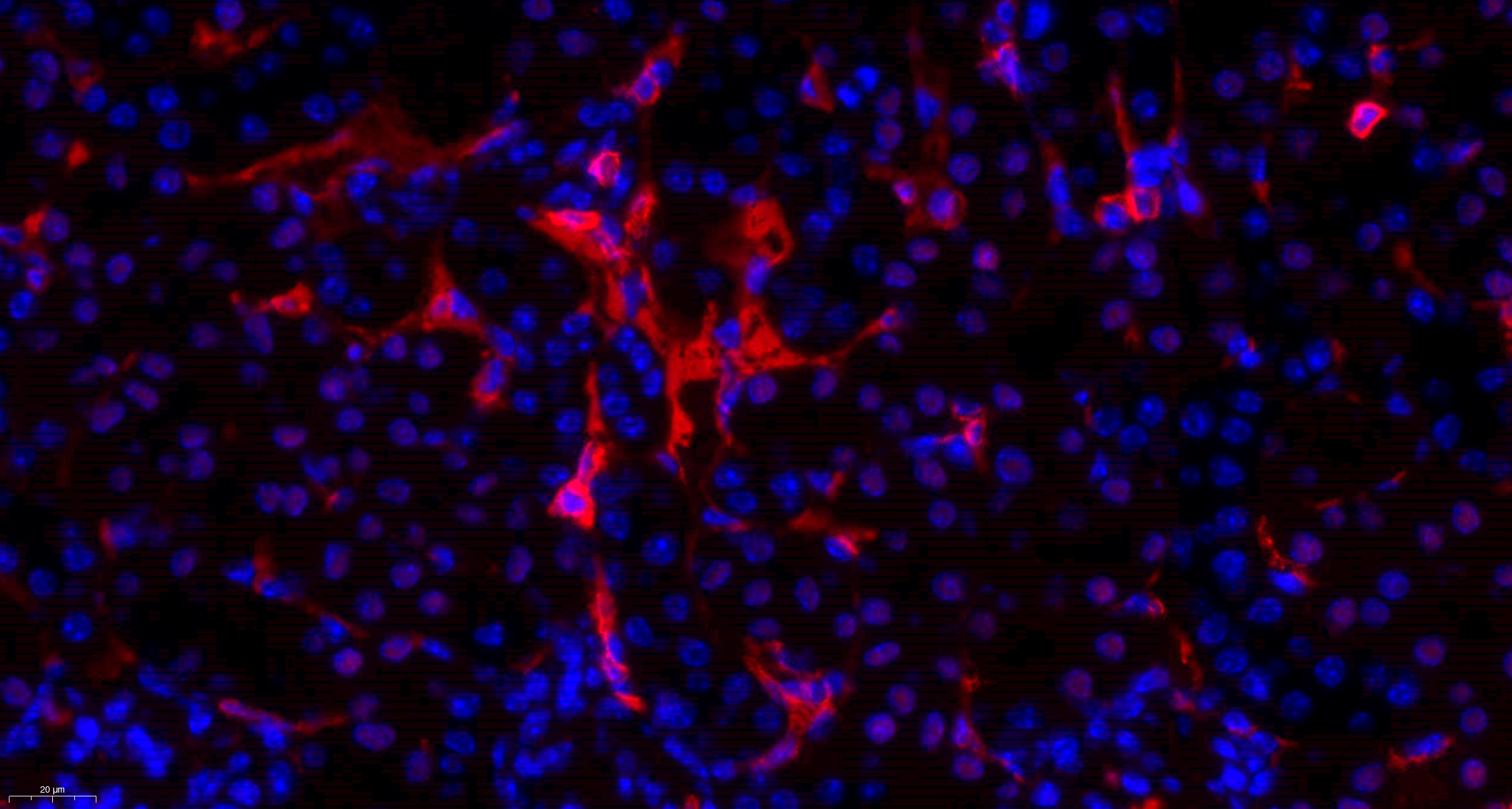

Supplement: Supplemental Information 7 [file peerj-13-20224-s007.zip › FIGURE6/FIG-6L/Caki1--CHAC1/CAK-1I/HBr.jpg]

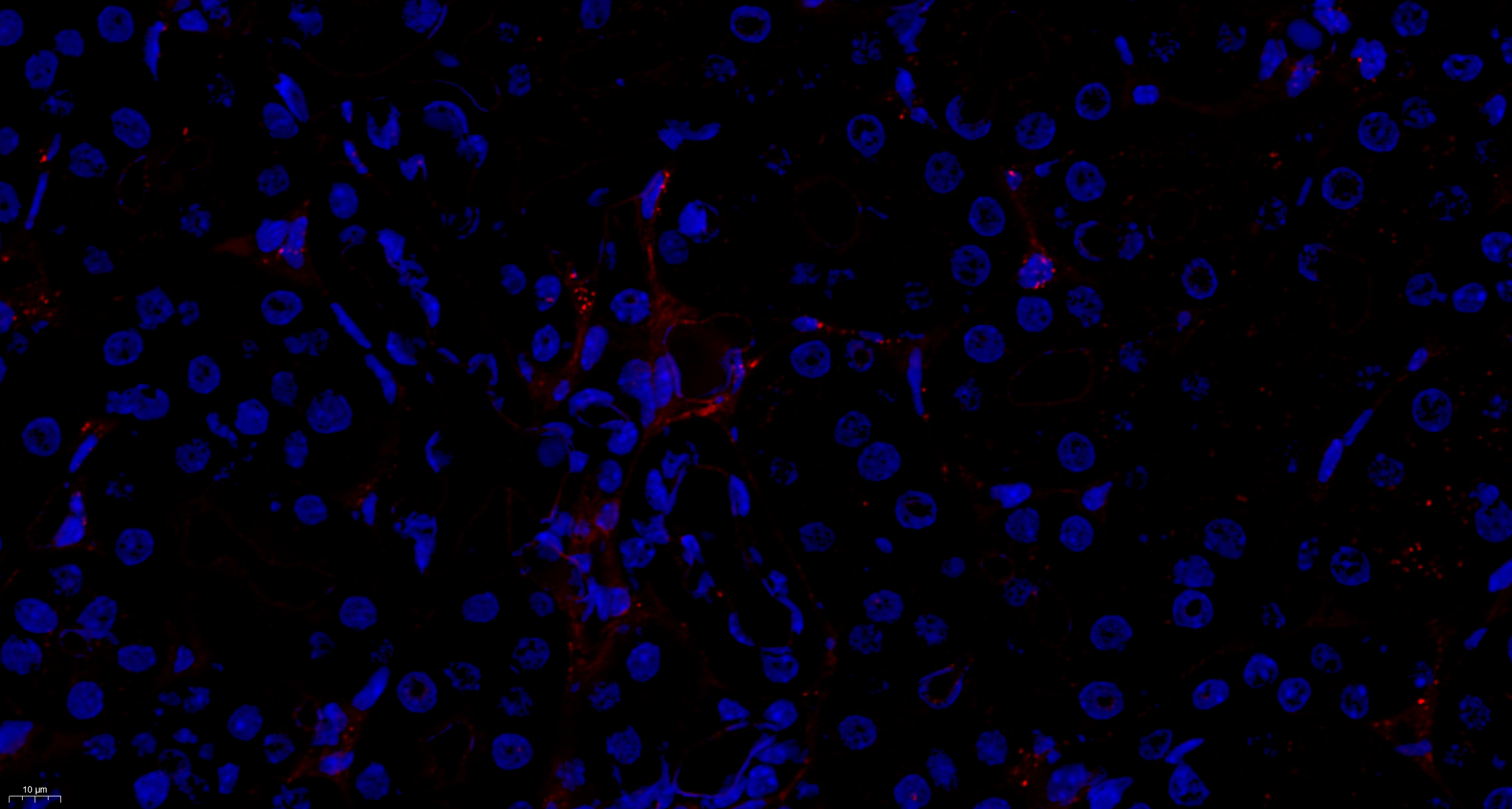

Supplement: Supplemental Information 7 [file peerj-13-20224-s007.zip › FIGURE6/FIG-6L/Caki1--PTGS2/CAK-1I/DMSO.jpg]

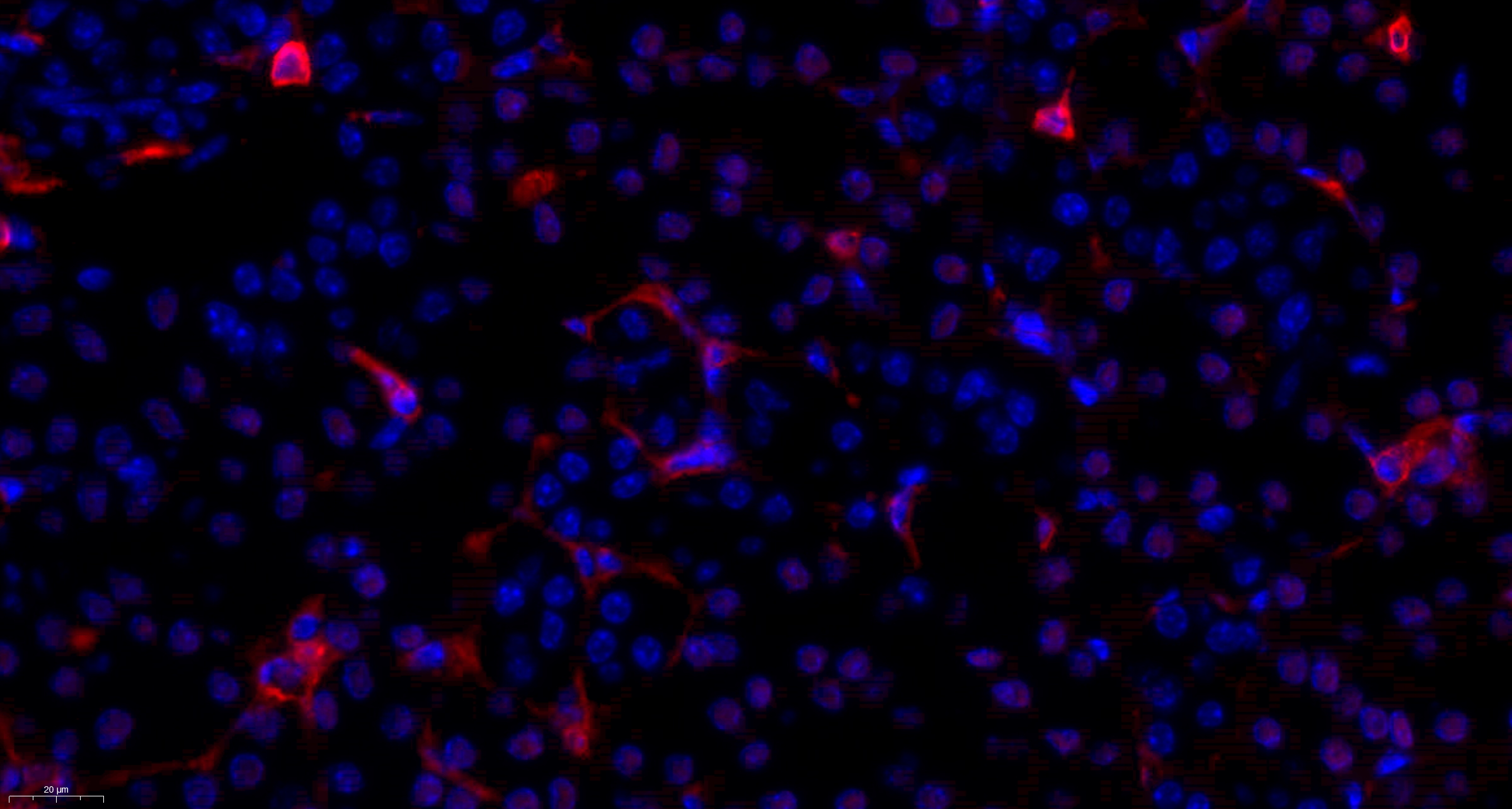

Supplement: Supplemental Information 7 [file peerj-13-20224-s007.zip › FIGURE6/FIG-6L/Caki1--PTGS2/CAK-1I/HBR+NFE.jpg]

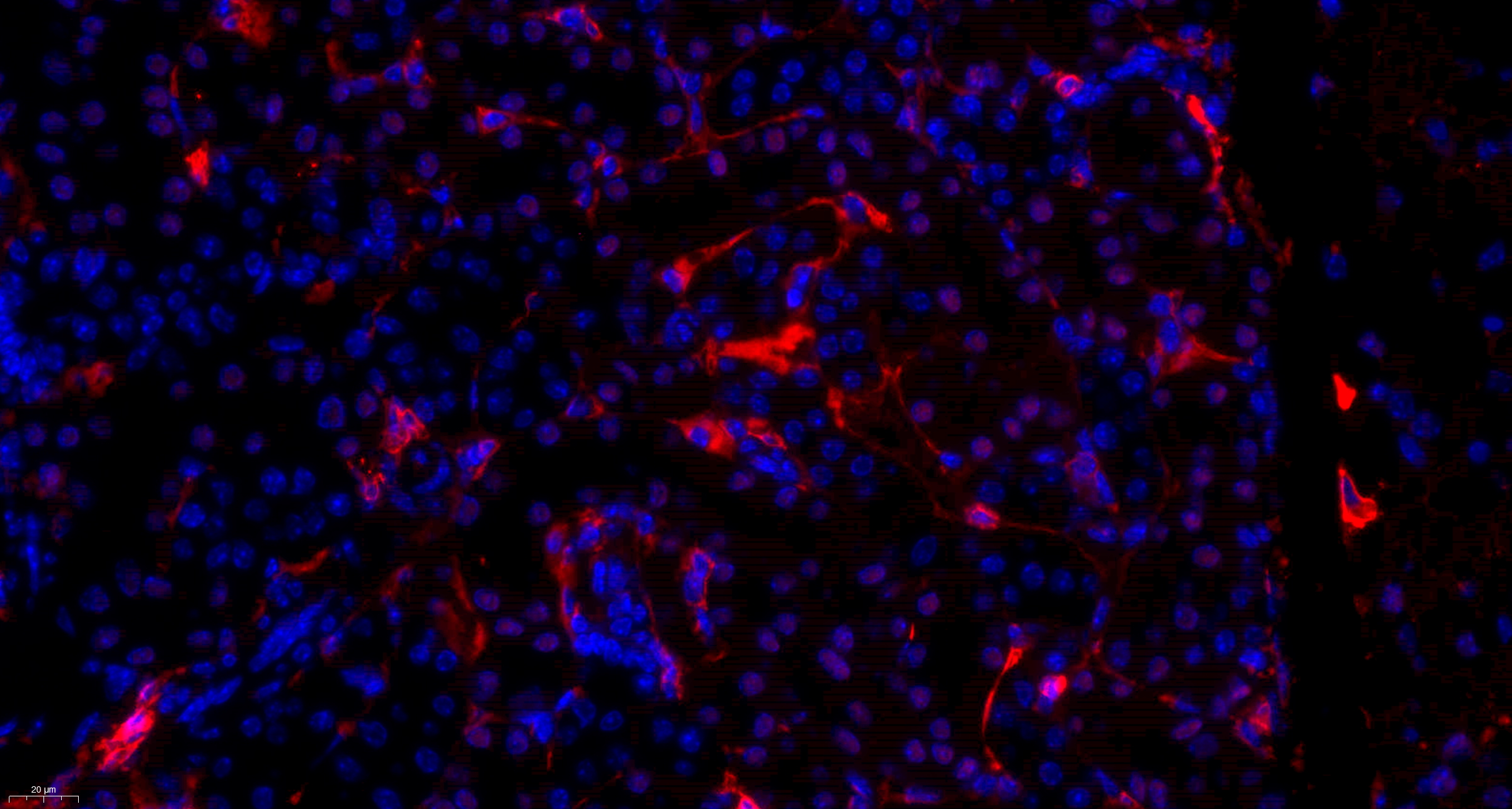

Supplement: Supplemental Information 7 [file peerj-13-20224-s007.zip › FIGURE6/FIG-6L/Caki1--PTGS2/CAK-1I/HBr.jpg]

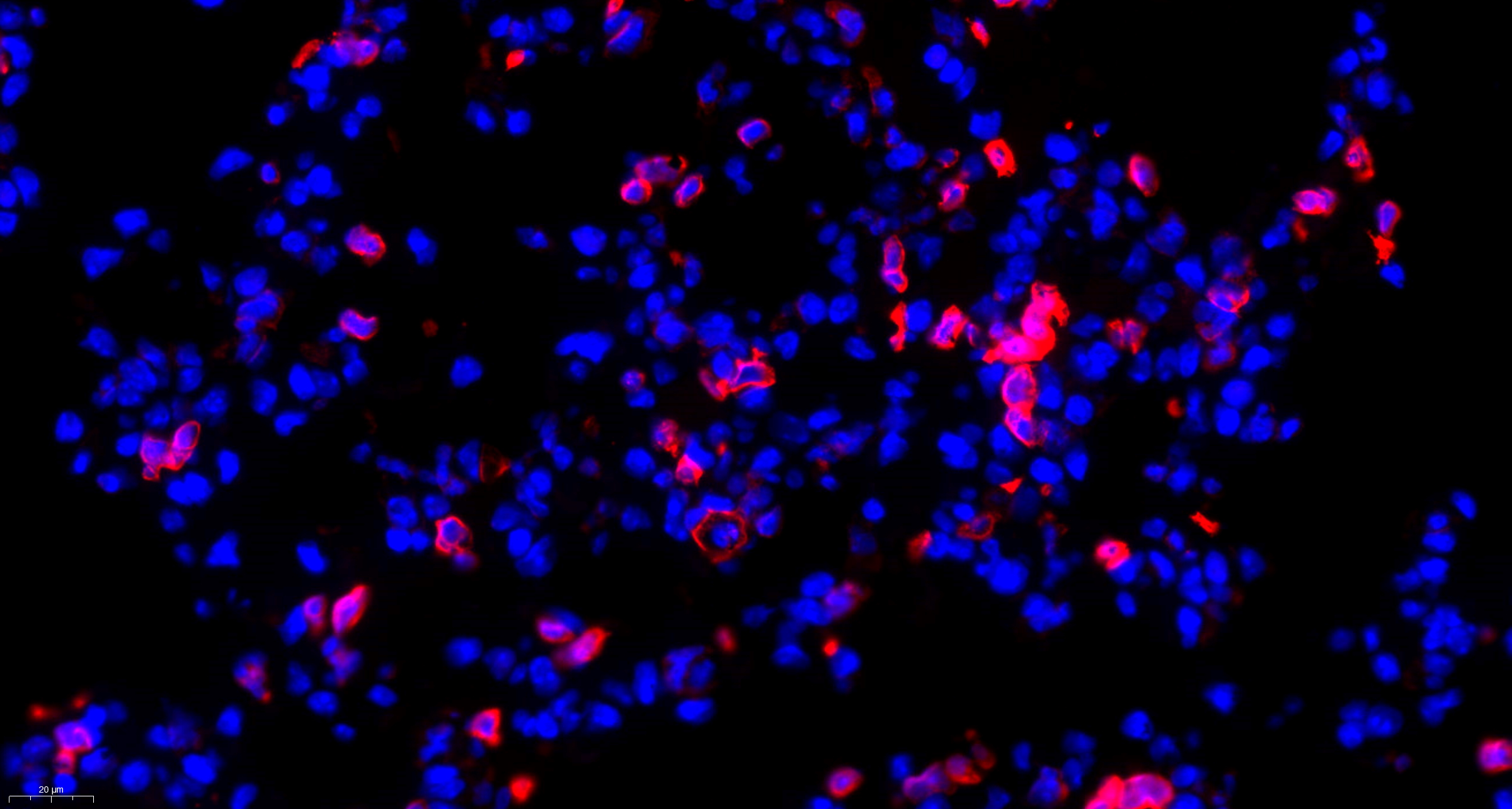

Supplement: Supplemental Information 7 [file peerj-13-20224-s007.zip › FIGURE6/FIG-6L/Caki1--SLC7A11/CAK-1I/DMSO.jpg]

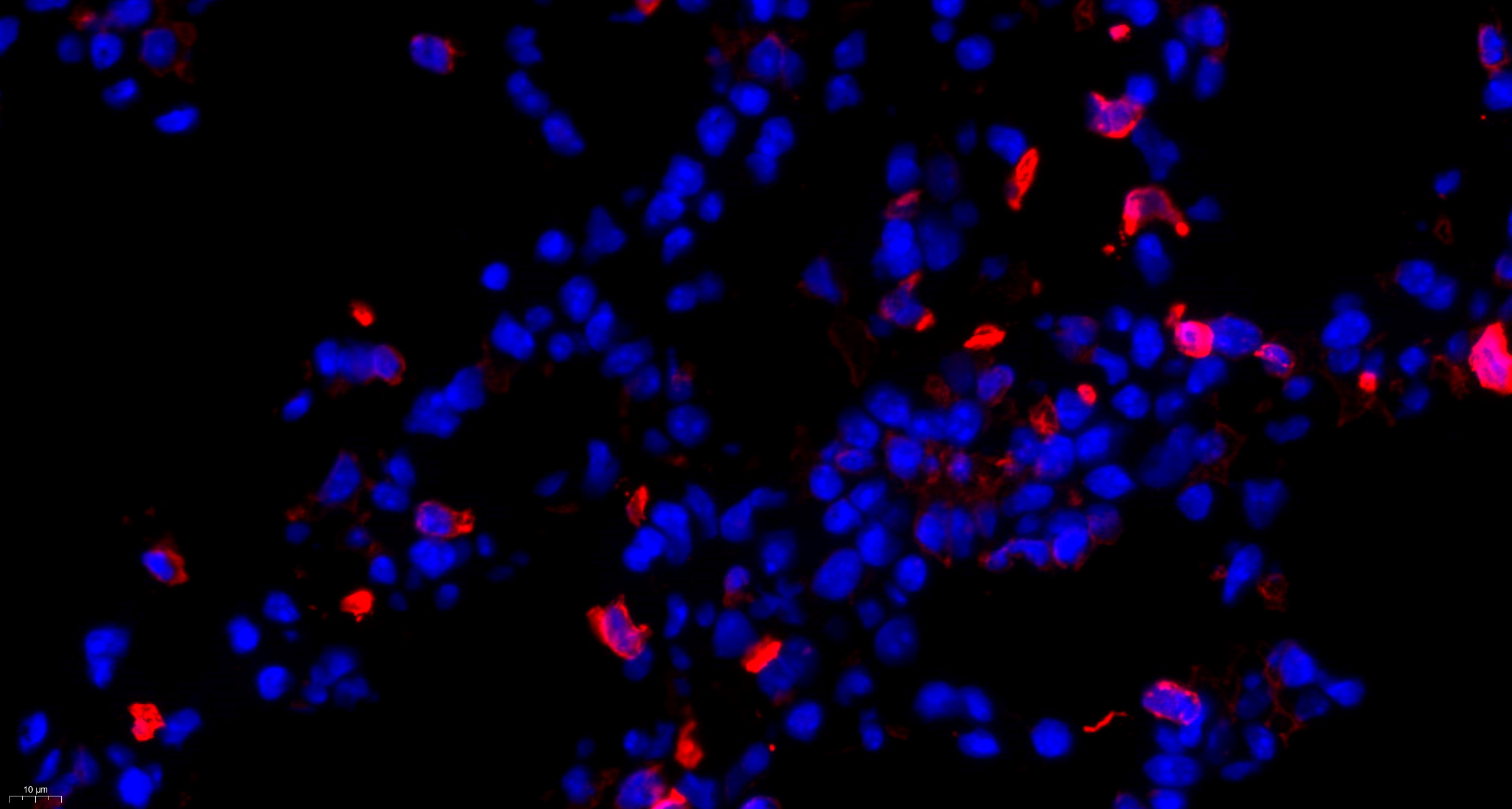

Supplement: Supplemental Information 7 [file peerj-13-20224-s007.zip › FIGURE6/FIG-6L/Caki1--SLC7A11/CAK-1I/HBR+NFE.jpg]

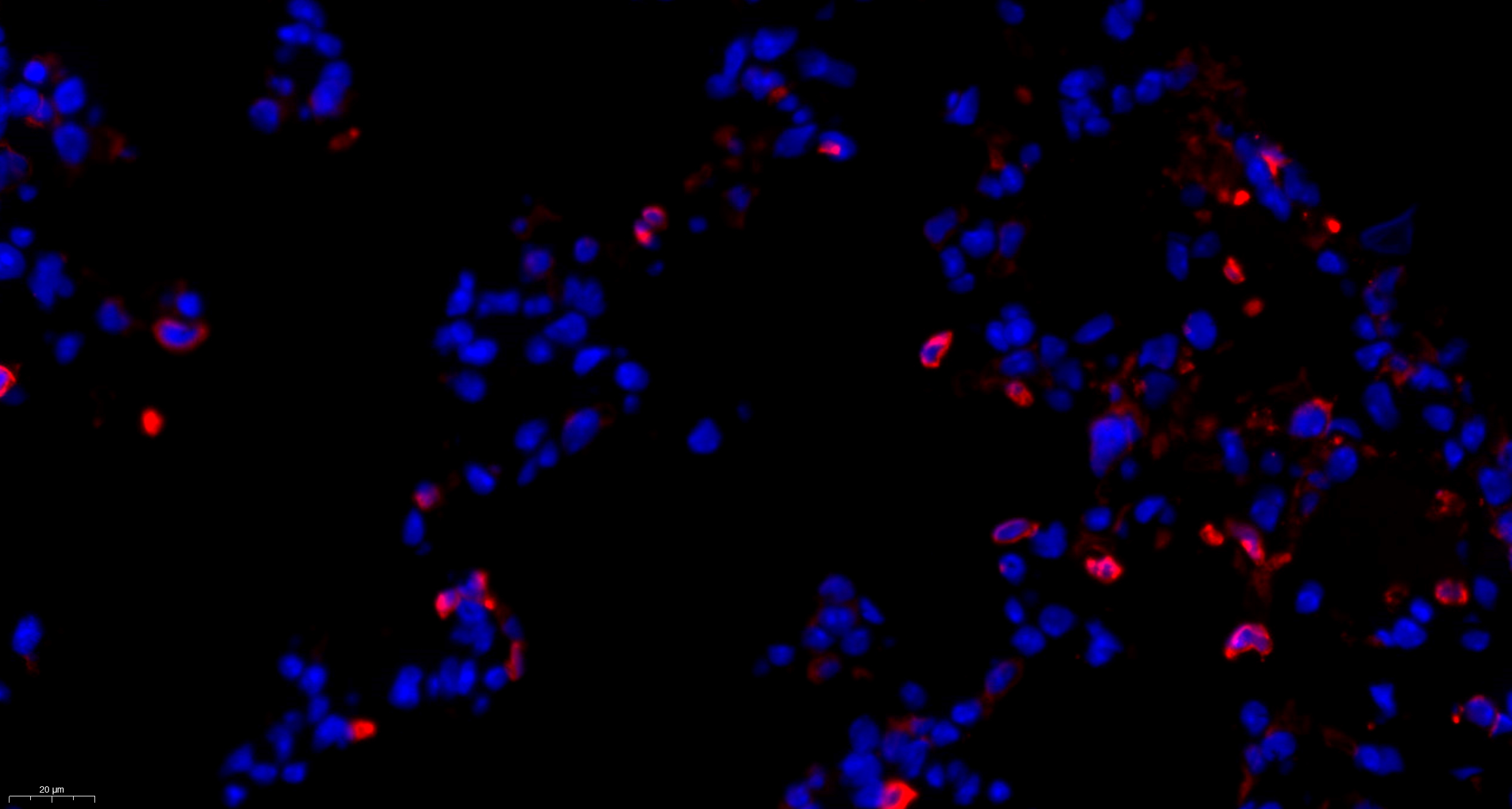

Supplement: Supplemental Information 7 [file peerj-13-20224-s007.zip › FIGURE6/FIG-6L/Caki1--SLC7A11/CAK-1I/HBr.jpg]

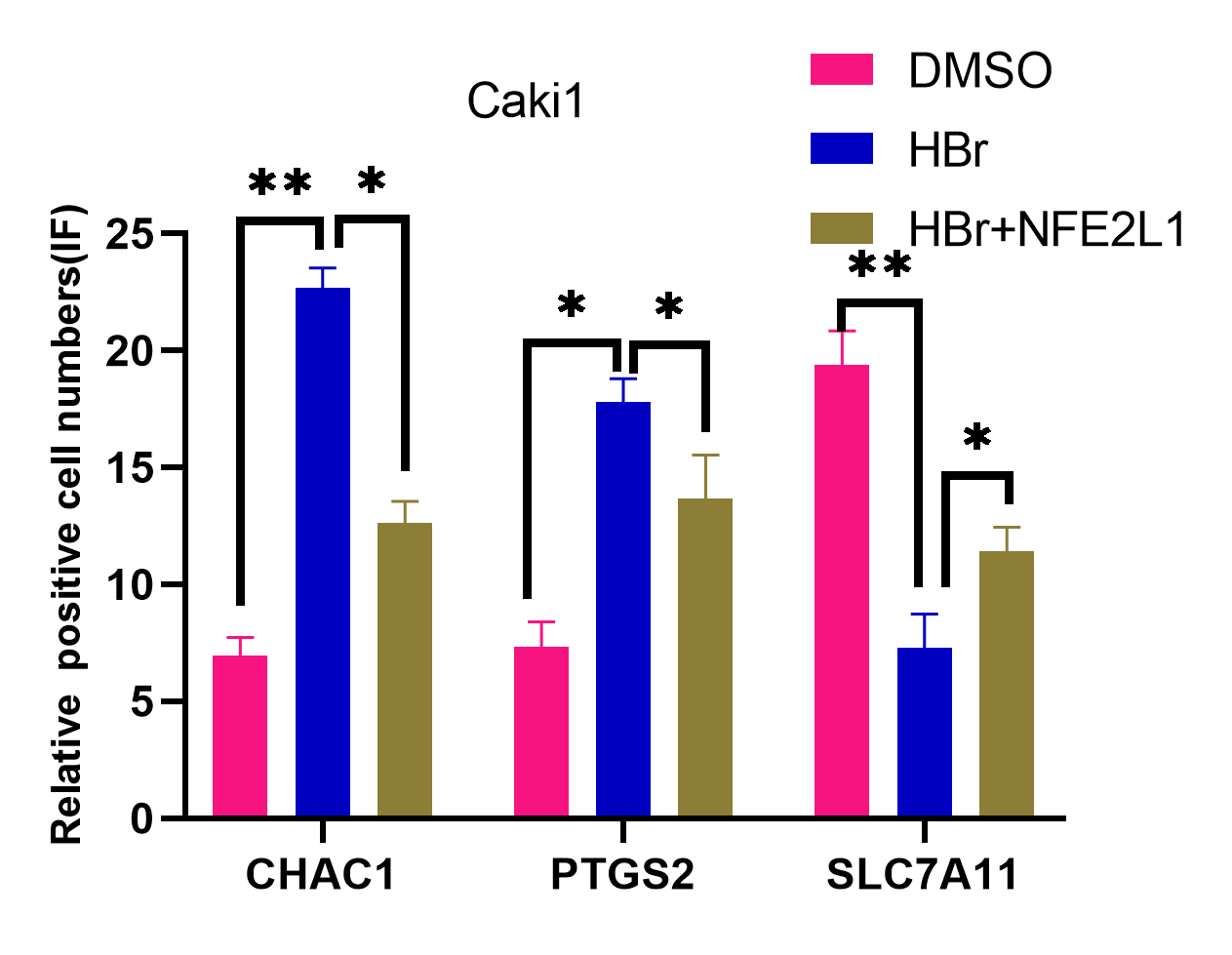

Supplement: Supplemental Information 7 [file peerj-13-20224-s007.zip › FIGURE6/FIG-6M/FIG-6M.tif]

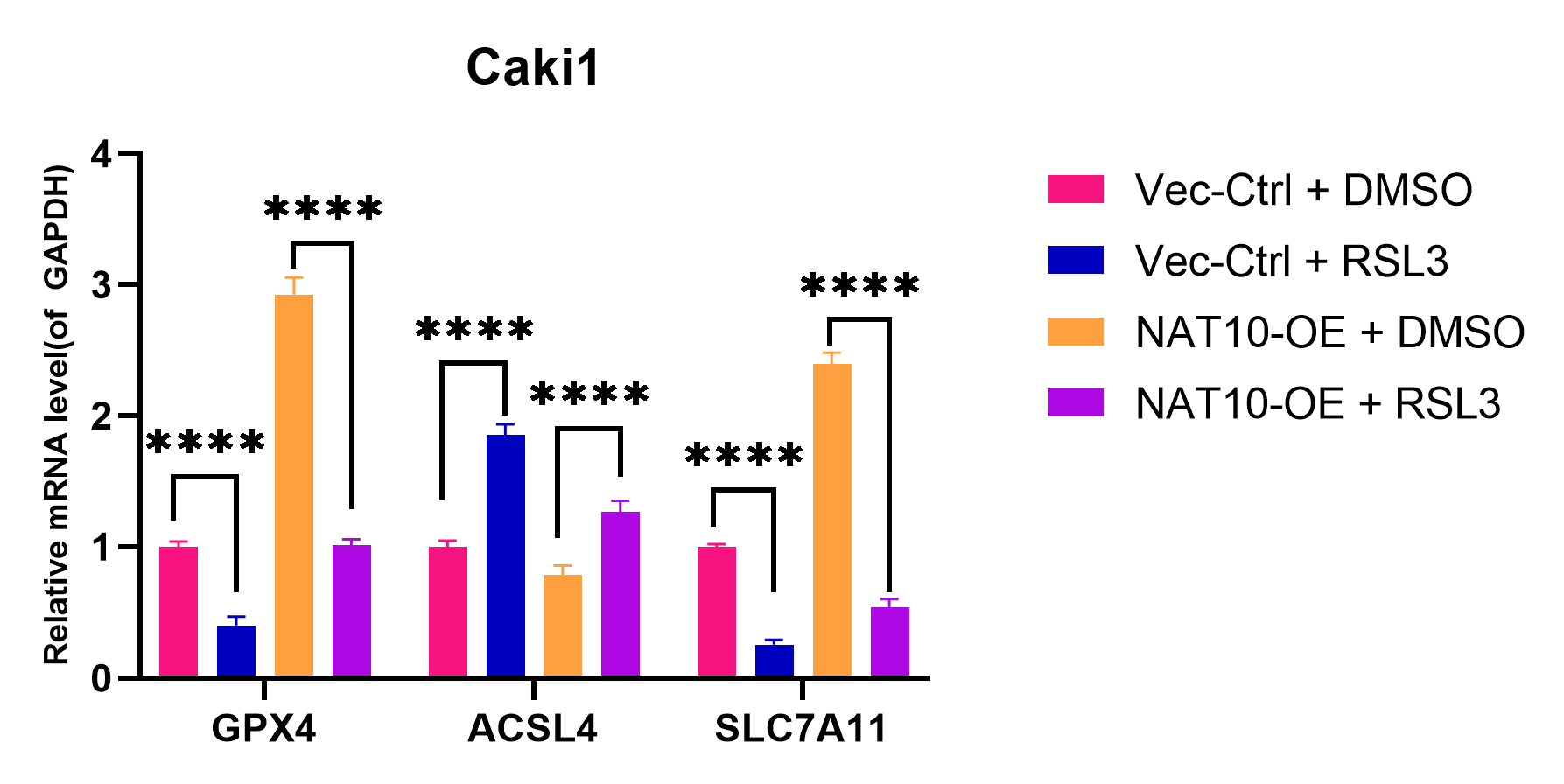

Supplement: Supplemental Information 8 [file peerj-13-20224-s008.zip › FIGURE7/FIG-7A/Fig.7A.tif]

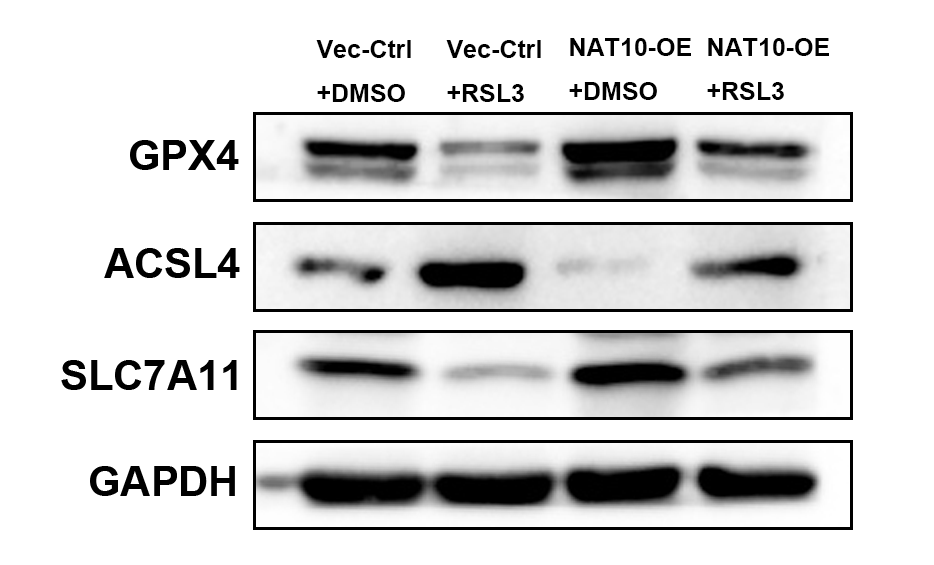

Supplement: Supplemental Information 8 [file peerj-13-20224-s008.zip › FIGURE7/FIG-7B-7C/Fig.7B.tif]

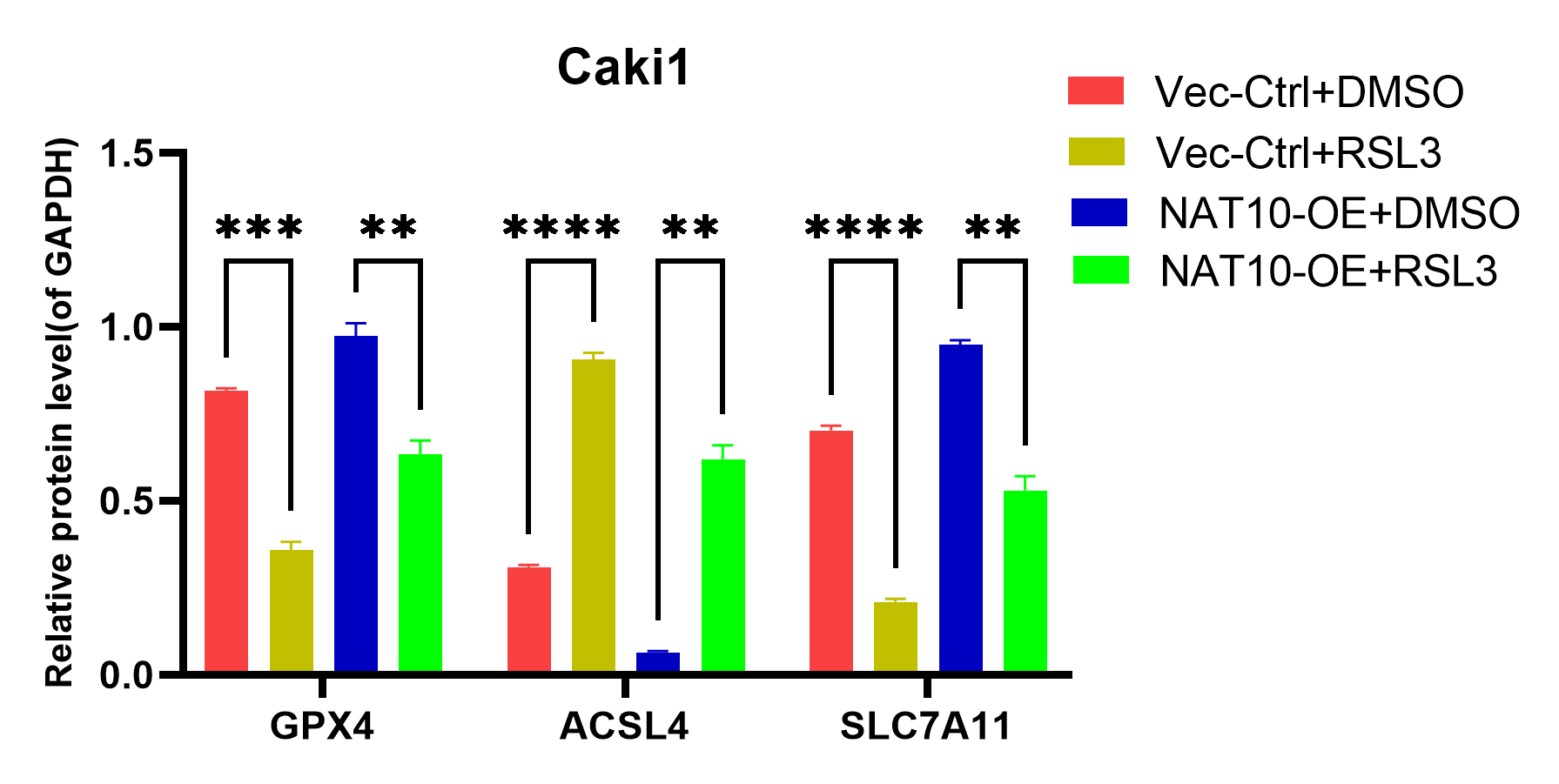

Supplement: Supplemental Information 8 [file peerj-13-20224-s008.zip › FIGURE7/FIG-7B-7C/Fig.7C.tif]

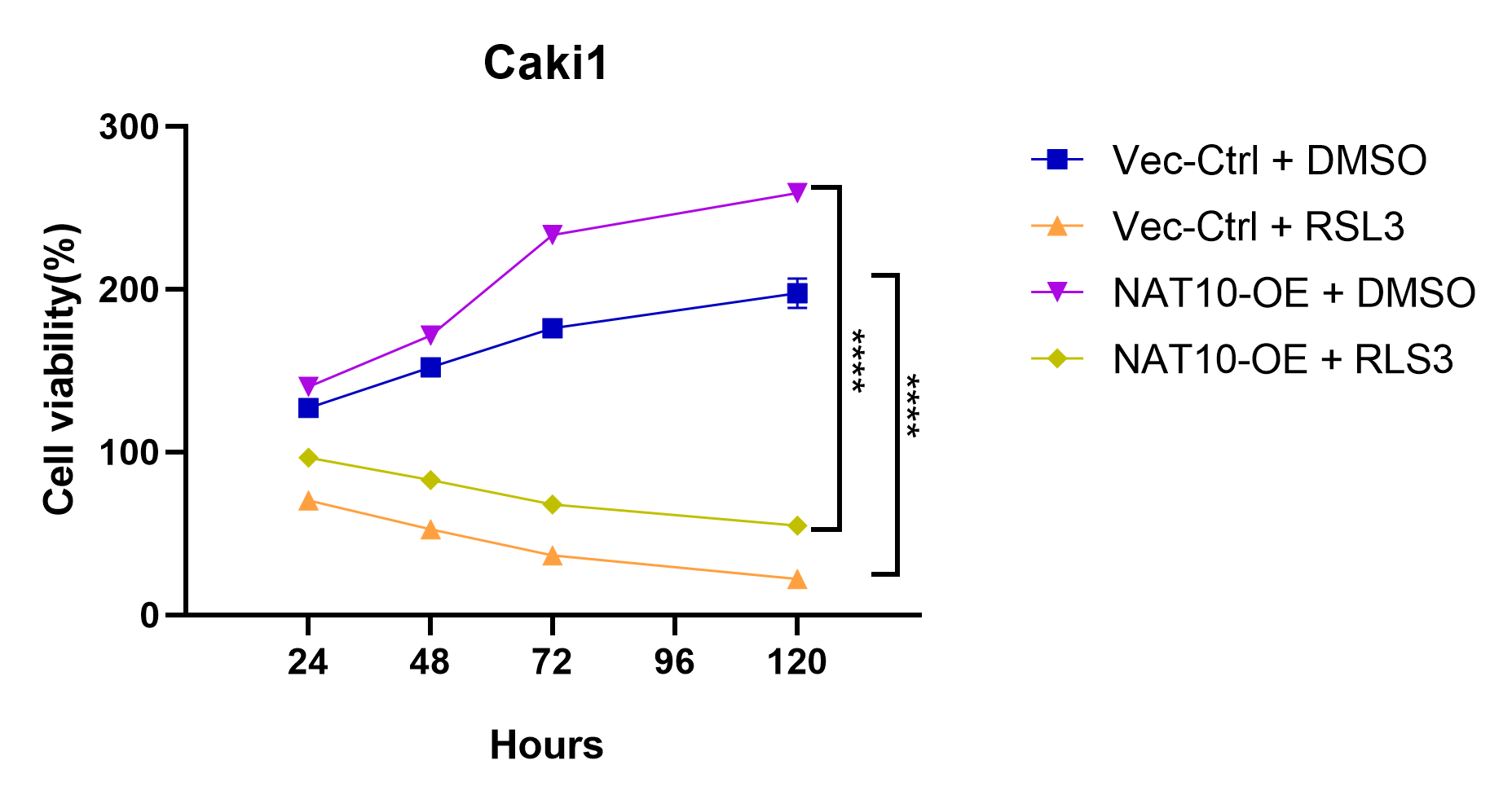

Supplement: Supplemental Information 8 [file peerj-13-20224-s008.zip › FIGURE7/FIG-7D/Fig.7D.tif]

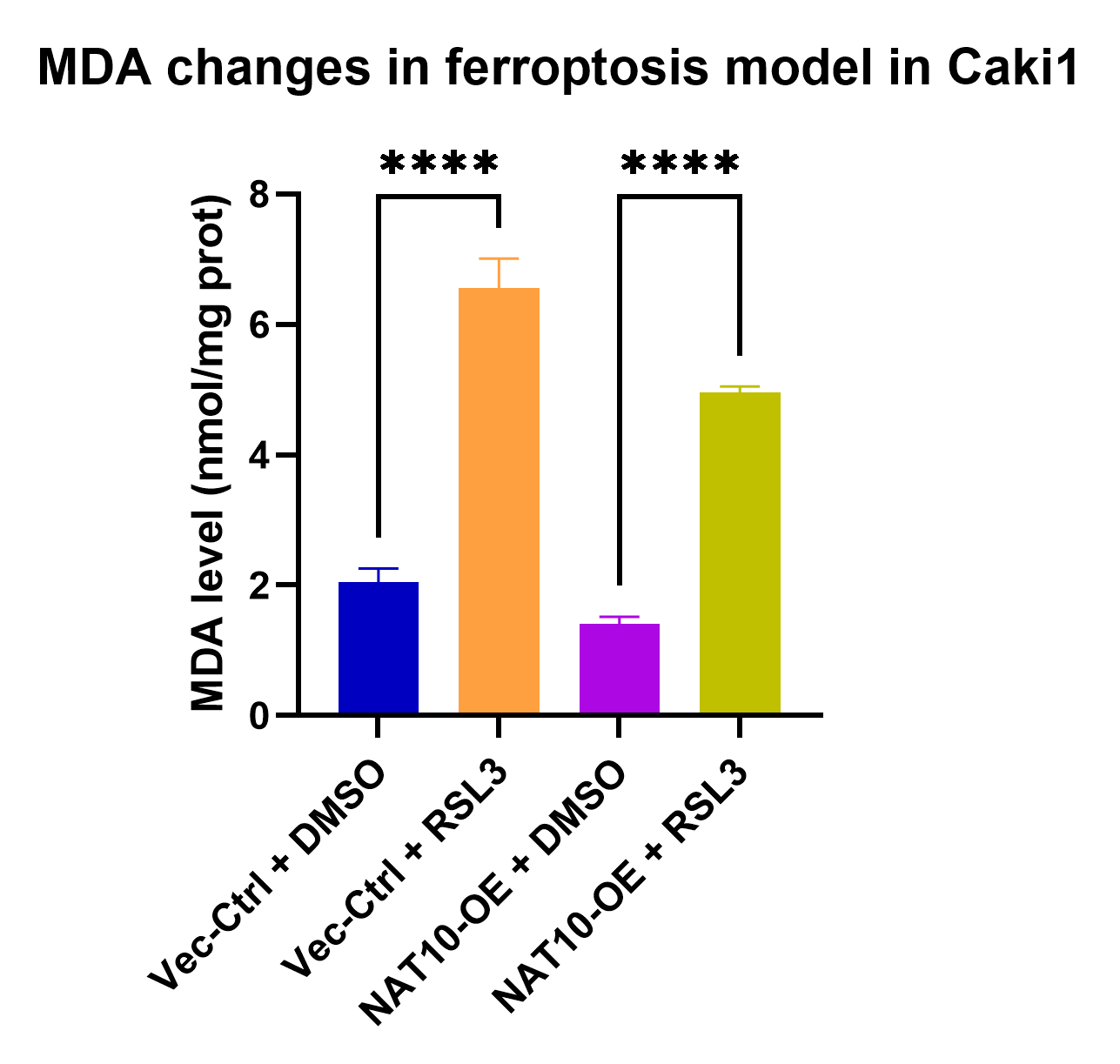

Supplement: Supplemental Information 8 [file peerj-13-20224-s008.zip › FIGURE7/FIG-7E/Fig.7E.tif]

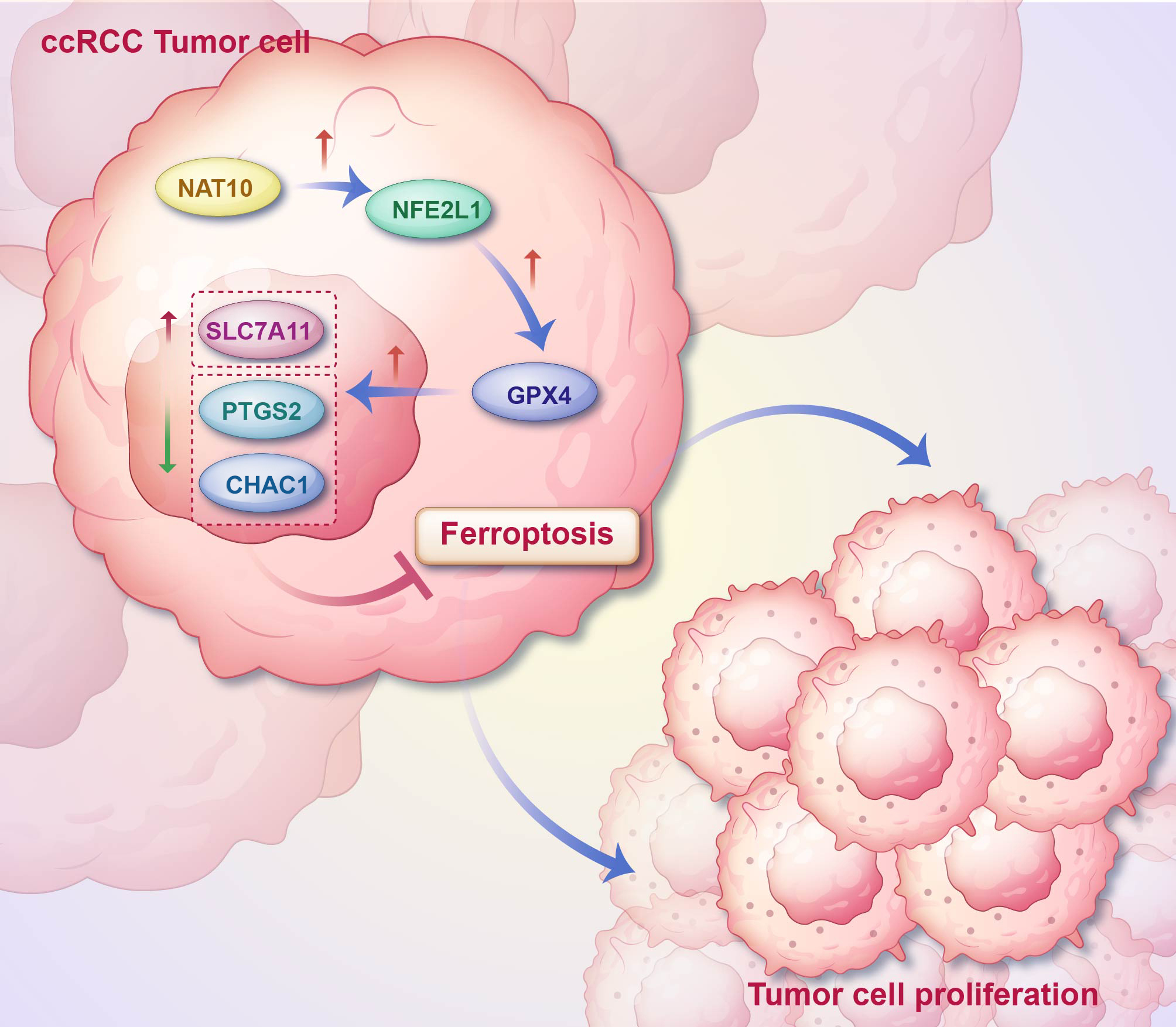

Supplement: Supplemental Information 9 [file peerj-13-20224-s009.jpg]
